# Molecular evolution and population genetics of glutamate decarboxylase acid resistance pathway in lactic acid bacteria

**DOI:** 10.3389/fgene.2023.1027156

**Published:** 2023-01-26

**Authors:** Efe Sezgin, Burcu Tekin

**Affiliations:** ^1^ Department of Food Engineering, Izmir Institute of Technology, Urla, Izmir, Turkey; ^2^ Biotechnology Interdisciplinary Program, Izmir Institute of Technology, Urla, Izmir, Turkey

**Keywords:** acid resistance pathway, glutamate decarboxylase, population genetics, molecular evolution, selection, lactic acid bacteria, glutamate/GABA antiporter, population differentiation

## Abstract

Glutamate decarboxylase (GAD) pathway (GDP) is a major acid resistance mechanism enabling microorganisms’ survival in low pH environments. We aimed to study the molecular evolution and population genetics of GDP in Lactic Acid Bacteria (LAB) to understand evolutionary processes shaping adaptation to acidic environments comparing species where the GDP genes are organized in an operon structure (*Levilactobacillus brevis*) versus lack of an operon structure (*Lactiplantibacillus plantarum*). Within species molecular population genetic analyses of GDP genes in *L. brevis* and *L. plantarum* sampled from diverse fermented food and other environments showed abundant synonymous and non-synonymous nucleotide diversity, mostly driven by low frequency changes, distributed throughout the coding regions for all genes in both species. GAD genes showed higher level of replacement polymorphism compared to transporter genes (*gadC* and *YjeM*) for both species, and GAD genes that are outside of an operon structure showed even higher level of replacement polymorphism. Population genetic tests suggest negative selection against replacement changes in all genes. Molecular structure and amino acid characteristics analyses showed that in none of the GDP genes replacement changes alter 3D structure or charge distribution supporting negative selection against non-conservative amino acid changes. Phylogenetic and between species divergence analyses suggested adaptive protein evolution on GDP genes comparing phylogenetically distant species, but conservative evolution comparing closely related species. GDP genes within an operon structure showed slower molecular evolution and higher conservation. All GAD and transporter genes showed high codon usage bias in examined LAB species suggesting high expression and utilization of acid resistance genes. Substantial discordances between species, GAD, and transporter gene tree topologies were observed suggesting molecular evolution of GDP genes do not follow speciation events. Distribution of operon structure on the species tree suggested multiple independent gain or loss of operon structure in LABs. In conclusion, GDP genes in LABs exhibit a dynamic molecular evolutionary history shaped by gene loss, gene transfer, negative and positive selection to maintain its active role in acid resistance mechanism, and enable organisms to thrive in acidic environments.

## 1 Introduction

Organisms are exposed to a variety of stress factors in their surroundings. Acid stress is a main stress factor in several bacterial species, and only a limited number of them evolved mechanisms to cope with high acidic environments. The bacteria that produce lactic acid as part of their natural metabolic processes are called Lactic Acid Bacteria (LAB) (Von Wright and Axelsson, 2019). LABs are Gram-positive, non-spore-forming, aerotolerant anaerobic (can thrive even in the presence oxygen) bacteria (Higuchi et al., 2000; Ayivi et al., 2020). LABs are found in diverse environments, where their number, taxonomic, and genomic diversity change depending on the characteristics of the environment (George et al., 2018).

LABs can be isolated from natural environments such as water and soil, however, their abundance in these environments has not been found at high levels (Chen et al., 2005). LABs are also found on host organisms mostly associated with digestive and excretory tissues (Pessione, 2012). Diet and health status determines the number and diversity of LABs found on host organisms (Hove et al., 1999). Fermentation is a centuries-old process that is widely used in the production of various foods by human societies all over the world (Mishra et al., 2017). LABs create a high acidic environment during fermentation and outcompete spoilage-causing microorganisms (Lamont et al., 2017). Moreover, fermented foods have an important place in human diet because the fermentation process plays a significant role in preserving the food taste, quality, and enrichment of the food content (Sharma et al., 2020). The utilization of lactic acid bacteria in food products, which may have positive health benefits on host, has become extremely popular in recent times (Nuraida, 2015). Therefore, natural fermentation environments, and fermented products are the niches where highest diversity and number of LABs are found (van Hylckama Vlieg and Hugenholtz, 2007; Ouoba et al., 2010; Phan et al., 2017).

In LABs, lactic acid is generated through several pathways of carbohydrate metabolism, mainly by the glycolysis pathway (Sheeladevi and Ramanathan, 2011). Lactic acid is released into the environment during the fermentation in a nutrient-rich environment, and this process causes acidification of the extracellular environment (Kajfasz and Quivey, 2011). Due to charge difference between the extracellular and intracellular environments, lactic acid in the acidified extracellular environment eventually passes into the cytoplasm by simple diffusion (Serrazanetti et al., 2009). Acidification of the intracellular environment has effects that may result in bacterial mortality such as disrupting the integrity of protein structures, and metabolic activities (Wu et al., 2014). LABs evolved acid resistance pathways to cope with this high acid stress environment. In general, decarboxylation metabolic pathways are used by LABs, and glutamate decarboxylase (GAD) pathway (GDP) is a major acid resistance mechanism that enable LABs’ survival in low pH environments (Yang et al., 2021).

The bacterial glutamate-dependent GAD system transports extracellular glutamate into the cell and converts it to gamma-aminobutyric acid (GABA). During this process the protons present in the intracellular environment participate in the reaction. Thus, the bacteria can regulate the decreasing pH level (Cotter and Hill, 2003). Since the GABA molecule, a byproduct of glutamate decarboxylation, is a very stable molecule, it does not re-ionize and does not alter the ion balance in the cell. Then, the GABA molecule produced in the cell can be transported out of the cell by the antiporter of the GAD system, or it can remain in the cell (Karatzas et al., 2010). Because the GAD system allows bacteria to successfully adapt to low acid conditions, different bacterial species from distinct habitats contain GDP genes in their genome. The structure and genomic organization of the GAD system, on the other hand, may change between species and even strains. Some species, for instance, have more than one decarboxylase (*gad*) and antiporter (*gadC*) gene, while others may not have the antiporter gene together with the enzyme gene (Cotter et al., 2001). The GAD system is mostly investigated in *Lactobacillus* (Yunes et al., 2016), *Lactococcus* (Redruello et al., 2021), and *Streptococcus* (Yang et al., 2008) LAB genera which show variable operon structure (tandem or nearby together presence of enzyme and transporter genes). In species such as *L. brevis*, *L. reuteri*, *L. buchneri*, and *L. lactis*, the antiporter gene creates an operon structure with the GAD gene, while in species such as *L. plantarum*, *L. fermentum*, there is only a single GAD gene, and there may not be a specific glutamate/GABA antiporter (*gadC*) gene (Wu et al., 2017). Among the many examined LAB genera, the *Lactobacillus* genus has the most significant GABA production potential with species such as *L. brevis* (Wang et al., 2018), *L. plantarum* (Di Cagno et al., 2010), *L. buchneri* (Park and Oh, 2006), and *L. paracasei* (Komatsuzaki et al., 2008) reported as high GABA producers.

*L. brevis* is the best-examined species in the genus and has a high GABA-producing capability. Only *L. brevis* possesses two GAD genes that produce isozyme GADs among the LABs examined so far (Wu and Shah, 2017; Lyu et al., 2018). Moreover, *L. brevis* genome has a GAD operon consisting of *gadR* involved in regulation of the system, *gadC* synthesizing the glutamate/GABA antiporter, and *gadA* that codes for GAD. Since no promoter or terminator structure could be found between *gadA* and *gadC* genes (Lyu et al., 2018), it is suggested that these two genes are transcribed together in a synchronized manner by forming an operon structure. The *gltX* (glutamate-tRNA ligase) gene, positioned downstream of the *gadA* gene, is also considered a part of the GAD cluster structure (Cui et al., 2020). The second *L. brevis* GAD gene, *gadB*, is found in a different region of the genome far away from the GAD operon without any operon formation (Lyu et al., 2018). One needs to be careful about the discrepancy in literature for the nomenclature of *L. brevis* GAD genes (Cui et al., 2020). Some articles use the *gadA* nomenclature for the GAD gene in the operon structure, whereas others use *gadB* (Wu et al., 2017; Lyu et al., 2018). Moreover, for LABs without a GAD operon structure, the GAD gene is also called *gadB*. Rather different compared to *L. brevis*, the other well-studied LABs, *L. plantarum*, has only one GAD coding enzyme in its genome, the *gadB*. There is no *gadR*, *gadC* (glutamate/GABA antiporter) or any GAD operon structure in *L. plantarum* genome (Wu et al., 2017). Many studies showed that *L. plantarum* can produce appreciable amounts of GABA, so there must be a transporter responsible for transporting glutamate and GABA in and out of the cell. A glutamate/gamma-aminobutyrate transporter family protein coded by the *yjeM* gene can be the best candidate for such a transporter (Valenzuela et al., 2019), however, there are no functional or *in silico* studies characterizing this gene/protein.

We aimed to study the molecular evolution and population genetics of GDP in LABs to understand evolutionary processes shaping adaptation to acidic environments comparing species where the GDP genes are organized in an operon structure (*L. brevis*) versus a species that lacks an operon structure (*L. plantarum*). First, we performed molecular population genetics and molecular evolution analyses with *L. brevis* and *L. plantarum* strains with whole genome data isolated from diverse food and non-food environments to compare the natural selective forces shaping the GDP genes in LABs with and without an operon structure. Second, the GAD system gene organization in diverse LAB species is compared *via* phylogenetic analyses. Based on phylogenetic relationships, divergence patterns of *L. brevis* and *L. plantarum* GAD system genes are compared with respect to other LAB species. Finally, the functional consequences of amino acid changes observed in *L. brevis* and *L. plantarum* genes is investigated with molecular structural analyses.

## 2 Materials and methods

### 2.1 Data collection and annotation of GDP genes in LABs

All gene sequences and genome data used are downloaded from NCBI (http://www.ncbi.nlm.nih.gov/Entrez/) web site (last accessed on 15/08/2022). Only strains having the whole genome or contig data containing all of the GAD pathway genes, and with clearly indicated isolation sources were selected from the results of the NCBI search. GeneMark.hmm prokaryotic web tool (http://exon.gatech.edu/GeneMark/gmhmmp.cgi) was used to identify and distinguish the GAD operon genes. Incomplete and pseudogene sequences were excluded from the analyses. Based on reported isolation sources, 30 strains from five different isolation groups (populations) were formed for *L. brevis* and 88 strains from nine isolation groups were formed for *L. plantarum*. One of the groups created for both bacterial populations is the feces group, which is a non-food source. All other groups contain strains isolated from various food environments. Supplementary Table S1 shows the isolation sources, NCBI accession numbers, and group assignment of all *L. brevis* and *L. plantarum* strains used for analyses. Due to discrepancy for the nomenclature of *L. brevis* GAD genes (Wu et al., 2017; Lyu et al., 2018; Cui et al., 2020), to avoid confusion, we used *gad1* and *gad2* for the GAD genes inside and outside the *L. brevis* operon, respectively.

Since there is no report of a specific *gadC* gene in the *L. plantarum* genome, the amino acid sequence of *L. brevis gadC* is searched *via* BLAST (https://blast.ncbi.nlm.nih.gov/Blast.cgi) against all available *L. plantarum* genomes. The glutamate/gamma-aminobutyrate transporter family protein coding gene *yjeM* was identified as the homolog of *gadC* gene. Similarly, *gadC* and *yjeM* homolog transporter genes of other LABs were identified by amino acid and nucleic acid BLAST searches against their respective genomes. Supplementary Tables S2, S3 show the GAD system genes’ information for 32 bacterial species. Additionally, the 16S rRNA sequence data of 32 bacterial species taken from NCBI are shown in Supplementary Table S4.

### 2.2 Sequence alignment and phylogenetic analyses

UGENE (Okonechnikov et al., 2012) and MEGA-X (Kumar et al., 2018) were used to visualize and align nucleic acid and amino acid sequences. Alignments of all GAD pathway genes and 16S rRNA from *L. brevis* and *L. plantarum* strains, and 32 bacterial species were performed by the MUSCLE alignment algorithm (Edgar, 2004). To obtain a more uniform and optimal alignment, homologous nucleotide sequences were first converted into amino acid sequences in MEGA-X, and aligned with MUSCLE. Then, aligned amino acid sequences were converted to nucleic acid sequences.

Phylogenetic analyses were performed by MEGA-X. The most suitable phylogenetic tree models for substitutions (nucleotide or amino acid) were predicted with MEGA-X/Models by using sequence alignments. The model with the lowest Bayesian Information Criterion (BIC) score was chosen, and the Maximum Likelihood (ML) method was applied. In addition, the 1000 bootstrap method was employed to evaluate the phylogeny, and the complete deletion option in MEGA-X was used to treat the sequence alignments. 16S rRNA sequences were used for reconstructing the phylogenetic relationships of bacterial species. Phylogenetic relationships of bacteria based on each individual GAD pathway gene was also evaluated.

### 2.3 Molecular population genetic analyses

#### 2.3.1 Intraspecies analyses

Within population genetic summary statistics of *L. brevis* and *L. plantarum* populations for nucleotide diversity included segregating sites (S), total number of mutations (Eta), pairwise comparison of nucleotide diversity (Pi, *π*) (Nei, 1987), and average number of nucleotide differences between sequences estimated by Watterson theta (ThetaW, *θ*) (Watterson, 1975; Nei, 1987). Allele frequency spectrum based selection tests included Tajima’s *D* (Tajima, 1989), Fu and Li’s D* and *D* (Fu and Li, 1993), Fu and Li’s F* and *F* (Fu and Li, 1993), Normalized Fay and Wu’s *H*_*n*_ (Fay and Wu, 2000; Zeng et al., 2006), Zeng *E* (Zeng et al., 2006), and Achaz *Y* (Achaz, 2008) test statistics. Population genetic parameters were estimated by DnaSP 6 (Rozas et al., 2017).

#### 2.3.2 Interspecies analyses

Adaptive protein evolution parameter estimates using different LABs’ GAD pathway gene sequences included McDonald and Kreitman test (MKT) (McDonald and Kreitman, 1991), Neutrality index (NI) (Rand and Kann, 1996), Alpha value (proportion of adaptive substitutions) (Smith and Eyre-Walker, 2002), and Direction of selection (DoS) (Stoletzki and Eyre-Walker, 2011). These parameters were estimated using DnaSP version 6 (Rozas et al., 2017).

### 2.4 Codon usage bias

Codon usage bias analyses included codon bias index (CBI) (Bennetzen and Hall, 1982) and codon adaptation index (CAI) (Sharp and Li, 1987) parameter estimates. CBI was used to investigate whether there is preference (bias) for certain synonymous codons. DnaSP v6 was used for the CBI estimates. Effect of gene expression on codon usage preference was estimated by CAI. CAIcal (http://genomes.urv.es/CAIcal/), a web-based tool, was used to calculate the CAI values. The codon usage tables required for CAI estimates were retrieved from http://www.kazusa.or.jp/codon/.

### 2.5 Protein sequence based analyses

The biochemical properties of the amino acid changes generated by non-synonymous polymorphisms were characterized based on the information from NCBI (www.ncbi.nlm.nih.gov/Class/Structure/) and literature. To investigate the impact of replacement changes on the protein isoelectric points (pI) Prot Pi (www.protpi.ch), a web-based tool, was used. The pKa values used in the pI calculation were estimated by ExPASy (https://www.expasy.org/). The effect of amino acid change on the protein function (i.e., deleterious vs. neutral) was estimated by PROVEAN (http://provean.jcvi.org/) (Choi and Chan, 2015). Selection on amino acid residues was tested by maximum likelihood site model analyses using the PAML package (codeml) (Yang, 2007) and its graphical user interface PAMLX (Xu and Yang, 2013).

### 2.6 Protein structure prediction

Several protein structure prediction servers were employed to investigate the structure of GDP proteins as there was very limited structure information on GDP proteins in literature. I-TASSER (Zhang, 2008) was used to predict the GDP proteins’ secondary structures. The three-dimensional structures of GDP proteins were predicted using the AlphaFold v2 Colab server (AlphaFold2. ipynb) (Jumper et al., 2021). Phyre2 (Kelley et al., 2015) was mainly used for the transmembrane helix (TM-helix) prediction of transporter proteins. The protein structure predictions were performed using the wild type amino acid sequences (sequences without amino acid changes) of GDP proteins. PyMOL (DeLano, 2002) molecular visualization tool was used to visualize and color the predicted protein structures.

### 2.7 Statistical analyses

Distribution of synonymous and non-synonymous changes among different protein domains is compared by Chi-square tests. For continuous variables, deviations from normal distribution is tested by Shapiro test. Population genetic parameter estimates per gene were compared among populations by non-parametric Kruskal-Wallis one-way ANOVA followed by non-parametric Wilcoxon pairwise tests. For two group comparisons non-parametric Wilcoxon test is conducted. ggplot2 package (Wickham et al., 2016) was used for statistical graphing. All statistical analyses were conducted in R (https://www.r-project.org/).

## 3 Results

### 3.1 Population genetic analyses of GAD pathway genes within *L. brevis* and *L. plantarum* populations

**FIGURE 1:**
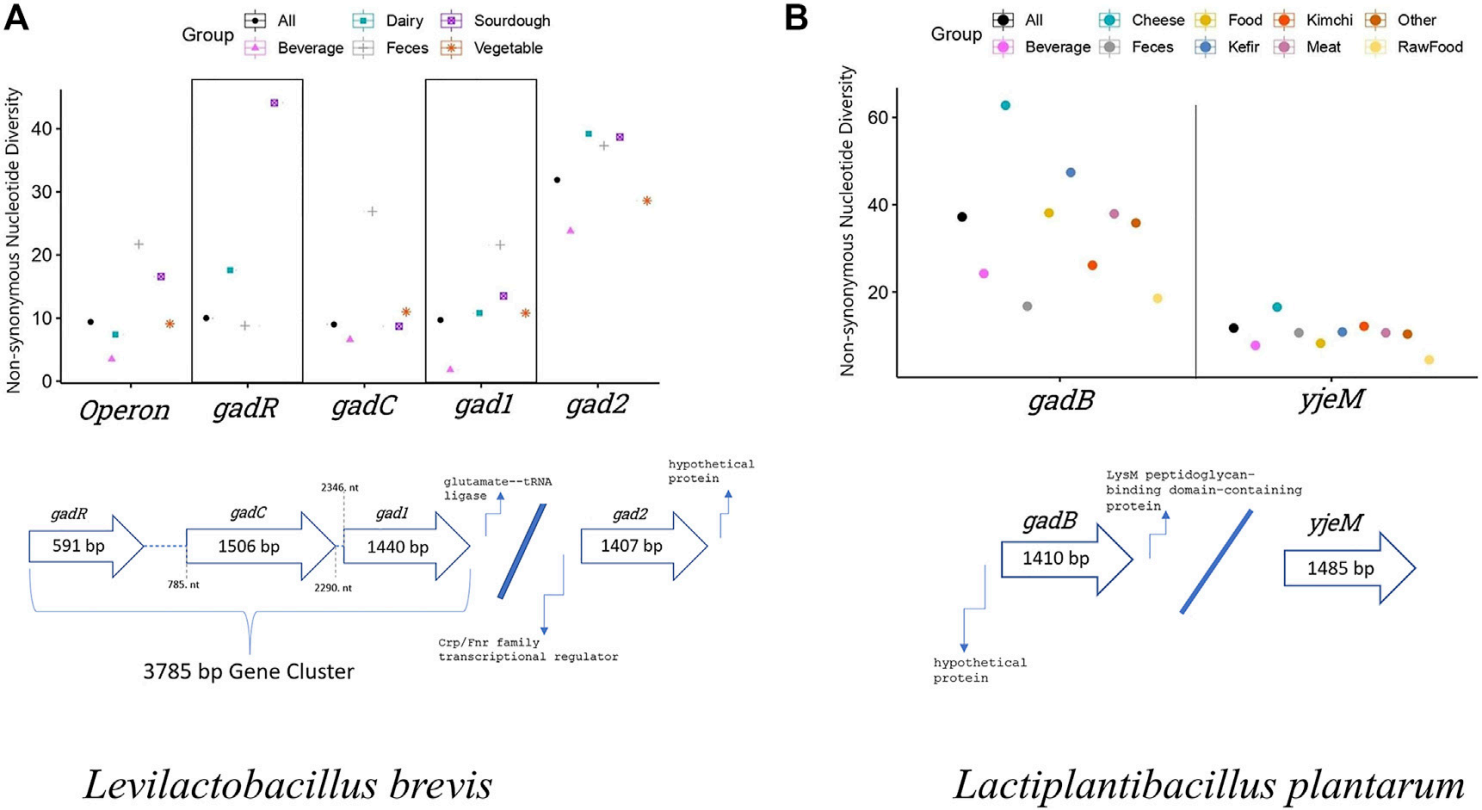
Non-synonymous nucleotide diversity estimates of GAD pathway genes from **(A)** *L. brevis* (*n* = 30), and **(B)** *L. plantarum* (*n* = 88) considering all samples, and indivudual populations isolated from different environments. Detailed sample size and other population genetic parameter estimates for *L. brevis* and *L. plantarum* populations are presented in Supplementary Tables S5, S6, respectively. Thick arrows show the GAD pathway gene organiation in *L. brevis* and *L. plantarum* genomes. Numbers inside the thick arrows show the length of each gene in base pairs (bp). Numbers outside the arrows indicate the relative nucleotide number (nt) positions of genes with respect to each other. *gadR*, Transcription regulator gene; *gadC* and *yjeM*, glutamate/GABA transporter gene; *gad1*, *gad2*, and *gadB*, glutamate decarboxylase enzyme gene. *L. brevis* operon includes *gadR*, *gadC*, and *gad1*.

In *L. brevis* GAD pathway consists of transcription regulator (*gadR*), glutamate/GABA transporter (*gadC*), and glutamate decarboxylase enzyme (*gad1* and *gad2*) genes. The operon is formed by *gadR*, *gadC*, and *gad1*. In total, sequence data of 30 strains from five isolation environments (populations) were analyzed. The transporter gene, *gadC*, showed the highest number of overall (synonymous and replacement/non-synonymous sites combined) segregating sites and mutations based on all (*n* = 30) *L. brevis* samples. *gadC* also had the highest overall nucleotide diversity based on segregating sites (*θ*), and average pairwise nucleotide difference (*π*) estimates (Table 1). The synonymous site nucleotide diversity was also highest in *gadC*, even higher than operon’s (*gadR*, *gadC*, and *gad1* combined) estimate. However, the glutamate decarboxylase enzyme coding gene, *gad2*, outside the operon showed significantly higher number of replacement/non-synonymous changes and non-synonymous site diversity compared to other genes and operon (non-parametric ANOVA *p* = 0.02; Figure 1; Table 1). Within each *L. brevis* population (such as different fermented foods, feces, etc.) *gad2* again showed the highest number of replacement changes and non-synonymous site diversity (Figure 1; Supplementary Table S5; Supplementary Figure S1). Interestingly, *gadC* was the most conserved GAD pathway gene with lowest non-synonymous site diversity in nearly all populations except the feces strains (Figure 1; Supplementary Table S5). Feces strains showed the highest overall, synonymous, and replacement polymorphism nucleotide diversity, followed by sourdough strains (Figure 1; Supplementary Table S5; Supplementary Figure S1). Phylogenetic analyses with gene sequences separated *gad1* and *gad2* into two distinct clades (Supplementary Figure S2). For all four genes, sequences from different isolation sources clustered together with no apparent separation of populations into distinct clades suggesting sharing of variants/alleles in multiple populations (Supplementary Figure S2).

**TABLE 1:** Nucleotide diversity population genetic summary statistics for glutamate decarboxylase pathway genes in the examined *L. brevis* and *L. plantarum* populations.

		Total bases	Syn. sites	Nonsyn. Sites	S (Eta)	Sing	Par	Syn. Pol	Rep. Pol	Theta-W (θ) all sites	π all sites	π Syn. sites	π Nonsyn. Sites
*L. brevis* (N = 30)													
	Operon	3516	807.32	2708.68	214 (217)	104	110	175	27	143.2	113.3	485.1	9.4
	*gadR*	591	135.36	455.64	28 (28)	9	19	23	5	119.6	108.4	452.9	10
	*gadC*	1492	346.71	1141.29	112 (114)	65	47	101	12	189.5	131.8	561.2	9
	*gad1*	1440	325.26	1111.75	60 (61)	20	40	51	10	105.2	101.3	427.4	9.7
	*gad2*	1401	321.24	1076.76	76 (77)	32	44	59	18	136.9	125	451.7	31.9
*L. plantarum* (N = 88)													
	*gadB*	1410	323.35	1077.65	121	39	82	82	38	170.7	86.2	247.4	37.2
	*yjeM*	1485	351.76	1130.24	65	30	35	51	14	86.7	50.6	178.2	11.7

N, Number of sequences analyzed; Syn, Synonymous sites, Nonsyn, Non-synonymous sites; S, number of segregating sites; Eta, Number of mutations; Sing, Singleton variable sites; Par., Parsimony informative sites; Syn. Pol., number of synonymous polymorphisms; Rep. Pol., Number of replacement (non-synonymous) polymorphisms. θ (theta) and π (pi) values represent percent sequence diversity and for exact estimates table values should be multiplied by 10^−4^. Jukes-Cantor correction applied estimates are presented for π estimates. Operon includes *gadR*, *gadC*, and *gad1* combined and total base count for operon includes only the coding regions.

In *L. plantarum*, glutamate/GABA transporter (*yjeM*) and glutamate decarboxylase enzyme (*gadB*) genes form the GAD pathway. In total, sequence data of 88 strains from nine isolation environments (populations) were analyzed. *gadB* had higher overall, synonymous, and replacement polymorphism nucleotide diversity compared to *yjeM* (Table 1). In all populations *gadB* had higher number of replacement polymorphisms and non-synonymous nucleotide diversity (non-parametric Wilcoxon *p* = 0.0002), and *yjeM* was the most conserved gene (Figure 1; Supplementary Table S6; Supplementary Figure S1). Cheese strains showed the highest overall, synonymous, and replacement polymorphism nucleotide diversity, followed by kefir strains (Figure 1; Supplementary Table S6; Supplementary Figure S1). Phylogenetic analyses with both genes showed that sequences from different isolation sources clustered together with no apparent separation of populations into distinct clades suggesting sharing of variants/alleles in multiple populations (Supplementary Figure S3).

#### 3.1.1 Identifying nature of selection driving nucleotide diversity of GAD pathway genes within *L. brevis* and *L. plantarum* populations

Following within population nucleotide polymorphism analyses, several neutrality tests were conducted for all GDP genes in both species. Tajima and Fu-Li tests are allele frequency spectrum tests that compare the two different nucleotide diversity estimates *θ* and *π*. Negative test results indicate negative selection, recent selective sweep or exponential population growth, whereas positive test results generated under balancing selection, positive selection or sudden population contraction. When all samples were analyzed together, excess of rare or singleton changes was evident in all genes for both species based on negative Tajima’s *D* (TD), Fu-Li’s D*, and Fu-Li’s F* test results (Table 2). Excess of rare/singleton replacement variants was more evident in *L. brevis* samples, whereas excess of both synonymous and replacement changes was observed in *L. plantarum* samples (Table 2). When neutrality tests were conducted within each population separately, still negative test results were observed for nearly all genes in all populations for both species (Supplementary Tables S7, S8). Negative selection, recent selective sweep acting on the genes or exponential population growth can lead to observed negative Tajima’s and Fu-Li test results. Less negative test results observed within each separate population suggest historical negative selection eliminating mutations happening on these genes rather than a recent selective sweep or fast population growth occurring independently in each population. To further test the negative selection hypothesis, other allele frequency spectrum based tests that use out group species were conducted (Supplementary Table S9). The advantage of using outgroup species is that the nucleotide changes observed can be differentiated as ancestral or derived. Analyses with outgroup homologous gene sequences suggested that most nucleotide changes observed are derived, and negative selection is keeping these changes at low allele frequency.

**TABLE 2:** Neutrality tests summary statistics for glutamate decarboxylase pathway genes in the examined *L. brevis* and *L. plantarum* populations.

	TD	TD Cod	TD Syn	TD Nonsyn	TD Silent	Fu-Li’s D*	Fu-Li’s F*
*L. brevis* (N = 30)							
*Operon*	−0.89	−0.84	−0.60	−2.24**	−0.67	−1.74	−1.72
*gadR*	−0.37	−0.37	0.06	−1.75^#^	0.06	−0.40	−0.45
*gadC*	−1.26	−1.27	−1.11	−2.16*	−1.11	−2.40^#^	−2.39^#^
*gad1*	−0.23	−0.23	0.15	−1.81*	0.15	−0.59	−0.56
*gad2*	−0.42	−0.42	−0.25	−0.85	−0.25	−1.21	−1.12
*L. plantarum* (N = 88)							
*gadB*	−1.68^#^	−1.71^#^	−1.75^#^	−1.48	−1.70^#^	−1.46	−1.86
*yjeM*	−1.37	−1.37	−1.26	−1.46	−1.26	−2.91*	−2.74*

TD, Tajima’s *D* test; Cod., Coding sites; Syn, Synonymous sites. Nonsyn, Non-synonymous sites; Silent, Silent sites; N, Sample size. Operon includes *gadR*, *gadC*, and *gad1* combined. #Represent 0.10 > *p* > 0.05; * represent *p* < 0.05, ** represent *p* < 0.01. ***Represent *p* < 0.001.

We also used maximum likelihood score based site model analyses to identify sites that can be under positive selection with the PAML package (Yang, 2007). No positive selection on any sites on any of the genes was identified.

Three-dimensional molecular structure (PDB code 5GP4) of *L. brevis* GAD protein (coded by *gad2*) and its functional domain information is available (Huang et al., 2018). The GAD protein is divided into N-terminal, PLP-binding (includes all active and co-factor binding residues), and Small (C-terminal) domains (Supplementary Table S10A; Supplementary Figure S4). *L. brevis gad1*, *gad2*, and *L. plantarum gadB* amino acid sequences are aligned, and nucleotide positions coding for each domain are identified for all three genes (Supplementary Table S10A; Supplementary Figure S4). Then, overall and replacement nucleotide diversity estimates, and Tajima’s *D* tests are conducted on each domain separately. Although overall nucleotide diversities were higher in the PLP-binding domains compared to other domains for all GAD genes, replacement nucleotide diversities were lower in the PLP-binding domains, suggesting higher conservation on PLP-binding domain coding sequences (Supplementary Table S10B). Replacement site Tajima’s *D* tests were negative for PLP-binding domain sites in *gad1*, *gad2*, and *gadB* suggesting negative selection against amino acid changing mutations especially in this domain (Supplementary Table S10B). Similar domain specific analyses cannot be performed for *gadR*, *gadC*, and *yjeM* genes due to lack of well-defined domain information.

### 3.2 Distribution and biochemical characteristics of amino acid changes observed on GAD pathway proteins

Following nucleotide based population genetics analyses, the distribution and nature of replacement changes observed on all GAD pathway genes are investigated. In these analyses independent occurrence of the same amino acid (aa) position change in different populations is summed to determine the total number of aa changing mutations on the genes. Among all *L. brevis* strains a total of seven aa changes were observed in *gadR*. Eighty six percent (6/7) of the changes were singletons (observed only once), and 57% (4/7) of changes were observed in sourdough strains. Seventy one percent (5/7) of the aa changes were conservative (not leading to charge, class, polarity, etc. changes), and only one of the singleton aa changes that leads to charge, class (basic to sulfuric aa), polarity, and interaction mode change was identified as deleterious with PROVEAN analysis (Supplementary Table S11A). For the *gadC*, 16 replacement changes were observed, and 88% (14/16) of the changes were singletons. Fifty percent (8/16) of the changes were observed in the feces strains. Only four of the aa changes (all singletons) that results in class, polarity, and interaction mode changes were identified as deleterious changes (Supplementary Table S11B). Sixteen replacement changes were observed for *gad1*. Again feces strains showed more changes compared to other populations. Although most changes were observed only once, three of the aa changes were seen in more than one population, and G471H was observed in nearly all (4/5) isolation groups. Over 85% (14/16) of the aa changes were biochemically conserved changes and only one change is identified as deleterious (Supplementary Table S11C). With 41 replacement changes, *gad2* showed the highest number of aa changes, and unlike other genes the distribution of changes was even among different isolation groups, where nearly all aa changes were seen in more than one population. Moreover, the percentage of biochemically unconserved and deleterious changes was higher for *gad2* compared to other genes (Supplementary Table S11D).

The total number of replacement changes observed in *gadB* among all *L. plantarum* strains was higher than *L. brevis* GAD genes combined. Most replacement changes were non-singletons observed in more than one population. The number and percentage of biochemically unconserved and deleterious changes was also higher in *gadB* than *L. brevis* GAD genes combined. The unconserved and deleterious aa changes were observed in all *L. plantarum* populations (Supplementary Table S12A). The number of replacement changes in *L. plantarum yjeM* was much lower compared to *gadB*, and over 85% of the changes were biochemically conserved aa changes with no deleterious effects. Most aa changes were observed in at least two populations (Supplementary Table S12B).

### 3.3 The influence of amino acid changes on the charge distribution and structure of GAD pathway proteins

The effect of aa changes on the isoelectric point (pI) and charge distribution of GAD pathway proteins is investigated. For each gene/protein a sequence without any aa change is selected as a wild type, and its pI and charge at pH values ranging from 4.00 to 7.00 is estimated. Then, pI and charge at various pH value estimates for each gene’s protein sequences with different aa changes are calculated. Despite abundance of aa changes with different biochemical properties, the pI and charge estimates of all proteins with aa changes were nearly identical to each other and to their respective wild types (Supplementary Table S13) indicating minimal or no net effect of these aa changes on protein characteristics. Even for protein sequences with more than one aa change (such as GadC with seven aa changes, Gad2 with five aa changes, GadB with nine aa changes) the effect of the aa changes on estimated pI and charge was negligible (Supplementary Table S13). A close look at the biochemical properties of amino acids observed on proteins with multiple aa changes shows that they are mostly conserved aa changes (Supplementary Tables S11, S12).

**FIGURE 2:**
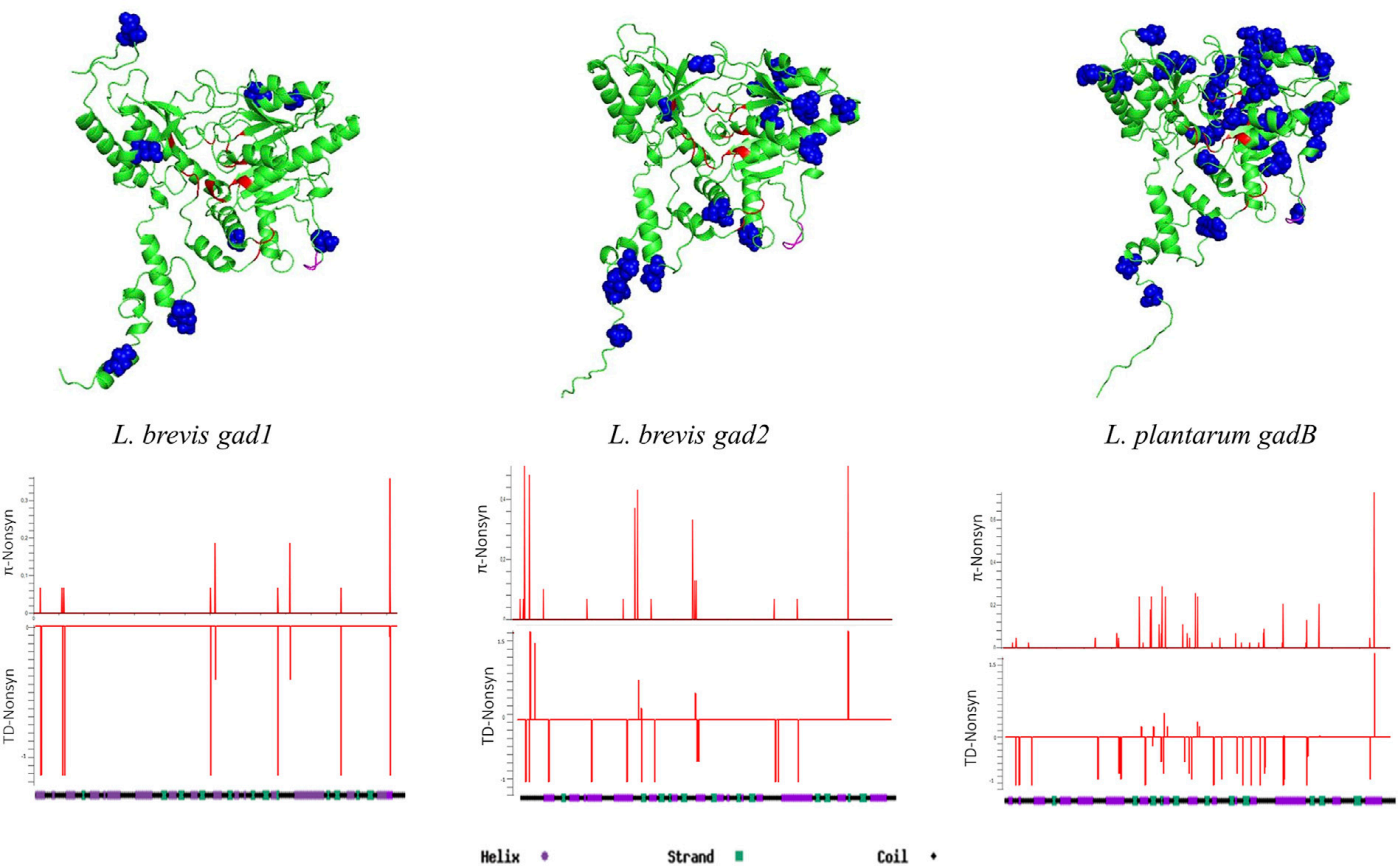
3D structure predictions for Gad1, Gad2, and GadB proteins. Blue colored residues represent replacement sites, red colored residues represent ligand and cofactor (PLP) binding sites, and pink colored residues represent the flexible loop region. Sliding-window analyses show non-synonymous nucleotide diversity (*π*) and Tajima’s D test results throughout the genes.

Except for *L. brevis* Gad2, there was no secondary or three-dimensional (3D) structure information for any of the GAD pathway proteins examined. I-TASSER was used for secondary structure predictions, Phyre2 was used for transmembrane helix structure predictions, and AlphaFold was used for 3D structure predictions. First, GAD enzyme protein structures were analyzed. There was 84% aa sequence similarity between Gad2 and GadB, and 52% similarity between Gad2 and Gad1 (Supplementary Figure S2). Despite differences in aa sequences, the secondary and 3D structures of all GAD enzymes were similar (Figure 2; Supplementary Figure S5) except the C-terminal regions. Gad2 and GadB C-terminal regions form a C-plug structure that curls towards the active site, whereas this structure is not observed for Gad1. Using aa sequence alignments and secondary structure information (Supplementary Figure S5) important residues, and PLP and L-glutamine binding sites were identified for Gad1, Gad2, and GadB (Supplementary Table S14). None of the observed replacement changes in *L. brevis* or *L. plantarum* GAD genes (detailed above in Section 3.2) led to aa change in these important residues (Figure 2). Sliding window analyses with window size 10 base pairs (bp) and step size 1 bp was conducted over entire GAD gene sequences calculating high resolution replacement polymorphism diversity (*π* non-synonymous) and non-synonymous site Tajima’s *D* values. Whereas higher non-synonymous site diversity driven by rare/singleton replacement changes (negative Tajima’s *D* values) on the second half of Gad1 was observed, higher non-synonymous site diversity driven by both rare and common (positive Tajima’s *D* values) replacement changes on the first half of Gad2 was observed (Figure 2). For GadB, nearly all replacement changes were on the PLP-binding domain (Figure 2).

**FIGURE 3:**
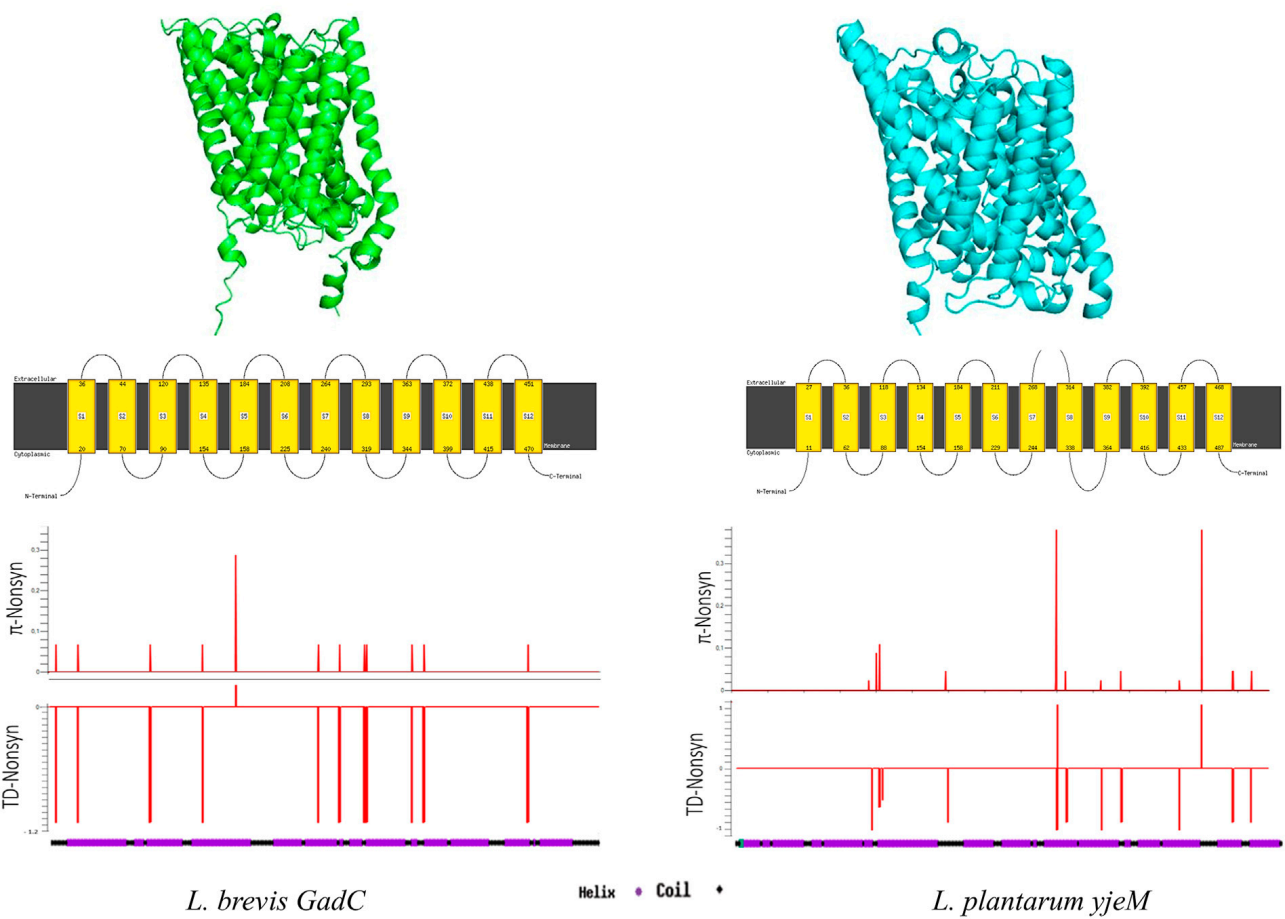
Secondary and 3D structure predictions for GadC, and YjeM proteins. Sliding-window analyses show non-synonymous nucleotide diversity (*π*) and Tajima’s D test results throughout the genes.

Structure prediction programs identified the *E. coli* glutamate/GABA antiporter (PDB code 4DJI) (Ma et al., 2012) as the best candidate for structural analyses of glutamate/GABA transporters GadC and YjeM. Although the aa sequence similarity between GadC and YjeM is only around 18% (Supplementary Figure S6), the secondary and 3D structures of both transporter proteins were very similar, where 12 transmembrane helixes formed by the long alpha-helixes and short coils between them were predicted (Figure 3; Supplementary Figure S7). Sliding window analyses showed that non-synonymous site diversity driven by rare/singleton replacement changes were distributed throughout the GadC, however, the replacement changes were more evident in the second half of YjeM (Figure 3).

There is very limited structure information on GAD system regulatory proteins, and potential regulatory proteins have not been annotated in most LABs. Structure analyses identified a simple 3D structure consisting of alpha-helices with a possible single TM-helix (Supplementary Figure S8). The small number of rare/singleton replacement changes were observed in a narrow window between residues 50 and 100 outside the TM-helix region (Supplementary Figure S8).

We also examined the distributions of singleton vs. non-singleton, and deleterious vs. non-deleterious replacement changes on the alpha-helix, beta-sheet, and coil regions for all GDP genes. None of the results was significant suggesting negative selection against replacement changes is evident for all secondary structures.

### 3.4 Codon usage bias

Most amino acids are coded by more than one codon (codon redundancy), and codon preference can be under selection, leading to non-random codon usage bias. Selection on codon usage is generally observed in rapidly growing bacterial populations, and genes with high expression levels (Fox and Erill, 2010). One of the measures, the codon bias index (CBI), measures the ratio between preferred codons and the total number of codons in a gene, and its value ranges between −1 and 1. A zero CBI value suggests random codon choice, whereas 1 and −1 indicates preferred and unpreferred codon utilization, respectively (Bahiri-Elitzur and Tuller, 2021). Codon adaptation index (CAI) measures the frequency of codon usage for a gene utilizing the predetermined reference values. CAI is generally used as an indication of expression level of a gene. CAI values range between zero and one, where values close to one indicate higher expression levels (Bahiri-Elitzur and Tuller, 2021). The CAI estimates for all GAD pathway genes in *L. brevis* and *L. plantarum* were similar, and greater than 0.77 (range 0.77–0.83) indicating high expression of GAD pathway genes (Supplementary Table S15) in both species. Moreover, the CBI values were similar and greater than 0 (range 0.29–0.45) suggesting moderate codon usage bias (Supplementary Table S15).

### 3.5 Examining nature of selection driving molecular evolution of GAD pathway genes with interspecies analyses

Following molecular population genetic analyses within *L. brevis* and *L. plantarum* populations, molecular evolution and divergence patterns of *L. brevis* (with operon structure) and *L. plantarum* (without operon structure) GAD system genes are compared with respect to other LAB species. These interspecies analyses are conducted using 32 bacterial species (31 LABs and the outgroup *E. coli*) that are commonly found in acidic fermented food environments. These species are chosen so that each one has at least one high coverage whole genome sequence with annotated GAD pathway genes, and they represent species with and without GAD operon structure.

For each gene we performed McDonald-Kreitman (MK) tests, comparing the ratio of non-synonymous to synonymous variation within *L. brevis* and *L. plantarum* samples to the ratio of non-synonymous to synonymous divergence between *L. brevis* and *L. plantarum*, and other LAB species. For most comparisons, between species divergence (non-synonymous/synonymous fixed difference ratio) was greater than within species non-synonymous/synonymous polymorphism ratio, indicated by statistically significant MK test results, suggesting positive selection (adaptive protein evolution) on the genes (Supplementary Table S16). We also calculated proportion of adaptive substitutions in a MK test (alpha value), ratio of ratios in a MK 2 × 2 table (Neutrality index: NI), and difference between proportion of non-synonymous divergence and non-synonymous polymorphism (DoS: Direction of selection) values. Alpha and DoS values greater than zero, and NI values less than one indicate positive selection. For most tests, alpha and DoS estimates were greater than zero, and NI values were less than one (Supplementary Table S16) further supporting positive selection driving adaptive protein evolution in GAD pathway genes.

**FIGURE 4:**
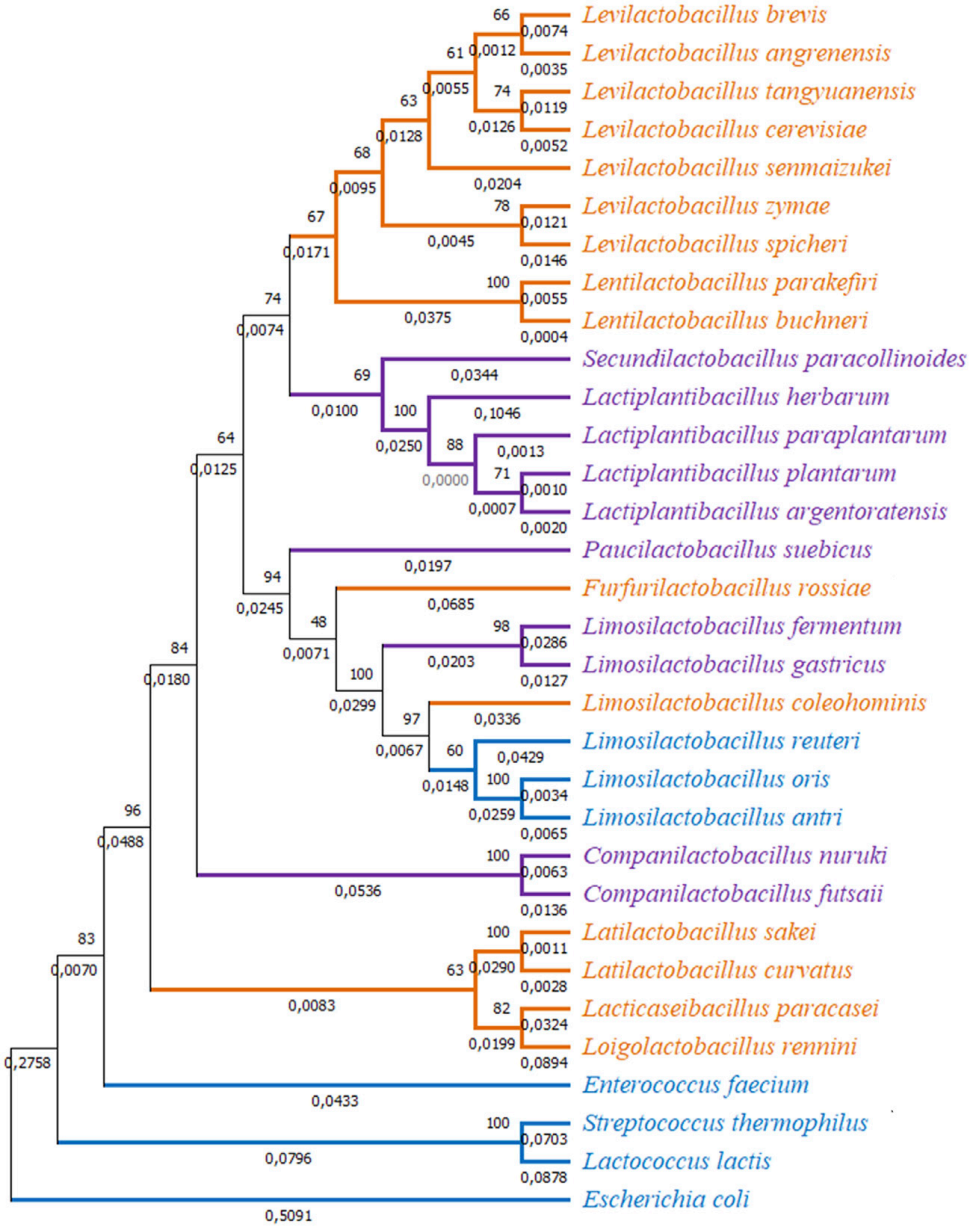
Phylogenetic tree based on 16S rRNA sequences from 32 bacterial species. The tree was constructed with MEGA-X using the Maximum Likelihood method and the Kimura 2-parameter model. One thousand replicates were used to generate the bootstrap consensus tree. A discrete Gamma (G) distribution was used for evolutionary rate variations across sites. Numbers on the nodes show the bootstrap support, numbers on the branches show substitutions per site. Orange color represents species where GAD pathway genes are organized in an operon structure. Purple color represents species without an operon structure, and blue color represents species with a small operon structure consisting of only a transporter and a GAD gene.

**FIGURE 5:**
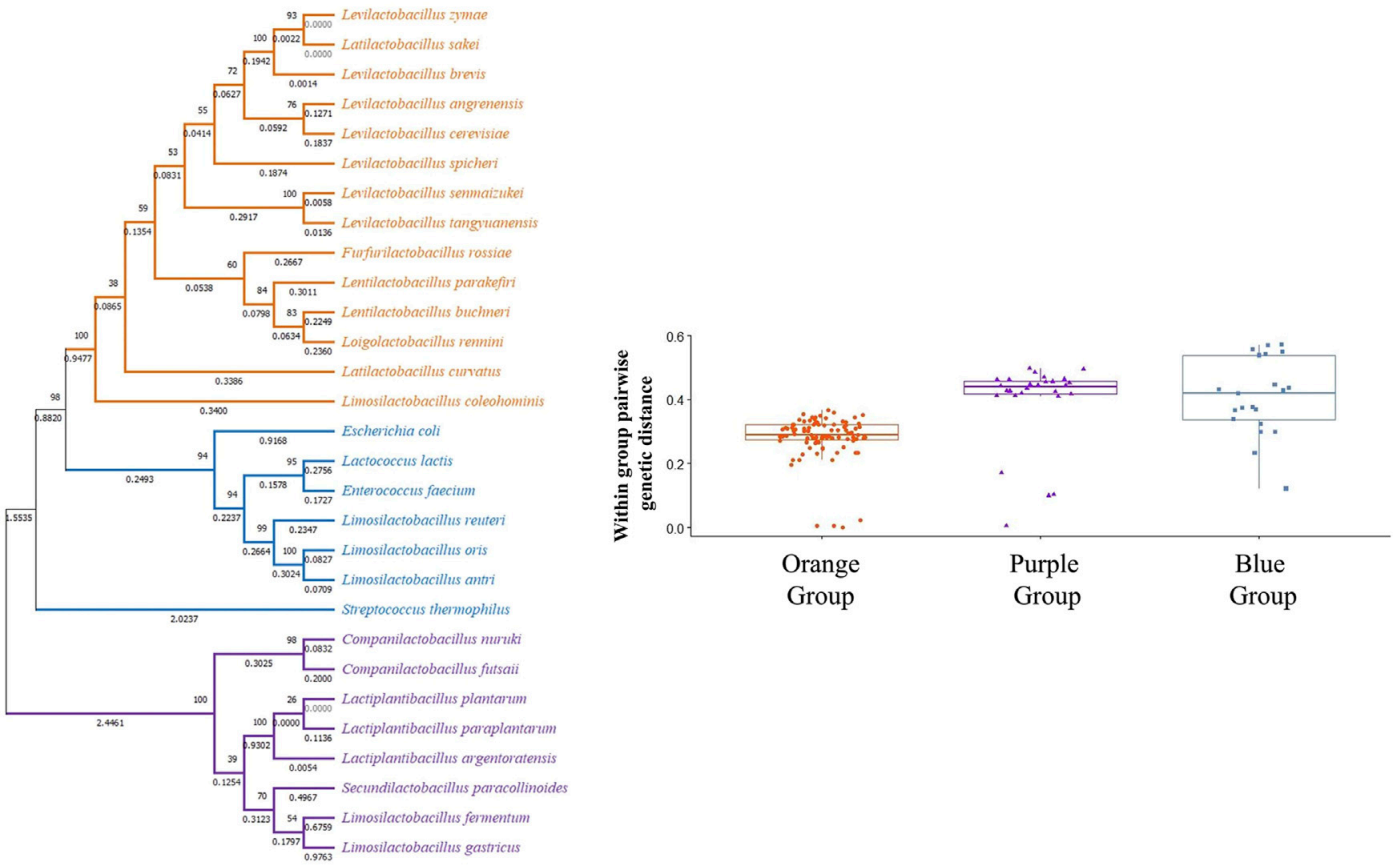
Phylogenetic tree based on transporter (gadC and yjeM) gene sequences from 29 bacterial species. The tree was constructed with MEGA-X using the Maximum Likelihood method and General Time Reversible (GTR) model. One thousand replicates were used to generate the bootstrap consensus tree. A discrete Gamma (G) distribution was used for evolutionary rate variations across sites. Numbers on the nodes show the bootstrap support, numbers on the branches show substitutions per site. Orange color represents species where GAD pathway genes are organized in an operon structure. Purple color represents species without an operon structure, and blue color represents species with a small operon structure consisting of only a transporter and a GAD gene.

**FIGURE 6:**
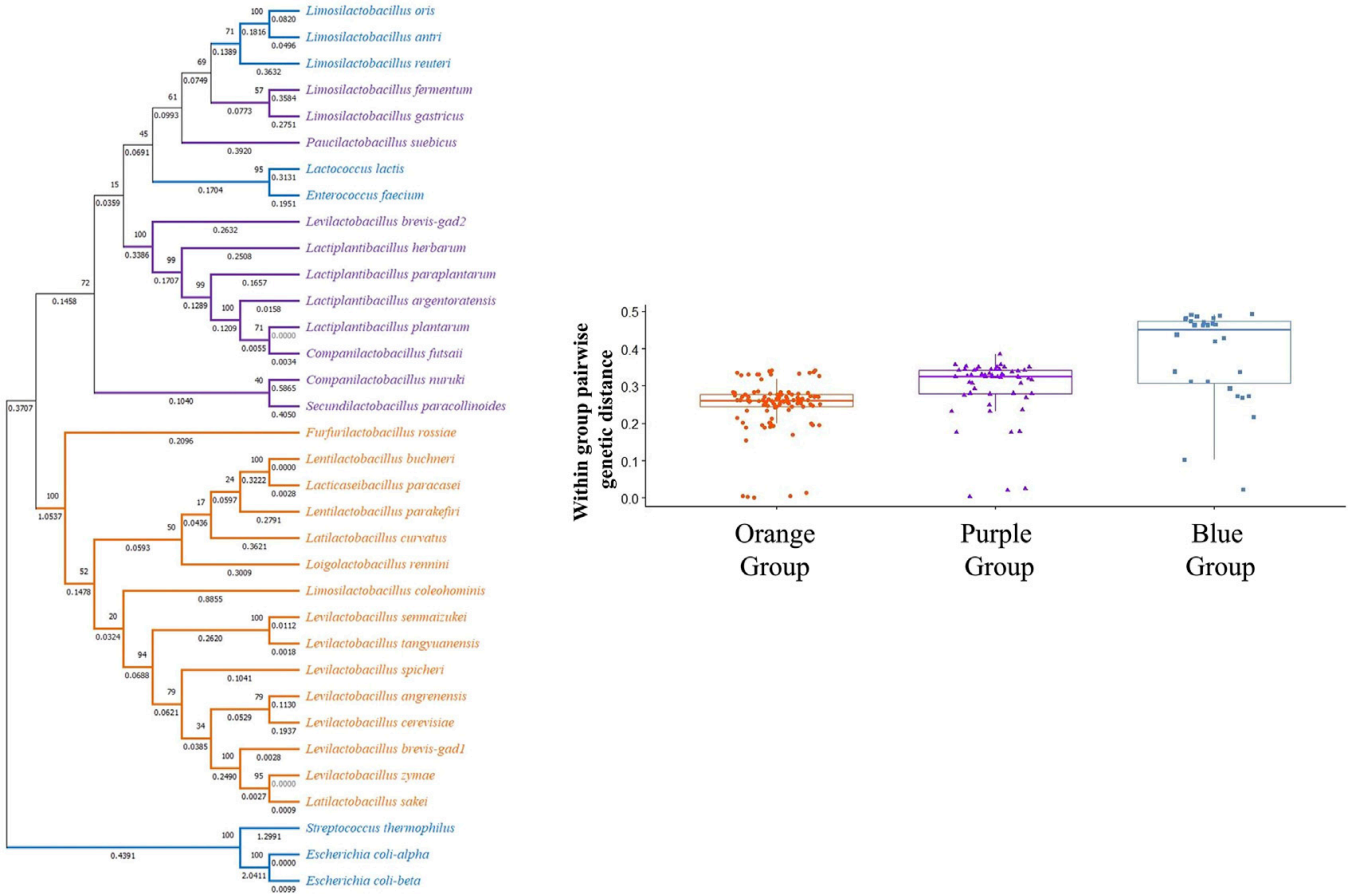
Phylogenetic tree based on gad gene sequences from 32 bacterial species. The tree was constructed with MEGA-X using the Maximum Likelihood method and General Time Reversible (GTR) model. One thousand replicates were used to generate the bootstrap consensus tree. A discrete Gamma (G) distribution was used for evolutionary rate variations across sites. Numbers on the nodes show the bootstrap support, numbers on the branches show substitutions per site. Orange color represents species where GAD pathway genes are organized in an operon structure. Purple color represents species without an operon structure, and blue color represents species with a small operon structure consisting of only a transporter and a GAD gene.

However, closer examination of interspecies tests showed neutral evolution (no sign of positive selection) in certain comparisons such as *L. brevis gad1* and *gadC* with respect to *gad* and *gadC* in *L. spicheri*, *L. zymae*, *L. sakei*, *L. angrenensis*, and *L. cerevisiae*. *L. brevis gad2* with respect to *L. plantarum gadB*, and with respect to *gad* genes in *L. paraplantarum*, *L. herbarum*, *L. argentoratensis* also did not show positive selection. Similar neutral evolution results were observed for *L. plantarum gadB* with respect to *gad* genes in *L. argentoratensis*, *L. herbarum*, and *S. paracollinoides*. Moreover, negative selection (high conservation) was observed when *L. plantarum gadB* is compared with *L. paraplantarum gad*. To get a broader perspective on the between species sequence comparisons, a phylogenetic tree based on 16S rRNA sequences is constructed to examine the phylogenetic relatedness of 32 bacterial species (Figure 4). This species tree showed that aforementioned neutral evolution and negative selection results are mostly observed in sequence comparisons between closely related species. However, there were exceptions such as *L. brevis gad1* and *L. sakei gad*, *L. brevis gad2* and *L. plantarum gadB*, *L. brevis gad2* and *C. futsaii gad*, etc. where gene sequences from distantly related genera and species showed higher similarity and much lower or no divergence compared to sequences from closely related species. Further phylogenetic analyses with GAD and transporter sequences showed phylogenetic trees with three distinct clades. One of the clades, clade I, colored orange in Figure 4–6, represent species that have *gadC*, *gad*, and *gltx* genes in an operon structure in their genomes. In some of the species this operon structure also includes a regulatory gene such as *gadR*. The second clade, clade II, colored purple in Figure 4–6, represent species that have only the *gad* gene without an operon structure. And, the last smaller blue colored clade, clade III, represent bacteria that has only *gadC* and *gad* in close vicinity to each other like a smaller operon structure. The phylogenetic tree based on transporter sequences showed a net separation between the three clades (Figure 5; Supplementary Figure S9). Clade II species without an operon structure all has the *yjeM* transporter gene. Clade I and clade III species, separated from clade II, all have the *gadC* transporter gene. Interestingly, clade I and clade III species are further separated from each other based on their *gadC* sequence. This suggests that all transporter (*gadC*) genes in bacteria with a true operon structure (clade I) are more closely related to each other and descend from a common ancestor, and all transporter genes in bacteria with a smaller operon like structure (clade III) are more closely related to each other and descent from a different common ancestor. Within group pairwise genetic distance was lowest for clade I group, followed by clade III and clade I groups (Figure 5). The CAI estimates for both transporter genes were high in all species suggesting high expression of transporter genes in all species (Supplementary Table S17). Because the topologies of species phylogenetic tree and transporter phylogenetic tree are different (Figure 4 vs. Figure 5) one cannot clearly suggest evolution of the operon structure in LABs from an ancestor with or without an operon structure, or identify which of the transporter genes is ancestral. Rather, the species tree suggests multiple independent gain or loss of operon structure in the LABs. The discordance between the 16S rRNA gene and transporter phylogenetic trees also explains why some *gadC* and *yjeM* gene sequences from distant genus and species show less divergence (appear to have not changed much) compared to transporter sequences from more closely related species, as these *gadC* and *yjeM* sequences are phylogenetically more closely related to each other than indicated by the 16S rRNA gene phylogenetic tree of the species that they belong to.

Similar to transporter sequence phylogeny, the GAD sequence phylogenetic tree also showed a three clade topology, where GAD sequences are clustered depending on their presence within an operon structure (Figure 6; Supplementary Figure S9). However, different from the transporter phylogenetic tree, some GAD sequences present in the smaller operon structure (clade III, blue colored, species such as *L. antri*, *L. reuteri* GADs) clustered together with GAD sequences that are not in an operon structure (clade II, purple colored species) forming a separate cluster within clade II cluster. Within group pairwise genetic distance was lowest for clade I, followed by clades II and III (Figure 6). These observations suggest all GADs within a true operon structure are more closely related to each other and descend from a common ancestor. However, it is hard to make a similar argument for GADs outside the operon structure since some GADs within a smaller operon clustered together with GADs without an operon structure, and showed the highest within group genetic distance. Interestingly, *L. brevis gad1*, which is present in the operon structure, clustered with GADs that are also in an operon structure (orange colored clade I lineages), whereas *L*. *brevis gad2*, which is present outside the operon structure, clustered with GADs from species without an operon structure (purple colored clade II lineages). Moreover, there is over 80% amino acid sequence similarity between *L*. *brevis gad2* and other *gad* sequences in the purple group. Taken together with the fact that *gad1* and *gad2* sequences within *L. brevis* populations cluster into two well supported distinct clades strongly suggest that the origin of *gad2* in *L*. *brevis* is not a gene duplication event, but a horizontal gene transfer of a *gad* gene from a LAB without an operon structure. CAI estimates for all GAD genes were high in all species suggesting high expression of decarboxylase enzyme genes in all species (Supplementary Table S17).

The discordance between the 16S species and GAD trees (Figures 4, 6) suggests that molecular evolution of GADs does not follow speciation events, and one cannot surely infer the most ancestral GAD form and molecular evolution of GADs from the species tree. Moreover, horizontal gene transfer events, and possible multiple independent gain or loss of operon structure in LAB species can be another factor driving the molecular evolution of GADs. The discordance between the 16S and GAD phylogenetic trees also explains why some GAD sequences from distant genus and species show less divergence (appear to have not changed much) compared to GAD sequences from more closely related species, as these GAD sequences are phylogenetically more closely related to each other than indicated by the 16S phylogenetic tree of the species that they belong to.

## 4 Discussion

We aimed to study the molecular evolution and population genetics of GAD pathway genes in LABs to understand selective processes shaping adaptation to low pH environments comparing gene sequences from species where the GAD pathway genes are organized in an operon structure (*L. brevis*) versus lack of an operon structure (*L. plantarum*). Within species analyses focused on gene sequences belonging to specific strains with whole genome sequence sampled from diverse food and non-food environments. Between species analyses examined the distribution of operon structure on the LAB species tree, and compared the divergence and molecular evolution of GAD pathway genes between closely and distantly related LAB species.

Nucleotide based analyses with six GAD pathway genes from *L. brevis* and *L. plantarum* indicated that these are actively transcribed genes under negative selection against replacement changes. Amino acid characteristics and protein structure analyses also indicated purifying selection eliminating amino acid changes with possible drastic effects in both species. Between species analyses with GAD pathway genes showed excessive fixed amino acid differences between species indicating adaptive protein evolution. One can speculate that there is fast adaptive protein evolution in the GAD pathway enabling a species to adapt to a new environment after a speciation or gene gain/loss event. After the adaptive protein evolution there is conservation of genes within the species. Another conclusion driven by between species comparisons is that GAD pathway genes can be transferred between LABs *via* horizontal gene transfer. Within *L. brevis* populations *gad1* and *gad2* sequences clustered into two well supported distinct clades. And, phylogenetic analyses with different species’ GAD genes showed clustering of *L. brevis gad1* with the *gad* sequences of LABs with an operon structure, whereas *L. brevis gad2* grouped with gad sequences of LABs without an operon structure. Finally, the distribution of operon structure on the LAB species tree suggests multiple independent gain or loss of operon structure in LABs independent of speciation events.

Several of our findings are in agreement with functional studies. *L. brevis* GAD system mutagenesis studies showed that *L. brevis* operon is acid-sensitive, and has a much bigger role in acid resistance compared to *gad2* that is outside the operon (Shin et al., 2014; Lyu et al., 2018). We observed higher conservation and higher codon usage bias in *L. brevis* genes inside the operon structure compared to *gad2*, supporting the higher expression of and higher selection on the operon structure in *L. brevis*. Similar functional studies in other LAB species is not reported. However, we observed higher conservation for GAD genes within an operon structure in the other examined LAB species as well. This may suggest that LABs with an operon structure utilize their operon structure much more efficiently for acid resistance, and can be more resistant to low pH environments. The biochemical characteristics, thermostability, and enzyme activity of *L. brevis* Gad2 is reported to be closer to *L. plantarum* GadB compared to *L. brevis* Gad1 (Wu et al., 2017). We also observed higher amino acid sequence similarity between *L. brevis* Gad2 and *L*. *plantarum* GadB compared to *L*. *brevis* Gad1 (84% vs. 52% sequence similarity). The C-terminal regions of *L*. *brevis* Gad2 and *L*. *plantarum* GadB form a C-plug structure that curls toward the active site (Lyu et al., 2021). The C-terminal regions are pH dependent, and can adjust the catalytic activity of the enzyme according to pH change (Shin et al., 2014), indicating the importance of C-terminal region in enzyme activity. In this study, the predicted 3D structures of *L*. *brevis* Gad2 and *L*. *plantarum* GadB were nearly identical, both having a C-terminal region curving towards the active site. Moreover, the C-terminal regions of Gad2 and GadB were highly conserved. A similar C-terminal structure that curls toward the active site is not observed in Gad1, and the C-terminal region of Gad1 was less conserved compared to the C-terminal region of Gad2. The quaternary structures of Gad2 and GadB enzymes in the active state are reported to be monomer (Shi et al., 2014) and dimer (Shin et al., 2014), respectively. Whereas, *L*. *brevis* Gad1 is reported to be active in a tetramer structure (Hiraga et al., 2008). N-terminus residues are involved in proper Gad1 quaternary structure formation (Hiraga et al., 2008). The importance and high conservation of the N-terminus residues was evident in our observations because we did not observe any amino acid changes in the Gad1 N-terminus residues involved in quaternary structure formation.

We could only find the *E*. *coli* GadC transporter 3D structure in the databases. *E*. *coli* GadC has a C-plug structure that goes through a conformational change according to intracellur pH and controls transport of ions (Ma et al., 2012). Our 3D structure prediction of *L*. *brevis* GadC is similar to *E*. *coli* GadC, and a possible C-plug structure is observed. The possible C-plug residues in *L*. *brevis* GadC was also highly conserved. A possible C-plug structure was not observed in the YjeM transporter, and most amino acid changes were observed on the C-terminal residues of YjeM proteins.

There are several limitations of our study. First, we only focused on LABs primarily found in fermented food environments. Because LABs not only survive but thrive and out compete other bacteria in low pH environments, we hypothesized that LABs would be one of the best bacteria to study adaptation to acidic environments. However, GAD pathway is also a major acid resistance pathway utilized by pathogenic bacteria such as *E*. *coli* (Bhagwat et al., 2005; Bergholz et al., 2007; Chen et al., 2018), *Shigella* sp. (Waterman and Small, 2003), *Listeria* sp. (Cotter et al., 2001; Cotter et al., 2001; Karatzas et al., 2012), and *Brucella* sp. (Damiano et al., 2015; Grassini et al., 2015). Moreover, GAD pathway can be an important virulence and other stress resistance factor (Feehily and Karatzas, 2013) so similar population genetics and molecular evolution studies should be conducted in other bacteria. Additionally, bacteria have other acid resistance mechanisms, in addition to the GAD pathway (Krulwich et al., 2011; Lund et al., 2014). The genes involved in other acid resistance mechanisms should be investigated to validate our conclusions. Moreover, possible interactions between different acid resistance pathways can be yet another factor in the evolution of acid resistance, that should be investigated in future studies. Finally, the conclusions we reach on the effects of observed amino acid changes on the protein charge distribution, isoelectric point, and structure are just predictions. The actual effects of these changes on protein structure and function should be experimentally confirmed.

We conclude that the evolution of acid resistance in LABs is shaped by adaptive protein evolution and acquisition of new genes during adaptation to low pH environments, followed by conservation of protein function and activity. Our results present another step in understanding the evolutionary mechanisms driving the molecular mechanisms behind acid resistance, and have potential broader implications by stimulating similar studies in other microorganisms.

## Data Availability

The datasets presented in this study can be found in online repositories. The names of the repository/repositories and accession number(s) can be found in the article/Supplementary Material.
