# Supplementary material for: Molecular evolution and population genetics of glutamate decarboxylase acid resistance pathway in lactic acid bacteria
Source: Front Genet. 2023 Jan 26;14:1027156. doi: 10.3389/fgene.2023.1027156 (PMC9909107; doi:10.3389/fgene.2023.1027156)
Supplement: Supplementary file 1 [file Table1.DOCX]

**Supplementary Table 1-A:** Strain information, isolation source, genome or contig accession number, and analysis group (population) assignment of *L. brevis* strains used in the study.

| **Sequence Mode** | **Name** | **Source** | **Accession Number** | **Population** |
| --- | --- | --- | --- | --- |
| Complete Genome | *Levilactobacillus brevis NPS-QW-145* | Kimchi(Hong Kong) | CP015398 |  |
| Contig | *Levilactobacillus brevis D7* | Kimchi(South Korea) | NVYO01000001 | **Fermented** |
| Contig | *Levilactobacillus brevis G101* | Kimchi | MULL01000001 | **Vegetable** |
| Contig | *Levilactobacillus brevis SRCM103306* | Food(South Korea) | SBJN01000006 | **Group** |
| Complete Genome | *Levilactobacillus brevis 100D8* | Rye silage(South K) | CP015338 |  |
| Complete Genome | *Levilactobacillus brevis KB290* | Suguki(Fermented vegetable) | AP012167 |  |
| Contig | *Levilactobacillus brevis CRL2013* | Sourdough(Argentina) | MZMW01000001 |  |
| Contig | *Levilactobacillus brevis TR055* | Sourdough(Ireland) | QFDK01000006/03 | **Sourdough** |
| Contig | *Levilactobacillus brevis TR052* | Sourdough(Ireland) | QFDL01000013/03 | **Group** |
| Contig | *Levilactobacillus brevis TR169* | Sourdough(Ireland) | QFDG01000024/16 |  |
| Complete Genome | *Levilactobacillus brevis UCCLBBS124* | Beer Keg | CP031169 |  |
| Complete Genome | *Levilactobacillus brevis TMW 1.2108* | Wheat beer(Germany) | CP019734 |  |
| Complete Genome | *Levilactobacillus brevis TMW 1.2112* | Wheat beer(Germany) | CP016797 |  |
| Complete Genome | *Levilactobacillus brevis NSMJ23* | Alcoholic Beverage | CP050541 | **Fermented** |
| Complete Genome | *Levilactobacillus brevis UCCLB521* | Brewery environment | CP031208 | **Beverage** |
| Contig | *Levilactobacillus brevis TMW 1.465* | Brewery environment | JXUG01000003 | **Group** |
| Contig | *Levilactobacillus brevis TMW 1.313* | Beer | JXUF01000002 |  |
| Complete Genome | *Levilactobacillus brevis UCCLBBS449* | Beer | CP031198 |  |
| Contig | *Levilactobacillus brevis BSO 310* | Brewery | LGIX01000030/17 |  |
| Complete Genome | *Levilactobacillus brevis BSO 464* | Brewery | CP005977 |  |
| Contig | *Levilactobacillus brevis TUCO-5E* | Milk | QMCB01000017/01 |  |
| Complete | *Levilactobacillus brevis HQ1-1* | dairy products | CP046631 | **Fermented** |
| Contig | *Levilactobacillus brevis D6* | Cheese | LQNG01000043/24 | **Dairy product** |
| Contig | *Levilactobacillus brevis KMB_620* | Bryndza cheese | QMJT01000025/48 | **Group** |
| Contig | *Levilactobacillus brevis KMB_615* | Bryndza cheese | QMJO01000051/32 |  |
| Contig | *Levilactobacillus brevis 47f* | Feces(Homo sapiens) | LBHR01000030/01 |  |
| Contig | *Levilactobacillus brevis 15f* | Feces(Homo sapiens) | JXCD01000017/02 |  |
| Contig | *Levilactobacillus brevis TMW 1.6* | Feces | JXUE01000015 | **Feces** |
| Complete Genome | *Levilactobacillus brevis ZLB004* | Feces (pig) | CP021456 | **Group** |
| Contig | *Levilactobacillus brevis DPC 6108* | Feces(Homo sapiens) | MDUA01000001 |  |

**Supplementary Table 1-B:** Strain information, isolation source, genome or contig accession number, and analysis group (population) assignment of *L. plantarum* strains used in the study.

| **Sequence Mode** | **Name** | **Source** | **Accession Number** | **Population** |
| --- | --- | --- | --- | --- |
| Complete Genome | *Lactiplantibacillus plantarum CAUH2* | Sichuan pickled vegetables | CP015126 |  |
| Complete Genome | *Lactiplantibacillus plantarum JBE245* | fermented soybean (South Korea) | CP014780 |  |
| Complete Genome | *Lactiplantibacillus plantarum CNEI-KCA5* | Fermented Okpei-Nsukka | CP059294 |  |
| Complete Genome | *Lactiplantibacillus plantarum BK-021* | Fermented onions (South Korea) | CP044233 |  |
| Complete Genome | *Lactiplantibacillus plantarum CNEI-KCA4* | Fermented Okpei-Onitsha | CP053571 |  |
| Complete Genome | *Lactiplantibacillus plantarum AMT74419* | Kimchi (South Korea) | CP052869 |  |
| Complete Genome | *Lactiplantibacillus plantarum KCCP11226* | Kimchi (South Korea) | CP046262 |  |
| Complete Genome | *Lactiplantibacillus plantarum EM* | Kimchi (South Korea) | CP037429 |  |
| Complete Genome | *Lactiplantibacillus plantarum ATG-K6* | Kimchi (South Korea) | CP032464 |  |
| Complete Genome | *Lactiplantibacillus plantarum ATG-K2* | Kimchi (South Korea) | CP032460 |  |
| Complete Genome | *Lactiplantibacillus plantarum IDCC3501* | Kimchi (South Korea) | CP031702 |  |
| Complete Genome | *Lactiplantibacillus plantarum b-2* | Pickle (China) | CP027349 |  |
| Complete Genome | *Lactiplantibacillus plantarum DSR_M2* | Kimchi (South Korea) | CP022294 | **Kimchi** |
| Complete Genome | *Lactiplantibacillus plantarum HAC01* | White Kimchi (South Korea) | CP029349 | **Group** |
| Complete Genome | *Lactiplantibacillus plantarum KC28* | Kimchi (South Korea) | CP026743 |  |
| Complete Genome | *Lactiplantibacillus plantarum LM1004* | Cabbage kimchi (South Korea) | CP025988 |  |
| Complete Genome | *Lactiplantibacillus plantarum PC520* | Chinese fermented food-pickles | CP023772 |  |
| Complete Genome | *Lactiplantibacillus plantarum NCU116* | Chinese pickle | CP016071 |  |
| Complete Genome | *Lactiplantibacillus plantarum DSM 20174* | Pickled cabbage | CP039121 |  |
| Complete Genome | *Lactiplantibacillus plantarum SRCM101511* | Radish Kimchi (South Korea) | CP028235 |  |
| Complete Genome | *Lactiplantibacillus plantarum SRCM102737* | Soybean paste (South Korea) | CP028261 |  |
| Complete Genome | *Lactiplantibacillus plantarum SRCM101518* | Radish Kimchi (South Korea) | CP028241 |  |
| Complete Genome | *Lactiplantibacillus plantarum SRCM101222* | Radish Kimchi (South Korea) | CP028229 |  |
| Complete Genome | *Lactiplantibacillus plantarum SRCM101187* | White Kimchi (South Korea) | CP028226 |  |
| Complete Genome | *Lactiplantibacillus plantarum SRCM101167* | Water kimchi (South Korea) | CP028334 |  |
| Complete Genome | *Lactiplantibacillus plantarum SRCM101105* | Kimchi (South Korea) | CP028222 |  |
| Complete Genome | *Lactiplantibacillus plantarum SRCM100995* | Pickled Green Chili Peppers | CP028275 |  |
| Complete Genome | *Lactiplantibacillus plantarum PMO08* | Kimchi (South Korea) | CP062059 |  |

**Supplementary Table 1-B:** (cont.).

| **Sequence Mode** | **Name** | **Source** | **Accession Number** | | **Population** |
| --- | --- | --- | --- | --- | --- |
| Complete Genome | *Lactiplantibacillus plantarum SRCM103297* | Food (South Korea) | CP035556 |  | |
| Complete Genome | *Lactiplantibacillus plantarum SRCM103473* | Food (South Korea) | CP035224 |  | |
| Complete Genome | *Lactiplantibacillus plantarum SRCM103472* | Food (South Korea) | CP035223 |  | |
| Complete Genome | *Lactiplantibacillus plantarum SRCM103426* | Food (South Korea) | CP035174 | **Unspecified** | |
| Complete Genome | *Lactiplantibacillus plantarum SRCM103418* | Food (South Korea) | CP035168 | **Femented Food** | |
| Complete Genome | *Lactiplantibacillus plantarum SRCM103357* | Food (South Korea) | CP035143 | **Group** | |
| Complete Genome | *Lactiplantibacillus plantarum SRCM103362* | Food (South Korea) | CP035156 |  | |
| Complete Genome | *Lactiplantibacillus plantarum SRCM103295* | Food (South Korea) | CP035113 |  | |
| Complete Genome | *Lactiplantibacillus plantarum AS-9* | Fruits and Vegetables | CP028421 |  | |
| Complete Genome | *Lactiplantibacillus plantarum AS-6* | Fruits and Vegetables | CP028424 | **Raw** | |
| Complete Genome | *Lactiplantibacillus plantarum AS-8* | Fruits and Vegetables | CP028422 | **Foods** | |
| Complete Genome | *Lactiplantibacillus plantarum AS-10* | Fruits and Vegetables | CP028420 |  | |
| Complete Genome | *Lactiplantibacillus plantarum 12_3* | Tibet kefir (China) | CP035012 |  | |
| Complete Genome | *Lactiplantibacillus plantarum YW11* | Tibet kefir (China) | CP035031 |  | |
| Complete Genome | *Lactiplantibacillus plantarum 13_3* | Tibet kefir (China) | CP035020 |  | |
| Complete Genome | *Lactiplantibacillus plantarum Q7* | Yak fer. milk(China) | CP019712 | **Kefir** | |
| Contig | *Lactiplantibacillus plantarum YW32* | Tibet kefir (China) | SDKA01000009/15 | **Group** | |
| Contig | *Lactiplantibacillus plantarum B-1* | Tibet kefir (China) | SDJY01000003/04 |  | |
| Contig | *Lactiplantibacillus plantarum XZ3303* | Tibet kefir (China) | SDJU01000003/13 |  | |
| Contig | *Lactiplantibacillus plantarum C4* | Kefir (Spain) | PVNN01000002/06 |  | |
| Contig | *Lactiplantibacillus plantarum SKT109* | Tibet kefir (China) | SAZE01000001/05 |  | |

**Supplementary Table 1-B:** (cont.).

| **Sequence Mode** | **Name** | **Source** | **Accession Number** | **Population** |
| --- | --- | --- | --- | --- |
| Complete Genome | *Lactiplantibacillus plantarum 10CH* | Cheese (UK) | CP023728 |  |
| Complete Genome | *Lactiplantibacillus plantarum NCIMB 700965* | New Zealand cheese | CP023490 |  |
| Contig | *Lactobacillus plantarum UCMA 3037* | Cheese (France) | APHP01000021/05 |  |
| Contig | *Lactiplantibacillus plantarum YLBGNL-S7* | Traditional cheese (China) | VHJC01000067/05 | **Cheese** |
| Contig | *Lactiplantibacillus plantarum LL441* | Traditional cheese (Spain) | LWKN01000058/80 | **Group** |
| Contig | *Lactiplantibacillus plantarum KMB_597* | Ovine cheese (Slovakia) | QMJE01000007/02 |  |
| Contig | *Lactiplantibacillus plantarum KMB_614* | Bryndza cheese (Slovakia) | QMJN01000031/08 |  |
| Contig | *Lactiplantibacillus plantarum KMB_619* | Bryndza cheese (Slovakia) | QMJS01000088/108 |  |
| Contig | *Lactiplantibacillus plantarum KMB_618* | Bryndza cheese (Slovakia) | QMJR01000039/45 |  |
| Contig | *Lactiplantibacillus plantarum KMB_621* | Bryndza cheese (Slovakia) | QMJU01000031/28 |  |
| Contig | *Lactiplantibacillus plantarum CECT 9435* | Chicha | CAADHQ010000026/09 |  |
| Contig | *Lactiplantibacillus plantarum 9434* | Chicha | CAADEV010000026/09 |  |
| Contig | *Lactiplantibacillus plantarum 8965* | Chicha | OMOO01000005/04 | **Beverage** |
| Complete Genome | *Lactiplantibacillus plantarum TMW 1.277* | Palm wine (Germany) | CP017363 | **Group** |
| Complete Genome | *Lactiplantibacillus plantarum UNQLp11* | Pinot noir wine (Argentina) | CP031140 |  |
| Complete Genome | *Lactiplantibacillus plantarum JBE490* | Nuruk(Korea) | CP020861 |  |
| Complete Genome | *Lactiplantibacillus plantarum MF1298* | Fermented sausage (Norway) | CP013149 |  |
| Complete Genome | *Lactiplantibacillus plantarum TMW 1.708* | raw sausage (Germany) | CP017374 |  |
| Complete Genome | *Lactiplantibacillus plantarum TMW 1.25* | Raw sausage (Germany) | CP017354 | **Meat** |
| Contig | *Lactiplantibacillus plantarum CRL 681* | Fermented sausage (Argentina) | QOSF01000014/13 | **Group** |
| Contig | *Lactiplantibacillus plantarum A6* | Vietnamese fermented sausage | LRUO01000066/03 |  |
| Complete Genome | *Lactiplantibacillus plantarum LPL-1* | fermented fish (China) | CP021997 |  |

**Supplementary Table 1-B:** (cont.).

| **Sequence Mode** | **Name** | **Source** | **Accession Number** | **Population** |
| --- | --- | --- | --- | --- |
| Complete Genome | *Lactiplantibacillus plantarum pc-26* | Healthy adult fecal sample (China) | CP023301 |  |
| Complete Genome | *Lactiplantibacillus plantarum Heal19* | Human GI tract (Sweden) | CP055123 | **Feces** |
| Complete Genome | *Lactiplantibacillus plantarum FBL-3a* | Fecal sample (China) | CP034694 | **Group** |
| Complete Genome | *Lactiplantibacillus plantarum 83-18* | Human feces (Russia) | CP046669 |  |
| Complete Genome | *Lactiplantibacillus plantarum 123-17* | Human feces (Russia) | CP046656 |  |
| Complete Genome | *Lactiplantibacillus plantarum DR7* | Milk (Malaysia) | CP031318 | **Other**  **Milk Based**  **Fermented**  **Products** |
| Complete Genome | *Lactiplantibacillus plantarum LZ227* | Raw cow milk (China) | CP015857 |  |
| Complete Genome | *Lactiplantibacillus plantarum LZ206* | Raw cow milk (China) | CP015966 |  |
| Complete Genome | *Lactiplantibacillus plantarum TS12* | Stinky Tofu (Malaysia) | CP018324 |  |
| Complete Genome | *Lactiplantibacillus plantarum X7021* | Brine of stinky tofu (China) | CP025412 |  |
| Contig | *Lactiplantibacillus plantarum T9* | Milk tofu (China) | RPOJ01000071/05 |  |
| Complete Genome | *Lactiplantibacillus plantarum 2025* | Milk products (Russia) | CP076824 |  |
| Complete Genome | *Lactiplantibacillus plantarum TK-P2A* | Probitic product (China) | CP045593 |  |
| Complete Genome | *Lactiplantibacillus plantarum 8P-A3* | Probiotic preparation (Russia) | CP046726 |  |
| Contig | *Lactiplantibacillus plantarum CCFM605* | Yogurt-like dairy product (China) | QPQR01000004/03 |  |
| Contig | *Lactiplantibacillus plantarum CGMCC12436* | Yogurt-like dairy product (China) | QPQS01000007/02 |  |
| Complete Genome | *Lactiplantibacillus plantarum J26* | Fermented dairy products (China) | CP033616 |  |

**Supplementary Table 2:** Glutamate decarboxylase protein and nucleotide sequence accession numbers, and isolation source information of 32 bacterial species.

| **Organisms** | **Protein Accession No.** | **Nucleotide Accession No.** | **Source** |
| --- | --- | --- | --- |
| *Levilactobacillus brevis* | ANN50035/ANN49747 | CP015398 | Korean kimchi |
| *Levilactobacillus zymae* | AHF72525 | KF690143 | Kimchi |
| *Levilactobacillus senmaizukei* | KRN01061 | AYZH01000037 | Pickles |
| *Levilactobacillus tangyuanensis* | WP_125641322 | NZ_RHOA01000007 | Pickle |
| *Levilactobacillus angrenensis* | WP_125574762 | NZ_RHOB01000001 | Yogurt |
| *Levilactobacillus cerevisiae* | WP_125583210 | NZ_RHNN01000018 | Spoiled beer |
| *Levilactobacillus spicheri* | KJW12820 | JZCR01000015 | Food |
| *Lactiplantibacillus plantarum* | AWY49471 | CP022294 | Kimchi |
| *Lactiplantibacillus argentoratensis* | AYJ36890 | CP032751 | Fermented cassava roots |
| *Lactiplantibacillus paraplantarum* | AYJ39960 | CP032744 | Beer |
| *Lactiplantibacillus herbarum* | WP_048001054 | NZ_LFEE01000043 | Fermented radish |
| *Limosilactobacillus reuteri* | KEQ19823 | JOKX02000003 | Type II sourdough |
| *Limosilactobacillus fermentum* | BAO00911 | AB856984 | Pickle juice |
| *Limosilactobacillus oris* | KRM16609 | AZGE01000002 | Saliva |
| *Limosilactobacillus antri* | EEW54005 | ACLL01000020 | - |
| *Limosilactobacillus gastricus* | EHS85882 | NZ_AICN01000052 | Human milk |
| *Limosilactobacillus coleohominis* | KRM84158 | AZEW01000005 | vagina |
| *Latilactobacillus sakei* | AJR27923 | KM982734 | Fermented sea-food |
| *Latilactobacillus curvatus* | ASN62676 | CP022475 | Salami |
| *Lentilactobacillus parakefiri* | GAW73186 | BDGB01000146 | Kefir grain |
| *Lentilactobacillus buchneri* | AEB72391 | CP002652 | - |
| *Companilactobacillus nuruki* | PMD69826 | NIPR01000025 | Beverage starter |
| *Companilactobacillus futsaii* | BBA26472 | AB986192 | Fermented shrimp |
| *Lacticaseibacillus paracasei* | BAG12190 | AB295641 | Fermented fish |
| *Loigolactobacillus rennini* | WP_057874537 | NZ_AYYI01000075 | Rennin |
| *Paucilactobacillus suebicus* | KRM13113 | AZGF01000003 | Apple mash |
| *Furfurilactobacillus rossiae* | KRL56399 | AZFF01000004 | Wheat sourdough |
| *Secundilactobacillus paracollinoides* | ANZ65502 | CP014915 | Brewery |

**Supplementary Table 2:** (cont.)

| **Organisms** | **Protein Accession No.** | **Nucleotide Accession No.** | **Source** |
| --- | --- | --- | --- |
| *Lactococcus lactis* | QEA60644 | CP042408 | Kimchi |
| *Enterococcus faecium* | KST45897 | LKPH01000077 | Cheese |
| *Streptococcus thermophilus* | QTA50001 | CP061019 | Cheese starter |
| *Escherichia coli* | CAA0191038/  CAA0194968 | CACSHO010000110/  CACSHO010000111 | Urine |

**Supplementary Table 3:** Glutamate/gamma-aminobutyrate transporter (*gadC* or *yjeM*) protein and nucleotide sequence accession numbers, and isolation source information of 32 bacterial species.

| **Organisms** | **Protein Accession** | **Nucleotide Accession** | **Source** | **Name** |
| --- | --- | --- | --- | --- |
| *Levilactobacillus brevis* | ANN48031 | CP015398 | Korean kimchi | *gadC* |
| *Levilactobacillus zymae* | AHF72526 | KF690144 | Kimchi | *gadC* |
| *Levilactobacillus senmaizukei* | KRN01060 | AYZH01000037 | Pickles | *gadC* |
| *Levilactobacillus tangyuanensis* | WP_125641325 | NZ_RHOA01000007 | Pickle | *gadC* |
| *Levilactobacillus angrenensis* | WP_125574761 | NZ_RHOB01000001 | Yogurt | *gadC* |
| *Levilactobacillus cerevisiae* | WP_125583211 | NZ_RHNN01000018 | Spoiled beer | *gadC* |
| *Levilactobacillus spicheri* | KJW12731 | JZCR01000015 | Food | *gadC* |
| *Lactiplantibacillus plantarum* | AWY49189 | CP022294 | Kimchi | *yjeM* |
| *Lactiplantibacillus argentoratensis* | AYJ36618 | CP032751 | Fermented cassava roots | *yjeM* |
| *Lactiplantibacillus paraplantarum* | AYJ39671 | CP032744 | Beer contaminant | *yjeM* |
| *Lactiplantibacillus herbarum* | - | - | - | *-* |
| *Limosilactobacillus reuteri* | KEQ19824 | JOKX02000003 | Type II sourdough | *gadC* |
| *Limosilactobacillus fermentum* | WP_104878675 | NZ_LT906621 | Sourdough | *yjeM* |
| *Limosilactobacillus oris* | KRM16610 | AZGE01000002 | Saliva | *gadC* |
| *Limosilactobacillus antri* | EEW54006 | ACLL01000020 | - | *gadC* |
| *Limosilactobacillus gastricus* | WP_007122298 | NZ_AICN01000047 | Human milk | *yjeM* |
| *Limosilactobacillus coleohominis* | KRM81387 | AZEW01000121 | Vagina | *gadC* |
| *Latilactobacillus sakei* | AKE47364 | KP310071 | Fermented sea-food | *gadC* |
| *Latilactobacillus curvatus* | ASN62677 | CP022475 | Salami | *gadC* |
| *Lentilactobacillus parakefiri* | GAW73185 | BDGB01000146 | Kefir grain | *gadC* |
| *Lentilactobacillus buchneri* | AEB72390 | CP002652 | - | *gadC* |
| *Companilactobacillus nuruki* | PMD67803 | NIPR01000064 | Beverage starter | *yjeM* |
| *Companilactobacillus futsaii* | QCX25054 | CP040736 | - | *yjeM* |
| *Lacticaseibacillus paracasei* | - | - | - | *-* |
| *Loigolactobacillus rennini* | WP_057874538 | NZ_AYYI01000075 | Rennin | *gadC* |
| *Paucilactobacillus suebicus* | - | - | - | *-* |
| *Furfurilactobacillus rossiae* | KRL56398 | AZFF01000004 | Wheat sourdough | *gadC* |
| *Secundilactobacillus paracollinoides* | ANZ62949 | CP014915 | Brewery environment | *yjeM* |
| *Lactococcus lactis* | QEA60645 | CP042408 | Kimchi | *gadC* |
| *Enterococcus faecium* | KST45898 | LKPH01000077 | Cheese | *gadC* |

**Supplementary Table 3:** (cont.)

| **Organisms** | **Protein Accession** | **Nucleotide Accession** | **Source** | **Name** |
| --- | --- | --- | --- | --- |
| *Streptococcus thermophilus* | QTA50002 | CP061019 | Cheese starter | *gadC* |
| *Escherichia coli* | CAA0194932 | CACSHO010000111 | Urine | *gadC* |
|  |  |  |  |  |

**Supplementary Table 4:** 16S rRNA gene information of 32 bacterial species.

| **16S rRNA Accession** | **Organisims** | **bp** | **Source** |
| --- | --- | --- | --- |
| KX458105.1 | *Levilactobacillus brevis* | 1508 bp | Kimchi |
| KJ607887.1 | *Levilactobacillus zymae* | 1543 bp | Sourdough |
| MT898562.1 | *Levilactobacillus senmaizukei* | 1593 bp | Cucumber kimchi |
| MK110861.1 | *Levilactobacillus tangyuanensis* | 1432 bp | Pickle |
| MK110858.1 | *Levilactobacillus angrenensis* | 1445 bp | Yogurt |
| MT211345.1 | *Levilactobacillus cerevisiae* | 1489 bp | Wufeng pickle water |
| KT757220.1 | *Levilactobacillus spicheri* | 1476 bp | Fermented bamboo shoot |
| MT898568.1 | *Lactiplantibacillus plantarum* | 1522 bp | Cabbage kimchi |
| MZ959460.1 | *Lactiplantibacillus argentoratensis* | 1490 bp | From koji |
| MZ365311.1 | *Lactiplantibacillus paraplantarum* | 1520 bp | Kimchi |
| MH548359.1 | *Lactiplantibacillus herbarum* | 1505 bp | Milk |
| L23507.1 | *Limosilactobacillus reuteri* | 1535 bp | - |
| AB856983.1 | *Limosilactobacillus fermentum* | 1491 bp | Chinese pickle juice |
| X94229.1 | *Limosilactobacillus oris* | 1512 bp | sourdough |
| AY253659.1 | *Limosilactobacillus antri* | 1520 bp | - |
| AY253658.1 | *Limosilactobacillus gastricus* | 1550 bp | - |
| AM113776.1 | *Limosilactobacillus coleohominis* | 1564 bp | - |
| KX886806.1 | *Latilactobacillus sakei* | 1496 bp | Kimchi |
| MT898647.1 | *Latilactobacillus curvatus* | 1590 bp | Cabbage kimchi |
| LC096211.1 | *Lentilactobacillus parakefiri* | 1514 bp | - |
| OK135530.1 | *Lentilactobacillus buchneri* | 1500 bp | Zha-chili |
| MT786425.1 | *Companilactobacillus nuruki* | 1419 bp | Bioproduct |
| AB839950.1 | *Companilactobacillus futsaii* | 1528 bp | Fermented shrimp |
| OK559730.1 | *Lacticaseibacillus paracasei* | 1498 bp | Dairy product |
| LC258150.1 | *Loigolactobacillus rennini* | 1514 bp | - |
| KU761840.1 | *Paucilactobacillus suebicus* | 1573 bp | Sichuan pickle |
| MZ749583.1 | *Furfurilactobacillus rossiae* | 1496 bp | From acidic gruel |
| NR_112846.1 | *Secundilactobacillus paracollinoides* | 1525 bp | Brewery environments |
| MN749817.1 | *Lactococcus lactis* | 1546 bp | Kimchi |
| KM495946.1 | *Enterococcus faecium* | 1495 bp | Siahmazgi cheese |
| HM218518.1 | *Streptococcus thermophilus* | 1472 bp | Fermented dairy products |
| MW059027.1 | *Escherichia coli* | 1464 bp | - |

**Supplementary Table 5:** Population genetic summary statistics for nucleotide diversity of glutamate decarboxylase pathway genes among *Levilactabacillus brevis* population.

| **Population**  ***L .brevis*** | | **N** | **Total bases** | **Syn. sites** | **Nonsyn. sites** | **S(Eta)** | **Sing.** | **Par.** | **Syn. Pol.** | **Rep. Pol.** | **π (Pi) All Sites** | **Theta-W**  **All Sites**  **(**θ) | **π (JC)**  **All**  **Sites** | **π (JC) Syn. sites** | **π (JC) Nonsyn. sites** |
| --- | --- | --- | --- | --- | --- | --- | --- | --- | --- | --- | --- | --- | --- | --- | --- |
| **All Samples** | *Operon* | 30 | 3516  (3771) | 807.32  (1058) | 2708.68 | 214(217) | 104 | 110 | 175 | 27 | 112 | 143.2 | 113.3 | 485.1  (387.2) | 9.4 |
|  | *gadR* |  | 591 | 135.36 | 455.64 | 28(28) | 9 | 19 | 23 | 5 | 107.4 | 119.6 | 108.4 | 452.9 | 10 |
|  | *gadC* |  | 1492 | 346.71 | 1141.29 | 112(114) | 65 | 47 | 101 | 12 | 129.6 | 189.5 | 131.8 | 561.2 | 9 |
|  | *gad1* |  | 1440 | 325.26 | 1111.75 | 60(61) | 20 | 40 | 51 | 10 | 100.4 | 105.2 | 101.3 | 427.4 | 9.7 |
|  | *gad2* |  | 1401 | 321.24 | 1076.76 | 76(77) | 32 | 44 | 59 | 18 | 123.5 | 136.9 | 125 | 451.7 | 31.9 |
| **Fermented Vegetable**  **Group** | *Operon* | 6 | 3534  (3785) | 810.89  (1062) | 2723.11 | 59(59) | 32 | 27 | 51 | 6 | 67.8 | 68.3 | 68.2 | 279.4  (222.6) | 9.1 |
|  | *gadR* |  | 591 | 135.06 | 455.94 | 12(12) | 5 | 7 | 12 | 0 | 92.5 | 88.9 | 93.2 | 419 | - |
|  | *gadC* |  | 1506 | 350.53 | 1155.47 | 23(23) | 13 | 10 | 20 | 3 | 67.7 | 66.9 | 68.1 | 260.4 | 11 |
|  | *gad1* |  | 1440 | 325.31 | 1111.69 | 22(22) | 14 | 8 | 19 | 3 | 62 | 66.9 | 62.5 | 245 | 10.8 |
|  | *gad2* |  | 1401 | 320.81 | 1077.19 | 31(31) | 19 | 12 | 23 | 8 | 90.9 | 96.9 | 91.8 | 311.8 | 28.6 |
| **Sourdough**  **Group** | *Operon* | 4 | 3534  (3785) | 811.13  (1062) | 2722.88 | 91(91) | 70 | 21 | 77 | 9 | 129.5 | 131.1 | 130.7 | 537.9  (431.9) | 16.6 |
|  | *gadR* |  | 591 | 135.38 | 455,63 | 15(15) | 14 | 1 | 11 | 4 | 129.7 | 138.4 | 131 | 432.6 | 44.1 |
|  | *gadC* |  | 1506 | 350.58 | 1155.42 | 35(35) | 27 | 8 | 33 | 2 | 125.1 | 126.8 | 126.3 | 530.4 | 8.7 |
|  | *gad1* |  | 1440 | 325.17 | 1111.83 | 36(36) | 24 | 12 | 33 | 3 | 138.9 | 136.4 | 140.3 | 593 | 13.5 |
|  | *gad2* |  | 1407 | 322.63 | 1081.38 | 36(36) | 29 | 7 | 28 | 8 | 136.2 | 139.6 | 137.8 | 484.2 | 38.7 |
| **Fermente Dairy**  **Products** | *Operon* | 5 | 3516  (3771) | 807.95  (1058) | 2708.97 | 86(86) | 58 | 28 | 78 | 5 | 106.1 | 109.5 | 107.1 | 470.8  (371.2) | 7.4 |
|  | *gadR* |  | 591 | 135.03 | 455.97 | 16(16) | 8 | 8 | 14 | 2 | 135.4 | 129.9 | 136.9 | 557.9 | 17.6 |
|  | *gadC* |  | 1492 | 346.67 | 1141.33 | 37(37) | 26 | 11 | 37 | 0 | 113.9 | 119 | 115.2 | 515.2 | - |
|  | *gad1* |  | 1440 | 325.33 | 1111.67 | 30(30) | 23 | 7 | 27 | 3 | 93.1 | 100 | 93.9 | 389.9 | 10.8 |
|  | *gad2* |  | 1401 | 320.73 | 1077.27 | 48(48) | 41 | 7 | 39 | 9 | 147 | 164.5 | 149.6 | 545.7 | 39.2 |
|  |  |  |  |  |  |  |  |  |  |  |  |  |  |  |  |

**Supplementary Table 5:** (cont.)

| **Population**  ***L. brevis*** | | **N** | **Total bases** | **Syn. sites** | **Nonsyn. sites** | **S(Eta)** | **Sing.** | **Par.** | **Syn. Pol.** | **Rep. Pol.** | **π (Pi) All Sites** | **Theta-W**  **All Sites**  **(**θ) | **π (JC)**  **All**  **Sites** | **π (JC) Syn. sites** | **π (JC)**  **Nonsyn. sites** |
| --- | --- | --- | --- | --- | --- | --- | --- | --- | --- | --- | --- | --- | --- | --- | --- |
| **Fermented**  **Beverage**  **Group** | *Operon* | 10 | 3516  (3771) | 807.95  (1059) | 2708.05 | 100(100) | 52 | 48 | 93 | 3 | 82.1 | 93.7 | 82.8 | 367.3  (289.8) | 3.5 |
|  | *gadR* |  | 591 | 135.67 | 455.33 | 14(14) | 6 | 8 | 14 | 0 | 75.2 | 83.7 | 75.8 | 339.5 | - |
|  | *gadC* |  | 1492 | 346.83 | 1141.17 | 47(47) | 27 | 20 | 44 | 2 | 90.9 | 111.4 | 91.7 | 379 | 6.6 |
|  | *gad1* |  | 1440 | 325.45 | 1111.55 | 36(36) | 18 | 18 | 35 | 1 | 81.5 | 88.4 | 82.1 | 367.5 | 1.8 |
|  | *gad2* |  | 1401 | 321.43 | 1076.57 | 43(43) | 23 | 20 | 34 | 9 | 99.1 | 108.5 | 100.1 | 364.8 | 23.8 |
| **Feces**  **Group** | *Operon* | 5 | 3534  (3785) | 811.03  (1062) | 2722.97 | 164(167) | 116 | 48 | 142 | 15 | 200.8 | 208 | 204.2 | 871.2  (698.7) | 21.7 |
|  | *gadR* |  | 591 | 135.43 | 455.7 | 13(13) | 8 | 5 | 12 | 1 | 104.9 | 105.6 | 105.9 | 445.6 | 8.8 |
|  | *gadC* |  | 1506 | 350.80 | 1155.20 | 95(98) | 68 | 27 | 90 | 8 | 293.5 | 302.8 | 301.9 | 1321.7 | 26.9 |
|  | *gad1* |  | 1440 | 324.80 | 1112.20 | 46(46) | 31 | 15 | 40 | 6 | 148.6 | 153.3 | 150.2 | 611.1 | 21.6 |
|  | *gad2* |  | 1401 | 321.60 | 1076.40 | 31(31) | 3 | 28 | 24 | 7 | 128.5 | 106.2 | 130.1 | 457.8 | 37.3 |
|  |  |  |  |  |  |  |  |  |  |  |  |  |  |  |  |

**Notes:** (N)Sample size show the number of sequences analyzed. Syn: Synonymous sites, Nonsyn: Nonsynonymous sites, S: Number of segregating sites, Eta: Number of mutations, Sing: Singleton variable sites, Par. : Parsimony informative sites, Syn. Pol. : Number of synonymous polymorphisms, Rep. Pol. : Number of replacement (nonsynonymous) polymorphisms, JC: Jukes-Cantor correction applied estimates. θ and π values represent percent sequence diversity, and for exact estimates, table values should be multiplied by 10-4. Total base count for operon includes only the coding regions. The total base count in the parenthesis includes coding and non-coding sites.

**Supplementary Table 6:** Population genetic summary statistics for nucleotide diversity of glutamate decarboxylase pathway genes among *Lactiplantibacillus plantarum* population.

| **Population**  ***L. plantarum*** | | **N** | **Total bases** | **Syn. sites** | **Nonsyn. sites** | **S(Eta)** | **Sing.** | **Par.** | **Syn. Pol.** | **Rep. Pol.** | | **π (Pi) All Sites** | **Theta-W**  **All Sites**  **(**θ) | **π (JC)**  **All**  **Sites** | **π (JC) Syn. sites** | **π (JC) Nonsyn. sites** |
| --- | --- | --- | --- | --- | --- | --- | --- | --- | --- | --- | --- | --- | --- | --- | --- | --- |
| **All Samples** | | 88 |  | | | | | | | | | | | | | |
|  | *gadB* |  | 1410 | 323.35 | 1077.65 | 121 | 39 | 82 | 82 | 38 | 85.3 | | 170.7 | 86.2 | 247.4 | 37.2 |
|  | *yjeM* |  | 1485 | 351.76 | 1130.24 | 65 | 30 | 35 | 51 | 14 | 50.4 | | 86.7 | 50.6 | 178.2 | 11.7 |
|  |  |  |  |  |  |  |  |  |  |  |  | |  |  |  |  |
| **Kimchi Group** | | 28 |  | | | | | | | | | | | | | |
|  | *gadB* |  | 1410 | 323.31 | 1077.69 | 88 | 48 | 40 | 67 | 20 | 74.8 | | 161.1 | 76 | 252.4 | 26.1 |
|  | *yjeM* |  | 1485 | 351.43 | 1130.57 | 39 | 19 | 20 | 32 | 7 | 51.8 | | 67.5 | 52 | 183.3 | 12.1 |
|  |  |  |  |  |  |  |  |  |  |  |  | |  |  |  |  |
| **Unspecified Fermented Food Group** | | 8 |  | | | | | | | | | | | | | |
|  | *gadB* |  | 1410 | 324.42 | 1082.58 | 32 | 22 | 10 | 17 | 14 | 70.2 | | 87.5 | 70.7 | 174.2 | 38.1 |
|  | *yjeM* |  | 1485 | 351.71 | 1130.29 | 15 | 6 | 9 | 12 | 3 | 37.8 | | 39 | 37.9 | 134.7 | 8.2 |
|  |  |  |  |  |  |  |  |  |  |  |  | |  |  |  |  |
| **Raw Foods** | | 4 |  | | | | | | | | | | | | | |
|  | *gadB* |  | 1410 | 324.17 | 1082.83 | 9 | 9 | 0 | 4 | 4 | 31.9 | | 34.8 | 32 | 62.1 | 18.5 |
|  | *yjeM* |  | 1485 | 351.71 | 1130.29 | 11 | 9 | 2 | 10 | 1 | 39.3 | | 40.4 | 39.4 | 153.5 | 4.4 |
|  |  |  |  |  |  |  |  |  |  |  |  | |  |  |  |  |
| **Kefir Group** | | 9 |  | | | | | | | | | | | | | |
|  | *gadB* |  | 1410 | 324.02 | 1082.98 | 38 | 14 | 24 | 26 | 11 | 111.9 | | 99.2 | 113.1 | 327.7 | 47.4 |
|  | *yjeM* |  | 1485 | 352.24 | 1129.76 | 12 | 2 | 10 | 8 | 4 | 29.2 | | 29.7 | 29.3 | 89.4 | 10.8 |
|  |  |  |  |  |  |  |  |  |  |  |  | |  |  |  |  |

**Supplementary Table 6:** (cont.)

| **Population**  ***L. plantarum*** | | **N** | **Total bases** | **Syn. sites** | **Nonsyn. sites** | **S(Eta)** | **Sing.** | **Par.** | **Syn. Pol.** | **Rep. Pol.** | **π (Pi) All Sites** | **Theta-W**  **All Sites**  **(θ)** | **π (JC)**  **All**  **Sites** | **π (JC) Syn. sites** | **π (JC) Nonsyn. sites** |
| --- | --- | --- | --- | --- | --- | --- | --- | --- | --- | --- | --- | --- | --- | --- | --- |
| **Cheese Group** | | 10 |  | | | | | | | | | | | | |
|  | *gadB* |  | 1410 | 323.30 | 1077.70 | 62 | 38 | 24 | 38 | 23 | 136 | 156 | 137.8 | 382.8 | 62.8 |
|  | *yjeM* |  | 1485 | 351.77 | 1130.23 | 22 | 8 | 14 | 15 | 7 | 51.9 | 52.4 | 52.2 | 168.7 | 16.5 |
|  |  |  |  |  |  |  |  |  |  |  |  |  |  |  |  |
| **Beverage Group** | | 6 |  | | | | | | | | | | | | |
|  | *gadB* |  | 1410 | 323.64 | 1077.36 | 24 | 14 | 10 | 17 | 6 | 71.7 | 74.9 | 72.1 | 219 | 24.2 |
|  | *yjeM* |  | 1485 | 352.08 | 1129.92 | 14 | 5 | 9 | 12 | 2 | 43.5 | 41.3 | 43.7 | 161.6 | 7.7 |
|  |  |  |  |  |  |  |  |  |  |  |  |  |  |  |  |
| **Meat Group** | | 6 |  | | | | | | | | | | | | |
|  | *gadB* |  | 1410 | 323.56 | 1077.44 | 25 | 22 | 3 | 14 | 11 | 63.6 | 78 | 64.2 | 154.7 | 37.9 |
|  | *yjeM* |  | 1485 | 351.83 | 1130.17 | 21 | 11 | 10 | 18 | 3 | 61.1 | 61.9 | 61.4 | 228 | 10.6 |
|  |  |  |  |  |  |  |  |  |  |  |  |  |  |  |  |
| **Feces Group** | | 5 |  | | | | | | | | | | | | |
|  | *gadB* |  | 1410 | 323.87 | 1077.13 | 12 | 11 | 1 | 7 | 4 | 35.6 | 41 | 35.7 | 87 | 16.7 |
|  | *yjeM* |  | 1485 | 351.73 | 1130.27 | 19 | 14 | 5 | 16 | 3 | 57.9 | 61.4 | 58.2 | 214 | 10.6 |
|  |  |  |  |  |  |  |  |  |  |  |  |  |  |  |  |
| **Other Milk-Based** | | 12 |  | | | | | | | | | | | | |
| **Fermented**  **Products** | *gadB* |  | 1410 | 323.31 | 1077.69 | 35 | 8 | 27 | 20 | 14 | 80.3 | 82.5 | 80.9 | 217.9 | 35.8 |
|  | *yjeM* |  | 1485 | 351.94 | 1130.06 | 22 | 11 | 11 | 18 | 4 | 47.2 | 49.1 | 47.4 | 168.7 | 10.3 |
|  |  |  |  |  |  |  |  |  |  |  |  |  |  |  |  |

**Notes:** (N)Sample size show the number sequences analyzed. Syn: Synonymous sites, Nonsyn: Nonsynonymous sites, S: Number of segregating sites, Eta: Number of mutations,Sing:Singleton variable sites,Par. :Parsimony informative sites, Syn. Pol. : Number of synonymous polymorphisms, Rep. Pol. : Number of replacement (nonsynonymous) polymorphisms, JC: Jukes-Cantor correction applied estimates. θ and π values represent percent sequence diversity and for exact estimates table values should be multiplied by 10^-4^.

**Suplementary Table 7:** Neutrality tests summary statistics for glutamate decarboxylase pathway genes among *Levilactobacillus brevis* populations.

| **Population** | TD | TD - Cod. | TD – Syn. | TD – Nonsyn. | TD - Silent | Fu-Li’s *D** | Fu-Li’s *F** |
| --- | --- | --- | --- | --- | --- | --- | --- |
| ***L. brevis*** |  |  |  |  |  |  |  |
| **All Samples (N=30)** | | | |  |  |  |  |
| *Operon* | -0.89 | -0.84 | -0.60 | -2.24** | -0.67 | -1.74 | -1.72 |
| *gadR* | -0.37 | -0.37 | 0.06 | -1.75# | 0.06 | -0.40 | -0.45 |
| *gadC* | -1.26 | -1.27 | -1.11 | -2.16* | -1.11 | -2.40# | -2.39# |
| *gad1* | -0.23 | -0.23 | 0.15 | -1.81* | 0.15 | -0.59 | -0.56 |
| *gad2* | -0.42 | -0.42 | -0.25 | -0.85 | -0.25 | -1.21 | -1.12 |
| **Fermented Vegetable Group (N=6)** | | | |  |  |  |  |
| *Operon* | -0.04 | -0.09 | -0.06 | -0.35 | 0.00 | -0.06 | -0.06 |
| *gadR* | 0.24 | 0.24 | 0.24 | n.a. | 0.24 | 0.34 | 0.35 |
| *gadC* | 0.08 | 0.08 | 0.12 | -0.19 | 0.12 | -0.13 | -0.09 |
| *gad1* | -0.46 | -0.46 | -0.44 | -0.45 | -0.44 | -0.36 | -0.41 |
| *gad2* | -0.39 | -0.39 | -0.25 | -0.74 | -0.25 | -0.29 | -0.34 |
| **Sourdough Group (N=4)** | | | |  |  |  |  |
| *Operon* | -0.13 | -0.09 | 0.00 | -0.83 | -0.05 | -0.13 | -0.14 |
| *gadR* | -0.64 | -0.64 | -0.56 | -0.78 | -0.56 | -0.64 | -0.66 |
| *gadC* | -0.14 | -0.14 | -0.10 | -0.71 | -0.10 | -0.14 | -0.15 |
| *gad1* | 0.19 | 0.19 | 0.29 | -0.75 | 0.29 | 0.19 | 0.20 |
| *gad2* | -0.25 | -0.25 | -0.18 | -0.45 | -0.18 | -0.25 | -0.26 |
| **Fermented Dairy Product (N=5)** | | | |  |  |  |  |
| *Operon* | -0.24 | -0.27 | -0.21 | -1.12 | -0.17 | -0.24 | -0.26 |
| *gadR* | 0.31 | 0.31 | 0.52 | -0.97 | 0.52 | 0.31 | 0.33 |
| *gadC* | -0.32 | -0.32 | -0.32 | n.a. | -0.32 | -0.32 | -0.35 |
| *gad1* | -0.52 | -0.52 | -0.44 | -1.05 | -0.44 | -0.52 | -0.56 |
| *gad2* | -0.80 | -0.80 | -0.93 | -0.20 | -0.93 | -0.80 | -0.86 |
|  |  |  |  |  |  |  |  |
| **Fermented Beverage Group (N=10)** | | | |  |  |  |  |
| *Operon* | -0.62 | -0.64 | -0.65 | -0.36 | -0.60 | -0.54 | -0.63 |
| *gadR* | -0.47 | -0.47 | -0.47 | n.a. | -0.47 | -0.14 | -0.25 |
| *gadC* | -0.90 | -0.87 | -0.92 | 0.22 | -0.92 | -0.75 | -0.89 |
| *gad1* | -0.38 | -0.38 | -0.33 | -1.11 | -0.33 | -0.44 | -0.48 |
| *gad2* | -0.42 | -0.42 | -0.28 | -0.86 | -0.28 | -0.59 | -0.62 |
| **Feces Group (N=5)** | | | |  |  |  |  |
| *Operon* | -0.40 | -0.36 | -0.25 | -1.32*** | -0.30 | -0.36 | -0.40 |
| *gadR* | -0.05 | -0.05 | 0.05 | -0.82 | 0.05 | -0.05 | -0.05 |
| *gadC* | -0.46 | -0.46 | -0.37 | -1.36*** | -0.37 | -0.39 | -0.44 |
| *gad1* | -0.23 | -0.23 | -0.08 | -1.15 | -0.08 | -0.23 | -0.25 |
| *gad2* | 1.57 | 1.57 | 1.60 | 1.33 | 1.60 | 1.57 | 1.69 |
|  |  |  |  |  |  |  |  |

**Notes:** TD: Tajima’s *D* test, Cod.: Coding sites, Syn: Synonymous sites,

Nonsyn: Nonsynonymous sites, Silent: Silent sites.

* represent P < 0.05, ** represent P < 0.01, *** represent P < 0.001.

# represent 0.10 > P > 0.0 ,Operon*: gadR+gadC+gad1,* N: sample size

**Supplementary Table 8:** Neutrality tests summary statistics for glutamate decarboxylase pathway genes among *Lactiplantibacillus plantarum* populations.

| **Population** | **TD** | | **TD - Cod.** | | **TD – Syn.** | | **TD – Nonsyn.** | | **TD - Silent** | | | **Fu-Li’s *D**** | | **Fu-Li’s *F**** |
| --- | --- | --- | --- | --- | --- | --- | --- | --- | --- | --- | --- | --- | --- | --- |
| ***L. plantarum*** |  |  |  |  |  |  |  |  |  |  |  |  |  |  |
| **All Samples (N=88)** | | | | | | | | | | | | | | |
| *gadB* | -1.68# | | -1.71# | | -1.75# | | -1.48 | | -1.70# | | | -1.46 | | -1.86 |
| *YjeM* | -1.37 | | -1.37 | | -1.26 | | -1.46 | | -1.26 | | | -2.91* | | -2.74* |
| **Kimchi Group** **(N=28)** | | | | | | | | | | | | | | |
| *gadB* | -2.06* | | -2.05* | | -2.13* | | -1.60# | | -2.14* | | | -1.96# | | -2.36# |
| *YjeM* | -0.87 | | -0.87 | | -0.85 | | -0.73 | | -0.85 | | | -1.46 | | -1.50 |
| **Unspecified Fermented Food Group (N=8)** | | | | | | | | | | | | | | |
| *gadB* | -1.05 | | -1.03 | | -0.83 | | -1.19 | | -0.89 | | | -0.91 | | -1.05 |
| *YjeM* | -0.16 | | -0.16 | | 0.05 | | -0.81 | | 0.05 | | | 0.14 | | 0.08 |
| **Raw Foods (N=4)** | | | | | | | | | | | | | | |
| *gadB* | -0.83 | | -0.82 | | -0.78 | | -0.78 | | -0.80 | | | -0.83 | | -0.82 |
| *YjeM* | -0.28 | | -0.28 | | -0.22 | | -0.61 | | -0.22 | | | -0.28 | | -0.28 |
| **Kefir Group (N=9)** | | | | | | | | | | | | | | |
| *gadB* | 0.65 | | 0.66 | | 0.37 | | 1.24 | | 0.37 | | | 0.18 | | 0.33 |
| *YjeM* | -0.09 | | -0.09 | | 0.26 | | -0.69 | | 0.26 | | | 0.90 | | 0.74 |
| **Cheese Group (N=10)** | | | | | | | | | | | | | | |
| *gadB* | | -0.63 | | -0.68 | | -0.58 | | -0.81 | | -0.50 | -0.92 | | -0.96 | |
| *YjeM* | | -0.04 | | -0.04 | | 0.47 | | -1.04 | | 0.47 | 0.12 | | 0.09 | |
| **Beverage Group** **(N=6)** | | | | | | | | | | | | | | |
| *gadB* | | -0.27 | | -0.34 | | -0.43 | | -0.06 | | -0.33 | -0.19 | | -0.22 | |
| *YjeM* | | 0.34 | | 0.34 | | 0.40 | | -0.05 | | 0.40 | 0.53 | | 0.53 | |
| **Meat Group (N=6)** | | | | | | | | | | | | | | |
| *gadB* | | -1.16 | | -1.16 | | -1.27 | | -0.94 | | -1.27 | -1.15 | | -1.26 | |
| *YjeM* | | -0.09 | | -0.09 | | -0.01 | | -0.45 | | -0.01 | 0.01 | | -0.02 | |
| **Feces Group (N=5)** | | | | | | | | | | | | | | |
| *gadB* | | -0.95 | | -0.93 | | -1.16 | | -0.41 | | -1.17 | -0.95 | | -1.01 | |
| *YjeM* | | -0.42 | | -0.42 | | -0.27 | | -1.05 | | -0.27 | -0.42 | | -0.45 | |
| **Other Milk Based Fermented Products (N=12)** | | | | | | | | | | | | | | |
| *gadB* | | -0.12 | | -0.21 | | 0.18 | | -0.72 | | 0.30 | 0.59 | | 0.46 | |
| *YjeM* | | -0.16 | | -0.16 | | -0.08 | | -0.42 | | -0.08 | -0.60 | | -0.55 | |
|  | |  | |  | |  | |  | |  |  | |  | |

**Notes:** TD: Tajima’s *D* test, Cod.: Coding sites, Syn: Synonymous sites,

Nonsyn: Nonsynonymous sites, Silent: Silent sites., N: Sample size.

* represent P < 0.05, # represent 0.10 > P > 0.05 .

**Supplementary Table 9-A:** *Levilactobacillus brevis* GAD pathway (*gad1-gadC* and *gad2*) Fu-Li’s D, Fu-Li’s F, and other tests with outgroup species.

|  | **Total**  **Sites** | **S** | **Eta** | **Eta**  **(e)** | **Eta**  **(s)** | **FL-D** | **FL-F** | **FW-Hn** | **ZE** | **AY** |
| --- | --- | --- | --- | --- | --- | --- | --- | --- | --- | --- |
| *Levilactobacillus brevis gad1* gene with; | | | | | | | | | | |
| ***L. sakei*** | | |  |  |  |  |  |  |  |  |
|  | 1437 | 47 | 47 | 11 | 11 | 0.40 | 0.42 | 0.92 | -0.74 | 0.06 |
| ***L. zymae*** | | |  |  |  |  |  |  |  |  |
|  | 1437 | 47 | 47 | 11 | 11 | 0.40 | 0.42 | 0.92 | -0.74 | 0.06 |
| ***L. cerevisiae*** | | |  |  |  |  |  |  |  |  |
|  | 1437 | 38 | 38 | 8 | 11 | 0.55 | 0.48 | -0.94 | 2.14 | -0.22 |
| ***L .angrenensis*** | | |  |  |  |  |  |  |  |  |
|  | 1437 | 37 | 37 | 8 | 10 | 0.51 | 0.45 | -0.88 | 2.24 | -0.22 |
| ***L. spicheri*** | | |  |  |  |  |  |  |  |  |
|  | 1407 | 36 | 36 | 5 | 9 | 1.03 | 0.93 | -1.43 | 2.84 | -0.39 |
| *Levilactobacillus brevis gadC* gene with; | | | | | | | | | | |
| ***L. sakei*** | | |  |  |  |  |  |  |  |  |
|  | 1503 | 59 | 59 | 18 | 18 | -0.10 | -0.27 | 1.09 | -1.58 | -0.59 |
| ***L. zymae*** | | |  |  |  |  |  |  |  |  |
|  | 1503 | 59 | 59 | 18 | 18 | -0.10 | -0.27 | 1.09 | -1.58 | -0.59 |
| ***L. cerevisiae*** | | |  |  |  |  |  |  |  |  |
|  | 1503 | 48 | 48 | 10 | 14 | 0.57 | 0.30 | -2.52 | 3.40 | -1.08 |
| ***L. angrenensis*** | | |  |  |  |  |  |  |  |  |
|  | 1503 | 46 | 46 | 9 | 13 | 0.66 | 0.39 | -1.95 | 3.16 | -1.13 |
| ***L. spicheri*** | | |  |  |  |  |  |  |  |  |
|  | 1503 | 48 | 48 | 10 | 14 | 0.58 | 0.31 | -2.46 | 3.37 | -1.08 |
| *Levilactobacillus brevis gad2* gene with; | | | | | | | | | | |
| ***L. herbarum*** | | |  |  |  |  |  |  |  |  |
|  | 1398 | 50 | 51 | 13 | 24 | 0.25 | -0.01 | -3.38 | 4.54 | -1.00 |
| ***L. paraplantarum*** | | |  |  |  |  |  |  |  |  |
|  | 1398 | 48 | 49 | 12 | 24 | 0.32 | -0.03 | -3.34 | 4.40 | -1.05 |
| ***L. argentoratensis*** | | |  |  |  |  |  |  |  |  |
|  | 1398 | 47 | 48 | 10 | 22 | 0.57 | 0.29 | -3.13 | 4.63 | -1.14 |
| ***L. plantarum*** | | |  |  |  |  |  |  |  |  |
|  | 1398 | 47 | 48 | 10 | 22 | 0.57 | 0.29 | -3.22 | 4.70 | -1.14 |
| ***C. futsaii*** | | |  |  |  |  |  |  |  |  |
|  | 1398 | 47 | 48 | 10 | 22 | 0.57 | 0.29 | -3.22 | 4.70 | -1.14 |
|  |  |  |  |  |  |  |  |  |  |  |

**Note:** S: Number of segregating sites, Eta: Total number of mutations, Eta(e): Total number of mutations in external branches, Eta(s): Total number of singleton mutations, FL-D: Fu and Li's D test statistic, FL-F: Fu and Li's F test statistic, FW-Hn: Normalized Fay and Wu's Hn test statistic, ZE: Zeng E test statistic, AY: Achaz Y test statistic.

**Supplementary Table 9-B:** *Lactiplantibacillus plantarum* GAD pathway (*gadB* and *yjeM*) Fu-Li’s D, Fu-Li’s F, and other tests with outgroup species.

|  | **Total**  **Sites** | **S** | **Eta** | **Eta**  **(e)** | **Eta**  **(s)** | **FL-D** | **FL-F** | **FW-Hn** | **ZE** | **AY** |
| --- | --- | --- | --- | --- | --- | --- | --- | --- | --- | --- |
| *Lactiplantibacillus plantarum gadB* gene with; | | | | | | | | | | |
| ***L. brevis-gad2*** | | |  |  |  |  |  |  |  |  |
|  | 1398 | 88 | 88 | 18 | 26 | -0.08 | -0.91 | -4.44 | 5.06 | -2.03 |
| ***L. herbarum*** | | |  |  |  |  |  |  |  |  |
|  | 1401 | 101 | 101 | 17 | 33 | -0.39 | -0.64 | -4.99 | 4.38 | -2.04 |
| ***L. paraplantarum*** | | |  |  |  |  |  |  |  |  |
|  | 1401 | 103 | 103 | 23 | 32 | -0.33 | -1.10 | -3.98 | 3.52 | -1.96 |
| ***L. argentoratensis*** | | |  |  |  |  |  |  |  |  |
|  | 1401 | 120 | 120 | 33 | 39 | -1.04 | -1.62 | -2.62 | 0.84 | -1.79 |
| ***C. futsaii*** | | |  |  |  |  |  |  |  |  |
|  | 1401 | 120 | 120 | 39 | 39 | -1.72 | -2.07# | 0.60 | -2.09 | -1.67 |
| *Lactiplantibacillus plantarum YjeM* gene with; | | | | | | | | | | |
| ***L. paraplantarum*** | | |  |  |  |  |  |  |  |  |
|  | 1482 | 64 | 64 | 21 | 30 | -1.60 | -1.87 | -3.19 | 1.6 | -1.23 |
| ***L. argentoratensis*** | | |  |  |  |  |  |  |  |  |
|  | 1482 | 65 | 65 | 28 | 30 | -2.87* | -2.70* | -0.10 | -1.13 | -0.83 |
|  |  |  |  |  |  |  |  |  |  |  |

**Note:** S: Number of segregating sites, Eta: Total number of mutations, Eta(e): Total number of mutations in external branches, Eta(s): Total number of singleton mutations, FL-D: Fu and Li's D test statistic, FL-F: Fu and Li's F test statistic, FW-Hn: Normalized Fay and Wu's Hn test statistic, ZE: Zeng E test statistic, AY: Achaz Y test statistic.

**Supplementary Table 10-A:** Enzyme domain information for *L. brevis gad1*/gad2 and *L.* *plantarum gadB*.

| **Gene** | **N-terminal Domain** | **PLP-binding Domain** | **Small (C-terminal)**  **Domain** |
| --- | --- | --- | --- |
| *gad1 (479 aa)* | 1-59 aa  (1-177 nt) | 60-375 aa  (180-1125 nt) | 375-479 aa  (1125-1437 nt) |
| *gad2 (468 aa)* | 1-57 aa  (1-171 nt) | 58-364 aa  (174-1092 nt) | 364-468 aa  (1092-1404 nt) |
| *gadB (469 aa)* | 1-57 aa  (1-171 nt) | 58-365 aa  (174-1095 nt) | 365-469 aa  (1095-1407 nt) |

**Supplementary Table 10-B:** Nucleotide diversity and TD (Tajima’s D test) calculations of *L. brevis (gad1/gad2)* and *L. plantarum (gadB) gad* genes enzyme domains.

| **N-terminal Domain** | π-Total | π-Nonsyn | TD | TD-Nonsyn |
| --- | --- | --- | --- | --- |
| *gad1* | 0.0055 | 0.0014 | -1.57 | -1.73 |
| *gad2* | 0.0210 | 0.0094 | -0.30 | -0.14 |
| *gadB* | 0.0027 | 0.0009 | -1.61 | -1.74 |
| **PLP-binding Domain** | π-Total | π-Nonsyn | TD | TD-Nonsyn |
| *gad1* | 0.0113 | 0.0007 | 0.24 | -1.28 |
| *gad2* | 0.0130 | 0.0024 | -0.62 | -1.42 |
| *gadB* | 0.0093 | 0.0042 | -1.66 | -1.50 |
| **Small (C-terminal)**  **Domain** | π-Total | π-Nonsyn | TD | TD-Nonsyn |
| *gad1* | 0.0087 | 0.0015 | -0.55 | -1.20 |
| *gad2* | 0.0065 | 0.0021 | 1.01 | 1.63 |
| *gadB* | 0.0082 | 0.0037 | -1.52 | -0.19 |

**Note:** π-Total: Overall nucleotide diversity, π-Nonsyn: Nonsynonymous sites nucleotide diversity, TD: Tajima’s D test, TD-Nonsyn: Nonsynonymous sites Tajima’s D.

**Supplementary Table 11-A1:** Replacement(Nonsynonymous) sites in the *L. brevis gadR* gene and their corresponding amino acids on the protein. The nucleotides changed in the triple codon structure, and the new amino acids formed by the change are shown in red.

| *L. brevis*  *gadR*  591 nt | **Isolation Group** | **Num. RP** | **Nucleotide Position of RP** | |
| --- | --- | --- | --- | --- |
|  | Fermented Vegetable | 0 |  | |
|  | Sourdough | 4 | 164-210-241-277 | |
|  | Fermented Dairy Products | 2 | 277-571 | |
|  | Fermented Beverage | 0 |  | |
|  | Feces | 1 | 277 | |
|  |  |  |  | |
| **Amino acid Changes on *L. brevis gadR*** | | | | |
| **N.No** | **Nucleotide Changes** | **Codons** | **Amino acid Change** | **R.No** |
| 164. nt | A change to G | CAG/CGG | Glutamine/Arginine | 55. aa |
| 210. nt | C change to A | GAC/GAA | Aspartic acid/Glutamic acid | 70. aa |
| 241 .nt | C change to T | CGC/TGC | Arginine/Cysteine | 81. aa |
| 277. nt | G change to A | GTC/ATC | Valine/Isoleucine | 93. aa |
| 571. nt | C change to T | CAA/TAA | Glutamine/Stop | 191. aa |
|  |  |  |  |  |

**Note:** Num. RP: Number of replacement polymorphisms,N.No:Nucleotide number, R.No:Residue number.

**Supplementary Table 11-A2:** Biochemical characteristics of amino acid changes obseved in *L. brevis gadR* gene.

| **R.No** | **Amino Acid**  **1-Letter Code** | | **Class** | **Charge Features** | | | **Polarity** | | **Interaction Modes** | **B** | **pI** | **H** | **Sing.** | **PROVEAN** |
| --- | --- | --- | --- | --- | --- | --- | --- | --- | --- | --- | --- | --- | --- | --- |
|  |  |  |  | **Positive** | **Negative** | **Neutral** | **Polar** | **Nonpolar** |  |  |  |  |  |  |
| **55** | Glutamine | Q | Amide | - | - | YES | YES | - | H-bonds, van der Waals | 5 | 5.7 | 0.430 | YES | Neutral |
|  | Arginine | R | Basic | YES | - | - | YES | - | Ionic, H-bonds, van der Waals | 7 | 10.8 | 0.000 |  |  |
| **70** | Aspartic acid | D | Acid | - | YES | - | YES | - | Ionic, H-bonds, van der Waals | 4 | 3.0 | 0.417 | YES | Neutral |
|  | Glutamic acid | E | Acid | - | YES | - | YES | - | Ionic, H-bonds, van der Waals | 4 | 3.2 | 0.458 |  |  |
| **81** | Arginine | R | Basic | YES | - | - | YES | - | Ionic, H-bonds, van der Waals | 7 | 10.8 | 0.000 | YES | Deleterious |
|  | Cysteine | C | Sulfuric | - | - | YES | YES | - | Covalent disulfide bonds, van der Waals | 0 | 5.0 | 0.721 |  |  |
| **93** | Valine | V | Aliphatic | - | - | YES | - | YES | van der Waals | 0 | 6.0 | 0.923 | NO | Neutral |
|  | Isoleucine | I | Aliphatic | - | - | YES | - | YES | van der Waals | 0 | 6.0 | 1,000 |  |  |
| **191** | Glutamine | Q | Amide | - | - | YES | YES | - | H-bonds, van der Waals | 5 | 5.7 | 0.430 | YES | - |
|  | Stop | * | - | - | - | - | - | - | - | - | - | - |  |  |

**Note:** R.No:Residue number, B:Potential side chain H-bonds, pI:Isoelectric point, H:Hydrophobicity, Sing.: Singleton

**Supplementary Table 11-B1:** Replacement(Nonsynonymous) sites in the *L. brevis gadC* gene and their corresponding amino acids on the protein. The nucleotides changed in the triple codon structure, and the new amino acids formed by the change are shown in red.

| *L. brevis*  *gadC*  1506 nt | **Isolation Group** | **Num. RP** | **Nucleotide Position of RP** | |
| --- | --- | --- | --- | --- |
|  | Fermented Vegetable | 3 | 99-1028-1099 | |
|  | Sourdough | 2 | 80-1313 | |
|  | Fermented Dairy Products | 0 |  | |
|  | Fermented Beverage | 3 | 278-512-1099 | |
|  | Feces | 8 | 20-421-739-797-865-821-1105-1105 | |
|  |  |  |  | |
| **Amino acid Changes on *L. brevis gadC*** | | | | |
| **N.No** | **Nucleotide Changes** | **Codons** | **Amino acid Change** | **R.No** |
| 20. nt | A change to G | GAA/GGA | Glutamic acid/Glycine | 7. aa |
| 80. nt | C change to T | ACG/ATG | Threonine/Methionine | 27. aa |
| 278. nt | G change to T | GGT/GTT | Glycine/Valine | 93. aa |
| 421. nt | A change to G | ATC/GTT | Isoleucine/Valine | 141. aa |
| 512. nt | T change to C | ATC/ACC | Isoleucine/Threonine | 171. aa |
| 739. nt | G change to A | GTT/ATT | Valine/Isoleucine | 247. aa |
| 797. nt | A change to G | AAT/AGT | Asparagine/Serine | 266. aa |
| 865. nt | G change to A | GGC/AGC | Glycine/Serine | 289. aa |
| 871. nt | G change to A | GTC/ATT | Valine/Isoleucine | 291. aa |
| 995. nt | G change to A | CGC/CAC | Arginine/Histidine | 332. aa |
| 1028. nt | C change to T | ACC/ATC | Threonine/Isoleucine | 343. aa |
| 1099. nt | A change to G | AAC/GAC | Asparagine/Aspartic acid | 367. aa |
| 1105. nt | G change to T | GCG/TCG | Alanine/Serine | 369. aa |
| 1105. nt | G change to A | GCG/ACG | Alanine/Threonine | 369. aa |
| 1313. nt | T change to C | TTC/TCC | Phenylalanine/Serine | 438. aa |
|  |  |  |  |  |

**Note:** Num. RP: Number of replacement polymorphisms, N.No:Nucleotide number, R.No:Residue number.

**Supplementary Table 11-B2:** Biochemical characteristics of amino acid changes obseved in *L. brevis gadC* gene.

| **R.No** | **Amino Acid**  **1-Letter Code** | | **Class** | **Charge Features** | | | **Polarity** | | **Interaction Modes** | **B** | **pI** | **H** | **Sing.** | **PROVEAN** |
| --- | --- | --- | --- | --- | --- | --- | --- | --- | --- | --- | --- | --- | --- | --- |
|  |  |  |  | **Positive** | **Negative** | **Neutral** | **Polar** | **Nonpolar** |  |  |  |  |  |  |
| **7** | Glutamic Acid | E | Acid | - | YES | - | YES | - | Ionic, H-bonds, van der Waals | 4 | 3.2 | 0.458 | YES | Neutral |
|  | Glycine | G | Aliphatic | - | - | YES | - | YES | van der Waals | 0 | 6.0 | 0.770 |  |  |
| **27** | Threonine | T | Hydroxylic | - | - | YES | YES | - | H-bonds, van der Waals | 3 | 5.6 | 0.634 | YES | Deleterious |
|  | Methionine | M | Sulfuric | - | - | YES | - | YES | van der Waals | 0 | 5.7 | 0.811 |  |  |
| **93** | Glycine | G | Aliphatic | - | - | YES | - | YES | van der Waals | 0 | 6.0 | 0.770 | YES | Deleterious |
|  | Valine | V | Aliphatic | - | - | YES | - | YES | van der Waals | 0 | 6.0 | 0.923 |  |  |
| **141** | Isoleucine | I | Aliphatic | - | - | YES | - | YES | van der Waals | 0 | 6.0 | 1,000 | YES | Neutral |
|  | Valine | V | Aliphatic | - | - | YES | - | YES | van der Waals | 0 | 6.0 | 0.923 |  |  |
| **171** | Isoleucine | I | Aliphatic | - | - | YES | - | YES | van der Waals | 0 | 6.0 | 1,000 | NO | Deleterious |
|  | Threonine | T | Hydroxylic | - | - | YES | YES | - | H-bonds, van der Waals | 3 | 5.6 | 0.634 |  |  |
| **247** | Valine | V | Aliphatic | - | - | YES | - | YES | van der Waals | 0 | 6.0 | 0.923 | YES | Neutral |
|  | Isoleucine | I | Aliphatic | - | - | YES | - | YES | van der Waals | 0 | 6.0 | 1,000 |  |  |
| **266** | Asparagine | N | Amide | - | - | YES | YES | - | H-bonds, van der Waals | 5 | 5.4 | 0.448 | YES | Neutral |
|  | Serine | S | Hydroxylic | - | - | YES | YES | - | H-bonds, van der Waals | 3 | 5.7 | 0.601 |  |  |
| **289** | Glycine | G | Aliphatic | - | - | YES | - | YES | van der Waals | 0 | 6.0 | 0.770 | YES | Neutral |
|  | Serine | S | Hydroxylic | - | - | YES | YES | - | H-bonds, van der Waals | 3 | 5.7 | 0.601 |  |  |
| **291** | Valine | V | Aliphatic | - | - | YES | - | YES | van der Waals | 0 | 6.0 | 0.923 | YES | Neutral |
|  | Isoleucine | I | Aliphatic | - | - | YES | - | YES | van der Waals | 0 | 6.0 | 1,000 |  |  |
| **332** | Arginine | R | Basic | YES | - | - | YES | - | Ionic, H-bonds, van der Waals | 7 | 10.8 | 0.000 | YES | Neutral |
|  | Histidine | H | Basic aromatic | YES | - | - | YES | - | Ionic, H-bonds, aromatic stacking, van der Waals | 3 | 7.6 | 0.548 |  |  |

(cont. on next page)

**Supplementary Table 11-B2:** (cont.)

|  | **Amino Acid**  **1-Letter Code** | | **Class** | **Charge Features** | | | **Polarity** | | **Interaction Modes** | **B** | **pI** | **H** | **Sing.** | **PROVEAN** |
| --- | --- | --- | --- | --- | --- | --- | --- | --- | --- | --- | --- | --- | --- | --- |
|  |  |  |  | **Positive** | **Negative** | **Neutral** | **Polar** | **Nonpolar** |  |  |  |  |  |  |
| **343** | Threonine | T | Hydroxylic | - | - | YES | YES | - | H-bonds, van der Waals | 3 | 5.6 | 0.634 | YES | Neutral |
|  | Isoleucine | I | Aliphatic | - | - | YES | - | YES | van der Waals | 0 | 6.0 | 1,000 |  |  |
| **367** | Asparagine | N | Amide | - | - | YES | YES | - | H-bonds, van der Waals | 5 | 5.4 | 0.448 | NO | Neutral |
|  | Aspartic acid | D | Acid | - | YES | - | YES | - | Ionic, H-bonds, van der Waals | 4 | 3.0 | 0.417 |  |  |
| **369** | Alanine | A | Aliphatic | - | - | YES | - | YES | van der Waals | 0 | 6.0 | 0.806 | YES | Neutral |
|  | Serine | S | Hydroxylic | - | - | YES | YES | - | H-bonds, van der Waals | 3 | 5.7 | 0.601 |  |  |
| **369** | Alanine | A | Aliphatic | - | - | YES | - | YES | van der Waals | 0 | 6.0 | 0.806 | YES | Neutral |
|  | Threonine | T | Hydroxylic | - | - | YES | YES | - | H-bonds, van der Waals | 3 | 5.6 | 0.634 |  |  |
| **438** | Phenylalanine | F | Aromatic | - | - | YES | - | YES | Aromatic stacking, van der Waals | 0 | 5.5 | 0.951 | YES | Deleterious |
|  | Serine | S | Hydroxylic | - | - | YES | YES | - | H-bonds, van der Waals | 3 | 5.7 | 0.601 |  |  |

**Note:** R.No:Residue number, B:Potential side chain H-bonds, pI:Isoelectric point, H:Hydrophobicity, Sing.: Singleton

**Supplementary Table 11-C1:** Replacement(Nonsynonymous) sites in the *L. brevis gad1* gene and their corresponding amino acids on the protein. The nucleotides changed in the triple codon structure, and the new amino acids formed by the change are shown in red.

| *L. brevis*  *gad1*  1440 nt | **Isolation Group** | **Num. RP** | **Nucleotide position of RP** | |
| --- | --- | --- | --- | --- |
|  | Fermented Vegetable | 3 | 26-719-1414 | |
|  | Sourdough | 3 | 119-1016-1413 | |
|  | Fermented Dairy Products | 3 | 698-1016-1413 | |
|  | Fermented Beverage | 1 | 1413 | |
|  | Feces | 6 | 112-701-719-1016-1219-1413 | |
|  |  |  |  | |
| **Amino acid Changes on *L. brevis gad1*** | | | | |
| **N.No** | **Nucleotide Changes** | **Codons** | **Amino acid Change** | **R.No** |
| 26. nt | A change to G | CAG/CGG | Glutamine/Arginine | 9. aa |
| 112. nt | G change to A | GAT/AAT | Aspartic acid/Asparagine | 38. aa |
| 119. nt | C change to T | CCC/CTC | Proline/Leucine | 40. aa |
| 701. nt | C change to T | ACG/ATG | Threonine/Methionine | 234. aa |
| 719. nt | C change to G | ACC/AGC | Threonine/Serine | 240. aa |
| 968. nt | C change to T | ACC/ATC | Threonine/Isoleucine | 323. aa |
| 1016. nt | G change to A | AGT/AAT | Serine/Asparagine | 339. aa |
| 1219. nt | G change to A | GAG/AAG | Glutamic acid/Lysine | 407. aa |
| 1413. nt | A change to C | CAA/CAC | Glutamine/Histidine | 471. aa |
| 1414. nt | G change to A | GAT/AAT | Aspartic acid/Asparagine | 472. aa |
|  |  |  |  |  |

**Note:** Num. RP: Number of replacement polymorphisms, N.No:Nucleotide numberR.No:Residue number.

**Supplementary Table 11-C2:** Biochemical characteristics of amino acid changes obseved in *L. brevis gad1* gene.

| **R.No** | **Amino Acid**  **1-Letter Code** | | **Class** | **Charge Features** | | | **Polarity** | | **Interaction Modes** | **B** | **pI** | **H** | **Sing.** | **PROVEAN** |
| --- | --- | --- | --- | --- | --- | --- | --- | --- | --- | --- | --- | --- | --- | --- |
|  |  |  |  | **Positive** | **Negative** | **Neutral** | **Polar** | **Nonpolar** |  |  |  |  |  |  |
| **9** | Glutamine | Q | Amide | - | - | YES | YES | - | H-bonds, van der Waals | 5 | 5.7 | 0.430 | YES | Neutral |
|  | Arginine | R | Basic | YES | - | - | YES | - | Ionic, H-bonds, van der Waals | 7 | 10.8 | 0.000 |  |  |
| **38** | Aspartic acid | D | Acid | - | YES | - | YES | - | Ionic, H-bonds, van der Waals | 4 | 3.0 | 0.417 | YES | Neutral |
|  | Asparagine | N | Amide | - | - | YES | YES | - | H-bonds, van der Waals | 5 | 5.4 | 0.448 |  |  |
| **40** | Proline | P | Cyclic | - | - | YES | - | YES | van der Waals | 0 | 6.3 | 0.678 | YES | Deleterious |
|  | Leucine | L | Aliphatic | - | - | YES | - | YES | van der Waals | 0 | 6.0 | 0.918 |  |  |
| **234** | Threonine | T | Hydroxylic | - | - | YES | YES | - | H-bonds, van der Waals | 3 | 5.6 | 0.634 | YES | Neutral |
|  | Methionine | M | Sulfuric | - | - | YES | - | YES | van der Waals | 0 | 5.7 | 0.811 |  |  |
| **240** | Threonine | T | Hydroxylic | - | - | YES | YES | - | H-bonds, van der Waals | 3 | 5.6 | 0.634 | NO | Neutral |
|  | Serine | S | Hydroxylic | - | - | YES | YES | - | H-bonds, van der Waals | 3 | 5.7 | 0.601 |  |  |
| **323** | Threonine | T | Hydroxylic | - | - | YES | YES | - | H-bonds, van der Waals | 3 | 5.6 | 0.634 | YES | Neutral |
|  | Isoleucine | I | Aliphatic | - | - | YES | - | YES | van der Waals | 0 | 6.0 | 1,000 |  |  |
| **339** | Serine | S | Hydroxylic | - | - | YES | YES | - | H-bonds, van der Waals | 3 | 5.7 | 0.601 | NO | Neutral |
|  | Asparagine | N | Amide | - | - | YES | YES | - | H-bonds, van der Waals | 5 | 5.4 | 0.448 |  |  |
| **407** | Glutamic acid | E | Acid | - | YES | - | YES | - | Ionic, H-bonds, van der Waals | 4 | 3.2 | 0.458 | YES | Neutral |
|  | Lysine | K | Basic | YES | - | - | YES | - | Ionic, H-bonds, van der Waals | 3 | 9.7 | 0.263 |  |  |
| **471** | Glutamine | Q | Amide | - | - | YES | YES | - | H-bonds, van der Waals | 5 | 5.7 | 0.430 | NO | Neutral |
|  | Histidine | H | Basic aromatic | YES | - | - | YES | - | Ionic, H-bonds, aromatic stacking, van der Waals | 3 | 7.6 | 0.548 |  |  |
| **472** | Aspartic acid | D | Acid | - | YES | - | YES | - | Ionic, H-bonds, van der Waals | 4 | 3.0 | 0.417 | YES | Neutral |
|  | Asparagine | N | Amide | - | - | YES | YES | - | H-bonds, van der Waals | 5 | 5.4 | 0.448 |  |  |

**Note:** R.No:Residue number, B:Potential side chain H-bonds, pI:Isoelectric point, H:Hydrophobicity, Sing.: Singleton

**Supplementary Table 11-D1:** Replacement(Nonsynonymous) sites in the *L. brevis gad2* gene and their corresponding amino acids on the protein. The nucleotides changed in the triple codon structure, and the new amino acids formed by the change are shown in red.

| *L. brevis*  *gad2*  1407 nt | **Isolation Group** | **Num. RP** | **Nucleotide Position of RP** | |
| --- | --- | --- | --- | --- |
|  | Fermented Vegetable | 8 | 50-68-458-468-679-685-984-1246 | |
|  | Sourdough | 8 | 50-68-415-458-468-518-671-1246 | |
|  | Fermented Dairy Products | 9 | 34-46-50-68-120-458-671-973-1246 | |
|  | Fermented Beverage | 9 | 50-68-281-458-468-679-685-1058-1246 | |
|  | Feces | 7 | 50-68-458-468-469-671-1246 | |
|  |  |  |  | |
| **Amino acid Changes on *L. brevis gad2*** | | | | |
| **N.No** | **Nucleotide Changes** | **Codons** | **Amino acid Change** | **R.No** |
| 34. nt | A change to G | ACA/GCA | Threonine/Alanine | 12. aa |
| 46. nt | C change to T | CTC/TTC | Leucine/Phenylalanine | 16. aa |
| 50. nt | C change to A | ACA/AAA | Threonine/Lysine | 17. aa |
| 68. nt | G change to C | AGC/ACC | Serine/Threonine | 23. aa |
| 120. nt | G change to T | GAG/GAT | Glutamic acid/Aspartic acid | 40. aa |
| 281. nt | G change to A | CGG/CAG | Arginine/Glutamine | 94. aa |
| 415. nt | T change to C | TTT/CTT | Phenylalanine/Leucine | 139. aa |
| 458. nt | C change to A | ACT/AAT | Threonine/Asparagine | 153. aa |
| 468. nt | A change to C | CAA/CAC | Glutamine/Histidine | 156. aa |
| 469. nt | C change to T | CCT/TCT | Proline/Serine | 157. aa |
| 518. nt | G change to C | TGT/TCT | Cysteine/Serine | 173. aa |
| 671. nt | C change to T | GCC/GTC | Alanine/Valine | 224. aa |
| 679. nt | G change to A | GAT/AAT | Aspartic acid/Asparagine | 227. aa |
| 685. nt | G change to A | GTT/ATT | Valine/Isoleucine | 229. aa |
| 973. nt | T change to G | TCC/GCC | Serine/Alanine | 325. aa |
| 984. nt | C change to G | ATC/ATG | Isoleucine/Methionine | 328. aa |
| 1058. nt | C change to T | GCC/GTC | Alanine/Valine | 353. aa |
| 1246. nt | G change to A | GCG/ACG | Alanine/Threonine | 416. aa |
|  |  |  |  |  |

**Note:** Num. RP: Number of replacement polymorphisms, N.No:Nucleotide number, R.No:Residue number.

**Supplementary Table 11-D2:** Biochemical characteristics of amino acid changes obseved in *L. brevis gad2* gene.

| **R.No** | **Amino Acid**  **1-Letter Code** | | **Class** | **Charge Features** | | | **Polarity** | | **Interaction Modes** | **B** | **pI** | **H** | **Sing.** | **PROVEAN** |
| --- | --- | --- | --- | --- | --- | --- | --- | --- | --- | --- | --- | --- | --- | --- |
|  |  |  |  | **Positive** | **Negative** | **Neutral** | **Polar** | **Nonpolar** |  |  |  |  |  |  |
| **12** | Threonine | T | Hydroxylic | - | - | YES | YES | - | H-bonds, van der Waals | 3 | 5.6 | 0.634 | YES | Neutral |
|  | Alanine | A | Aliphatic | - | - | YES | - | YES | van der Waals | 0 | 6.0 | 0.806 |  |  |
| **16** | Leucine | L | Aliphatic | - | - | YES | - | YES | van der Waals | 0 | 6.0 | 0.918 | YES | Neutral |
|  | Phenylalanine | F | Aromatic | - | - | YES | - | YES | Aromatic stacking, van der Waals | 0 | 5.5 | 0.951 |  |  |
| **17** | Threonine | T | Hydroxylic | - | - | YES | YES | - | H-bonds, van der Waals | 3 | 5.6 | 0.634 | NO | Neutral |
|  | Lysine | K | Basic | YES | - | - | YES | - | Ionic, H-bonds, van der Waals | 3 | 9.7 | 0.263 |  |  |
| **23** | Threonine | T | Hydroxylic | - | - | YES | YES | - | H-bonds, van der Waals | 3 | 5.6 | 0.634 | NO | Neutral |
|  | Serine | S | Hydroxylic | - | - | YES | YES | - | H-bonds, van der Waals | 3 | 5.7 | 0.601 |  |  |
| **40** | Glutamic acid | E | Acid | - | YES | - | YES | - | Ionic, H-bonds, van der Waals | 4 | 3.2 | 0.458 | YES | Neutral |
|  | Aspartic acid | D | Acid | - | YES | - | YES | - | Ionic, H-bonds, van der Waals | 4 | 3.0 | 0.417 |  |  |
| **94** | Arginine | R | Basic | YES | - | - | YES | - | Ionic, H-bonds, van der Waals | 7 | 10.8 | 0.000 | YES | Neutral |
|  | Glutamine | Q | Amide | - | - | YES | YES | - | H-bonds, van der Waals | 5 | 5.7 | 0.430 |  |  |
| **139** | Phenylalanine | F | Aromatic | - | - | YES | - | YES | Aromatic stacking, van der Waals | 0 | 5.5 | 0.951 | YES | Deleterious |
|  | Leucine | L | Aliphatic | - | - | YES | - | YES | van der Waals | 0 | 6.0 | 0.918 |  |  |
| **153** | Threonine | T | Hydroxylic | - | - | YES | YES | - | H-bonds, van der Waals | 3 | 5.6 | 0.634 | NO | Neutral |
|  | Asparagine | N | Amide | - | - | YES | YES | - | H-bonds, van der Waals | 5 | 5.4 | 0.448 |  |  |
| **156** | Glutamine | Q | Amide | - | - | YES | YES | - | H-bonds, van der Waals | 5 | 5.7 | 0.430 | NO | Neutral |
|  | Histidine | H | Basic aromatic | YES | - | - | YES | - | Ionic, H-bonds, aromatic stacking, van der Waals | 3 | 7.6 | 0.548 |  |  |

(cont. on next page)

**Supplementary Table 11-D2:** (cont.)

| **R.No** | **Amino Acid**  **1-Letter Code** | | **Class** | **Charge Features** | | | **Polarity** | | **Interaction Modes** | **B** | **pI** | **H** | **Sing.** | **PROVEAN** |
| --- | --- | --- | --- | --- | --- | --- | --- | --- | --- | --- | --- | --- | --- | --- |
|  |  |  |  | **Positive** | **Negative** | **Neutral** | **Polar** | **Nonpolar** |  |  |  |  |  |  |
| **157** | Proline | P | Cyclic | - | - | YES | - | YES | van der Waals | 0 | 6.3 | 0.678 | YES | Deleterious |
|  | Serine | S | Hydroxylic | - | - | YES | YES | - | H-bonds, van der Waals | 3 | 5.7 | 0.601 |  |  |
| **173** | Cysteine | C | Sulfuric | - | - | YES | YES | - | Covalent disulfide bonds, van der Waals | 0 | 5.0 | 0.721 | YES | Deleterious |
|  | Serine | S | Hydroxylic | - | - | YES | YES | - | H-bonds, van der Waals | 3 | 5.7 | 0.601 |  |  |
| **224** | Alanine | A | Aliphatic | - | - | YES | - | YES | van der Waals | 0 | 6.0 | 0.806 | NO | Neutral |
|  | Valine | V | Aliphatic | - | - | YES | - | YES | van der Waals | 0 | 6.0 | 0.923 |  |  |
| **227** | Aspartic acid | D | Acid | - | YES | - | YES | - | Ionic, H-bonds, van der Waals | 4 | 3.0 | 0.417 | NO | Neutral |
|  | Asparagine | N | Amide | - | - | YES | YES | - | H-bonds, van der Waals | 5 | 5.4 | 0.448 |  |  |
| **229** | Valine | V | Aliphatic | - | - | YES | - | YES | van der Waals | 0 | 6.0 | 0.923 | NO | Neutral |
|  | Isoleucine | I | Aliphatic | - | - | YES | - | YES | van der Waals | 0 | 6.0 | 1,000 |  |  |
| **325** | Serine | S | Hydroxylic | - | - | YES | YES | - | H-bonds, van der Waals | 3 | 5.7 | 0.601 | YES | Neutral |
|  | Alanine | A | Aliphatic | - | - | YES | - | YES | van der Waals | 0 | 6.0 | 0.806 |  |  |
| **328** | Isoleucine | I | Aliphatic | - | - | YES | - | YES | van der Waals | 0 | 6.0 | 1,000 | YES | Neutral |
|  | Methionine | M | Sulfuric | - | - | YES | - | YES | van der Waals | 0 | 5.7 | 0.811 |  |  |
| **353** | Alanine | A | Aliphatic | - | - | YES | - | YES | van der Waals | 0 | 6.0 | 0.806 | YES | Deleterious |
|  | Valine | V | Aliphatic | - | - | YES | - | YES | van der Waals | 0 | 6.0 | 0.923 |  |  |
| **416** | Alanine | A | Aliphatic | - | - | YES | - | YES | van der Waals | 0 | 6.0 | 0.806 | NO | Neutral |
|  | Threonine | T | Hydroxylic | - | - | YES | YES | - | H-bonds, van der Waals | 3 | 5.6 | 0.634 |  |  |

**Note:** R.No:Residue number, B:Potential side chain H-bonds, pI:Isoelectric point, H:Hydrophobicity, Sing.: Singleton

**Supplementary Table 12-A1:** Replacement(Nonsynonymous) sites in the *L. plantarum gadB* gene and their corresponding amino acids on the protein. The nucleotides changed in the triple codon structure, and the new amino acids formed by the change are shown in red.

| *L. plantarum*  *gadB*  1410 nt | **Isolation Group** | **Num. RP** | **Nucleotide Position of RP** | |
| --- | --- | --- | --- | --- |
|  | Kimchi | 20 | 340-341-418-419-500-540-544-572-580-583-595-658-674-704-712-1022-1108-1153-1337-1353 | |
|  | Unspecified  Femented Food | 14 | 52-500-540-544-583-595-704-712-901-1019-1022-1023-1153-1353 | |
|  | Raw Food | 4 | 514-658-1153-1353 | |
|  | Kefir | 11 | 40-500-544-572-583-595-658-704-712-953-1353 | |
|  | Cheese | 23 | 53-98-340-341-418-419-425-500-540-544-580-583-595-674-704-712-764-793-953-1022-1108-1153-1353 | |
|  | Beverage | 6 | 540-683-850-871-951-1353 | |
|  | Meat | 11 | 500-544-583-595-704-712-850-951-1022-1108-1353 | |
|  | Feces | 4 | 1105-1108-1153-1353 | |
|  | Other Milk-based Products | 14 | 500-540-544-572-583-595-658-704-712-793-934-953-1022-1353 | |
| **Amino acid Changes on *L. plantarum gadB*** | | | | |
| **N.No** | **Nucleotide Changes** | **Codons** | **Amino acid Change** | **R.No** |
| 40. nt | G change to A | GAA/AAA | Glutamic acid/Lysine | 14. aa |
| 52. nt | C change to A | CCA/ACA | Proline/Threonine | 18. aa |
| 53. nt | C change to T | CCA/CTA | Proline/Leucine | 18. aa |
| 98. nt | G change to T | CGG/CTG | Arginine/Leucine | 33. aa |
| 340. nt | G change to A | GAT/AGT | Aspartic acid/Serine | 114. aa |
| 341. nt | A change to G |  |  |  |
| 418. nt | G change to A | GCC/AGT | Alanine/Serine | 140. aa |
| 419. nt | C change to G |  |  |  |
| 425. nt | G change to T | CGT/CTT | Arginine/Leucine | 142. aa |
| 500. nt | T change to C | GTT/GCT | Valine/Alanine | 167. aa |
| 514. nt | T change to G | TTT/GTT | Phenylalanine/Valine | 172. aa |
| 540. nt | G change to A | ATG/ATA | Methionine/Isoleucine | 180. aa |
| 544. nt | G change to A | GTG/ATG | Valine/Methionine | 182. aa |
| 572. nt | C change to T | GCC/GTC | Alanine/Valine | 191. aa |
| 580. nt | G change to A | GTT/ATT | Valine/Isoleucine | 194. aa |
| 583. nt | A change to G | AAC/GAC | Asparagine/Aspartic acid | 195. aa |
| 595. nt | G change to A | GAC/AAC | Aspartic acid/Asparagine | 199. aa |
| 658. nt | T change to G | TAT/GAT | Tyrosine/Aspartic acid | 220. aa |
| 674. nt | C change to G | GCA/GGA | Alanine/Glycine | 225. aa |
| 683. nt | A change to G | AAG/AGG | Lysine/Arginine | 228. aa |
| 704. nt | A change to G | CAT/CGT | Histidine/Arginine | 235. aa |
| 712. nt | C change to T | CCC/TCC | Proline/Serine | 238. aa |
| 764. nt | C change to T | ACC/ATC | Threonine/Isoleucine | 255. aa |
| 793. nt | G change to A | GAC/AAC | Aspartic acid/Asparagine | 265. aa |
| 850. nt | G change to A | GTT/ATT | Valine/Isoleucine | 284. aa |
| 871. nt | G change to A | GTC/ATC | Valine/Isoleucine | 291. aa |
| 901. nt | C change to T | CCA/TCA | Proline/Serine | 301. aa |

(cont. on next page)

**Supplementary Table 12-A1:** (cont.)

| **N.No** | **Nucleotide Changes** | **Codons** | **Amino acid Change** | **R.No** |
| --- | --- | --- | --- | --- |
| 934. nt | G change to A | GGG/AGG | Glycine/Arginine | 312. aa |
| 951. nt | G change to A | ATG/ATA | Methionine/Isoleucine | 317. aa |
| 953. nt | C change to T | GCG/GTG | Alanine/Valine | 318. aa |
| 1019. nt | T change to A | ATG/AAG | Methionine/Lysine | 340. aa |
| 1022. nt | A change to G | GAC/GGC | Aspartic acid/Glycine | 341. aa |
| 1023. nt | C change to A | GAC/GAA | Aspartic acid/Glutamic acid | 341. aa |
| 1105. nt | A change to G | ATG/GTG | Methionine/Valine | 369. aa |
| 1108. nt | A change to G | ATC/GTC | Isoleucine/Valine | 370. aa |
| 1153. nt | C change to T | CCG/TCG | Proline/Serine | 385. aa |
| 1337. nt | T change to G | GTC/GGC | Valine/Glycine | 446. aa |
| 1353. nt | A change to C | CAA/CAC | Glutamine/Histidine | 451. aa |
|  |  |  |  |  |

**Note:** Num. RP: Number of replacement polymorphisms, N.No:Nucleotide number, R.No:Residue number.

**Supplementary Table 12-A2:** Biochemical characteristics of amino acid changes obseved in *L. plantarum gadB* gene.

| **R.No** | **Amino Acid**  **1-Letter Code** | | **Class** | **Charge Features** | | | **Polarity** | | **Interaction Modes** | **B** | **pI** | **H** | **Sing.** | **PROVEAN** |
| --- | --- | --- | --- | --- | --- | --- | --- | --- | --- | --- | --- | --- | --- | --- |
|  |  |  |  | **Positive** | **Negative** | **Neutral** | **Polar** | **Nonpolar** |  |  |  |  |  |  |
| **14** | Glutamic acid | E | Acid | - | YES | - | YES | - | Ionic, H-bonds, van der Waals | 4 | 3.2 | 0.458 | YES | Neutral |
|  | Lysine | K | Basic | YES | - | - | YES | - | Ionic, H-bonds, van der Waals | 3 | 9.7 | 0.263 |  |  |
| **18** | Proline | P | Cyclic | - | - | YES | - | YES | van der Waals | 0 | 6.3 | 0.678 | YES | Deleterious |
|  | Threonine | T | Hydroxylic | - | - | YES | YES | - | H-bonds, van der Waals | 3 | 5.6 | 0.634 |  |  |
| **18** | Proline | P | Cyclic | - | - | YES | - | YES | van der Waals | 0 | 6.3 | 0.678 | NO | Deleterious |
|  | Leucine | L | Aliphatic | - | - | YES | - | YES | van der Waals | 0 | 6.0 | 0.918 |  |  |
| **33** | Arginine | R | Basic | YES | - | - | YES | - | Ionic, H-bonds, van der Waals | 7 | 10.8 | 0.000 | YES | Deleterious |
|  | Leucine | L | Aliphatic | - | - | YES | - | YES | van der Waals | 0 | 6.0 | 0.918 |  |  |
| **114** | Aspartic acid | D | Acid | - | YES | - | YES | - | Ionic, H-bonds, van der Waals | 4 | 3.0 | 0.417 | NO | Neutral |
|  | Serine | S | Hydroxylic | - | - | YES | YES | - | H-bonds, van der Waals | 3 | 5.7 | 0.601 |  |  |
| **140** | Alanine | A | Aliphatic | - | - | YES | - | YES | van der Waals | 0 | 6.0 | 0.806 | NO | Neutral |
|  | Serine | S | Hydroxylic | - | - | YES | YES | - | H-bonds, van der Waals | 3 | 5.7 | 0.601 |  |  |
| **142** | Arginine | R | Basic | YES | - | - | YES | - | Ionic, H-bonds, van der Waals | 7 | 10.8 | 0.000 | NO | Deleterious |
|  | Leucine | L | Aliphatic | - | - | YES | - | YES | van der Waals | 0 | 6.0 | 0.918 |  |  |
| **167** | Valine | V | Aliphatic | - | - | YES | - | YES | van der Waals | 0 | 6.0 | 0.923 | NO | Deleterious |
|  | Alanine | A | Aliphatic | - | - | YES | - | YES | van der Waals | 0 | 6.0 | 0.806 |  |  |
| **172** | Phenylalanine | F | Aromatic | - | - | YES | - | YES | Aromatic stacking, van der Waals | 0 | 5.5 | 0.951 | YES | Deleterious |
|  | Valine | V | Aliphatic | - | - | YES | - | YES | van der Waals | 0 | 6.0 | 0.923 |  |  |
| **180** | Methionine | M | Sulfuric | - | - | YES | - | YES | van der Waals | 0 | 5.7 | 0.811 | NO | Neutral |
|  | Isoleucine | I | Aliphatic | - | - | YES | - | YES | van der Waals | 0 | 6.0 | 1,000 |  |  |

(cont. on next page)

**Supplementary Table 12-A2:** (cont.)

| **R.No** | **Amino Acid**  **1-Letter Code** | | **Class** | **Charge Features** | | | **Polarity** | | **Interaction Modes** | **B** | **pI** | **H** | **Sing.** | **PROVEAN** |
| --- | --- | --- | --- | --- | --- | --- | --- | --- | --- | --- | --- | --- | --- | --- |
|  |  |  |  | **Positive** | **Negative** | **Neutral** | **Polar** | **Nonpolar** |  |  |  |  |  |  |
| **182** | Valine | V | Aliphatic | - | - | YES | - | YES | van der Waals | 0 | 6.0 | 0.923 | NO | Neutral |
|  | Methionine | M | Sulfuric | - | - | YES | - | YES | van der Waals | 0 | 5.7 | 0.811 |  |  |
| **191** | Alanine | A | Aliphatic | - | - | YES | - | YES | van der Waals | 0 | 6.0 | 0.806 | NO | Neutral |
|  | Valine | V | Aliphatic | - | - | YES | - | YES | van der Waals | 0 | 6.0 | 0.923 |  |  |
| **194** | Valine | V | Aliphatic | - | - | YES | - | YES | van der Waals | 0 | 6.0 | 0.923 | NO | Neutral |
|  | Isoleucine | I | Aliphatic | - | - | YES | - | YES | van der Waals | 0 | 6.0 | 1,000 |  |  |
| **195** | Asparagine | N | Amide | - | - | YES | YES | - | H-bonds, van der Waals | 5 | 5.4 | 0.448 | NO | Neutral |
|  | Aspartic acid | D | Acid | - | YES | - | YES | - | Ionic, H-bonds, van der Waals | 4 | 3.0 | 0.417 |  |  |
| **199** | Aspartic acid | D | Acid | - | YES | - | YES | - | Ionic, H-bonds, van der Waals | 4 | 3.0 | 0.417 | NO | Neutral |
|  | Asparagine | N | Amide | - | - | YES | YES | - | H-bonds, van der Waals | 5 | 5.4 | 0.448 |  |  |
| **220** | Tyrosine | Y | Aromatic | - | - | YES | YES | - | Ionic, H-bonds, aromatic stacking, van der Waals | 8? | 5.6 | ? | NO | Deleterious |
|  | Aspartic acid | D | Acid | - | YES | - | YES | - | Ionic, H-bonds, van der Waals | 4 | 3.0 | 0.417 |  |  |
| **225** | Alanine | A | Aliphatic | - | - | YES | - | YES | van der Waals | 0 | 6.0 | 0.806 | NO | Deleterious |
|  | Glycine | G | Aliphatic | - | - | YES | - | YES | van der Waals | 0 | 6.0 | 0.770 |  |  |
| **228** | Lysine | K | Basic | YES | - | - | YES | - | Ionic, H-bonds, van der Waals | 3 | 9.7 | 0.263 | NO | Neutral |
|  | Arginine | R | Basic | YES | - | - | YES | - | Ionic, H-bonds, van der Waals | 7 | 10.8 | 0.000 |  |  |
| **235** | Histidine | H | Basic aromatic | YES | - | - | YES | - | Ionic, H-bonds, aromatic stacking, van der Waals | 3 | 7.6 | 0.548 | NO | Neutral |
|  | Arginine | R | Basic | YES | - | - | YES | - | Ionic, H-bonds, van der Waals | 7 | 10.8 | 0.000 |  |  |
| **238** | Proline | P | Cyclic | - | - | YES | - | YES | van der Waals | 0 | 6.3 | 0.678 | NO | Neutral |
|  | Serine | S | Hydroxylic | - | - | YES | YES | - | H-bonds, van der Waals | 3 | 5.7 | 0.601 |  |  |

(cont. on next page)

**Supplementary Table 12-A2:** (cont.)

| **R.No** | **Amino Acid**  **1-Letter Code** | | **Class** | **Charge Features** | | | **Polarity** | | **Interaction Modes** | **B** | **pI** | **H** | **Sing.** | **PROVEAN** |
| --- | --- | --- | --- | --- | --- | --- | --- | --- | --- | --- | --- | --- | --- | --- |
|  |  |  |  | **Positive** | **Negative** | **Neutral** | **Polar** | **Nonpolar** |  |  |  |  |  |  |
| **255** | Threonine | T | Hydroxylic | - | - | YES | YES | - | H-bonds, van der Waals | 3 | 5.6 | 0.634 | YES | Neutral |
|  | Isoleucine | I | Aliphatic | - | - | YES | - | YES | van der Waals | 0 | 6.0 | 1,000 |  |  |
| **265** | Aspartic acid | D | Acid | - | YES | - | YES | - | Ionic, H-bonds, van der Waals | 4 | 3.0 | 0.417 | NO | Deleterious |
|  | Asparagine | N | Amide | - | - | YES | YES | - | H-bonds, van der Waals | 5 | 5.4 | 0.448 |  |  |
| **284** | Valine | V | Aliphatic | - | - | YES | - | YES | van der Waals | 0 | 6.0 | 0.923 | NO | Neutral |
|  | Isoleucine | I | Aliphatic | - | - | YES | - | YES | van der Waals | 0 | 6.0 | 1,000 |  |  |
| **291** | Valine | V | Aliphatic | - | - | YES | - | YES | van der Waals | 0 | 6.0 | 0.923 | YES | Neutral |
|  | Isoleucine | I | Aliphatic | - | - | YES | - | YES | van der Waals | 0 | 6.0 | 1,000 |  |  |
| **301** | Proline | P | Cyclic | - | - | YES | - | YES | van der Waals | 0 | 6.3 | 0.678 | YES | Neutral |
|  | Serine | S | Hydroxylic | - | - | YES | YES | - | H-bonds, van der Waals | 3 | 5.7 | 0.601 |  |  |
| **312** | Glycine | G | Aliphatic | - | - | YES | - | YES | van der Waals | 0 | 6.0 | 0.770 | YES | Deleterious |
|  | Arginine | R | Basic | YES | - | - | YES | - | Ionic, H-bonds, van der Waals | 7 | 10.8 | 0.000 |  |  |
| **317** | Methionine | M | Sulfuric | - | - | YES | - | YES | van der Waals | 0 | 5.7 | 0.811 | NO | Neutral |
|  | Isoleucine | I | Aliphatic | - | - | YES | - | YES | van der Waals | 0 | 6.0 | 1,000 |  |  |
| **318** | Alanine | A | Aliphatic | - | - | YES | - | YES | van der Waals | 0 | 6.0 | 0.806 | NO | Deleterious |
|  | Valine | V | Aliphatic | - | - | YES | - | YES | van der Waals | 0 | 6.0 | 0.923 |  |  |
| **340** | Methionine | M | Sulfuric | - | - | YES | - | YES | van der Waals | 0 | 5.7 | 0.811 | YES | Neutral |
|  | Lysine | K | Basic | YES | - | - | YES | - | Ionic, H-bonds, van der Waals | 3 | 9.7 | 0.263 |  |  |
| **341** | Aspartic acid | D | Acid | - | YES | - | YES | - | Ionic, H-bonds, van der Waals | 4 | 3.0 | 0.417 | NO | Deleterious |
|  | Glycine | G | Aliphatic | - | - | YES | - | YES | van der Waals | 0 | 6.0 | 0.770 |  |  |

(cont. on next page)

**Supplementary Table 12-A2:** (cont.)

| **R.No** | **Amino Acid**  **1-Letter Code** | | **Class** | **Charge Features** | | | **Polarity** | | **Interaction Modes** | **B** | **pI** | **H** | **Sing.** | **PROVEAN** |
| --- | --- | --- | --- | --- | --- | --- | --- | --- | --- | --- | --- | --- | --- | --- |
|  |  |  |  | **Positive** | **Negative** | **Neutral** | **Polar** | **Nonpolar** |  |  |  |  |  |  |
| **369** | Methionine | M | Sulfuric | - | - | YES | - | YES | van der Waals | 0 | 5.7 | 0.811 | YES | Neutral |
|  | Valine | V | Aliphatic | - | - | YES | - | YES | van der Waals | 0 | 6.0 | 0.923 |  |  |
| **370** | Isoleucine | I | Aliphatic | - | - | YES | - | YES | van der Waals | 0 | 6.0 | 1,000 | NO | Neutral |
|  | Valine | V | Aliphatic | - | - | YES | - | YES | van der Waals | 0 | 6.0 | 0.923 |  |  |
| **385** | Proline | P | Cyclic | - | - | YES | - | YES | van der Waals | 0 | 6.3 | 0.678 | NO | Neutral |
|  | Serine | S | Hydroxylic | - | - | YES | YES | - | H-bonds, van der Waals | 3 | 5.7 | 0.601 |  |  |
| **446** | Valine | V | Aliphatic | - | - | YES | - | YES | van der Waals | 0 | 6.0 | 0.923 | NO | Deleterious |
|  | Glycine | G | Aliphatic | - | - | YES | - | YES | van der Waals | 0 | 6.0 | 0.770 |  |  |
| **451** | Glutamine | Q | Amide | - | - | YES | YES | - | H-bonds, van der Waals | 5 | 5.7 | 0.430 | NO | Neutral |
|  | Histidine | H | Basic aromatic | YES | - | - | YES | - | Ionic, H-bonds, aromatic stacking, van der Waals | 3 | 7.6 | 0.548 |  |  |
|  |  |  |  |  |  |  |  |  |  |  |  |  |  |  |

**Note:** R.No:Residue number, B:Potential side chain H-bonds, pI:Isoelectric point, H:Hydrophobicity, Sing.: Singleton

**Supplementary Table 12-B1:** Replacement(Nonsynonymous) sites in the *L. plantarum yjeM* gene and their corresponding amino acids on the protein. The nucleotides changed in the triple codon structure, and the new amino acids formed by the change are shown in red.

| *L. plantarum*  *yjeM*  1485 nt | **Isolation Group** | **Num. RP** | **Nucleotide Position of RP** | |
| --- | --- | --- | --- | --- |
|  | Kimchi | 7 | 379-400-409-898-1300-1387 | |
|  | Unspecified  Femented Food | 3 | 409-898-1300 | |
|  | Raw Food | 1 | 1300 | |
|  | Kefir | 4 | 400-592-898-1076 | |
|  | Cheese | 7 | 898-923-1021-1238-1300-1385-1438 | |
|  | Beverage | 2 | 1076-1300 | |
|  | Meat | 3 | 400-898-1300 | |
|  | Feces | 3 | 409-898-1387 | |
|  | Other Milk-based Products | 4 | 409-592-898-1300 | |
| **Amino acid Changes on *L. plantarum gadB*** | | | | |
| **N.No** | **Nucleotide Changes** | **Codons** | **Amino acid Change** | **R.No** |
| 379. nt | T change to C | TTT/CTT | Phenylalanine/Leucine | 127. aa |
| 400. nt | A change to G | ATG/GTG | Methionine/Valine | 134. aa |
| 409. nt | A change to C | ATT/CTT | Isoleucine/Leucine | 137. aa |
| 592. nt | A change to G | ATG/GTG | Methionine/Valine | 198. aa |
| 896. nt | C change to T | GCG/GTG | Alanine/Valine | 299. aa |
| 898. nt | C change to T | CTT/TTT | Leucine/Phenylalanine | 300. aa |
| 923. nt | A change to C | AAG/ACG | Lysine/Threonine | 308. aa |
| 1021. nt | C change to A | CTT/ATT | Leucine/Isoleucine | 341. aa |
| 1076. nt | A change to G | AAA/AGA | Lysine/Arginine | 359. aa |
| 1238. nt | C change to T | GCG/GTG | Alanine/Valine | 413. aa |
| 1300. nt | A change to G | ACT/GCT | Threonine/Alanine | 434. aa |
| 1385. nt | C change to A | ACT/AAT | Threonine/Asparagine | 462. aa |
| 1387. nt | G change to A | GGG/AGG | Glycine/Arginine | 463. aa |
| 1438. nt | G change to T | GTG/TTG | Valine/Leucine | 480. aa |
|  |  |  |  |  |

**Note:** Num. RP: Number of replacement polymorphisms, N.No:Nucleotide number, R.No:Residue number.

**Supplementary Table 12-B2:** Biochemical characteristics of amino acid changes obseved in *L. plantarum yjeM* gene.

| **R.No** | **Amino Acid**  **1-Letter Code** | | **Class** | **Charge Features** | | | **Polarity** | | **Interaction Modes** | **B** | **pI** | **H** | **Sing.** | **PROVEAN** |
| --- | --- | --- | --- | --- | --- | --- | --- | --- | --- | --- | --- | --- | --- | --- |
|  |  |  |  | **Positive** | **Negative** | **Neutral** | **Polar** | **Nonpolar** |  |  |  |  |  |  |
| **127** | Phenylalanine | F | Aromatic | - | - | YES | - | YES | Aromatic stacking, van der Waals | 0 | 5.5 | 0.951 | YES | Neutral |
|  | Leucine | L | Aliphatic | - | - | YES | - | YES | van der Waals | 0 | 6.0 | 0.918 |  |  |
| **134** | Methionine | M | Sulfuric | - | - | YES | - | YES | van der Waals | 0 | 5.7 | 0.811 | NO | Neutral |
|  | Valine | V | Aliphatic | - | - | YES | - | YES | van der Waals | 0 | 6.0 | 0.923 |  |  |
| **137** | Isoleucine | I | Aliphatic | - | - | YES | - | YES | van der Waals | 0 | 6.0 | 1,000 | NO | Neutral |
|  | Leucine | L | Aliphatic | - | - | YES | - | YES | van der Waals | 0 | 6.0 | 0.918 |  |  |
| **198** | Methionine | M | Sulfuric | - | - | YES | - | YES | van der Waals | 0 | 5.7 | 0.811 | NO | Neutral |
|  | Valine | V | Aliphatic | - | - | YES | - | YES | van der Waals | 0 | 6.0 | 0.923 |  |  |
| **299** | Alanine | A | Aliphatic | - | - | YES | - | YES | van der Waals | 0 | 6.0 | 0.806 | YES | Deleterious |
|  | Valine | V | Aliphatic | - | - | YES | - | YES | van der Waals | 0 | 6.0 | 0.923 |  |  |
| **300** | Leucine | L | Aliphatic | - | - | YES | - | YES | van der Waals | 0 | 6.0 | 0.918 | NO | Neutral |
|  | Phenylalanine | F | Aromatic | - | - | YES | - | YES | Aromatic stacking, van der Waals | 0 | 5.5 | 0.951 |  |  |
| **308** | Lysine | K | Basic | YES | - | - | YES | - | Ionic, H-bonds, van der Waals | 3 | 9.7 | 0.263 | NO | Neutral |
|  | Threonine | T | Hydroxylic | - | - | YES | YES | - | H-bonds, van der Waals | 3 | 5.6 | 0.634 |  |  |
| **341** | Leucine | L | Aliphatic | - | - | YES | - | YES | van der Waals | 0 | 6.0 | 0.918 | YES | Neutral |
|  | Isoleucine | I | Aliphatic | - | - | YES | - | YES | van der Waals | 0 | 6.0 | 1,000 |  |  |
| **359** | Lysine | K | Basic | YES | - | - | YES | - | Ionic, H-bonds, van der Waals | 3 | 9.7 | 0.263 | NO | Neutral |
|  | Arginine | R | Basic | YES | - | - | YES | - | Ionic, H-bonds, van der Waals | 7 | 10.8 | 0.000 |  |  |

(cont. on next page)

**Supplementary Table 12-B2:** (cont.)

| **R.No** | **Amino Acid**  **1-Letter Code** | | **Class** | **Charge Features** | | | **Polarity** | | **Interaction Modes** | **B** | **pI** | **H** | **Sing.** | **PROVEAN** |
| --- | --- | --- | --- | --- | --- | --- | --- | --- | --- | --- | --- | --- | --- | --- |
|  |  |  |  | **Positive** | **Negative** | **Neutral** | **Polar** | **Nonpolar** |  |  |  |  |  |  |
| **413** | Alanine | A | Aliphatic | - | - | YES | - | YES | van der Waals | 0 | 6.0 | 0.806 | YES | Deleterious |
|  | Valine | V | Aliphatic | - | - | YES | - | YES | van der Waals | 0 | 6.0 | 0.923 |  |  |
| **434** | Threonine | T | Hydroxylic | - | - | YES | YES | - | H-bonds, van der Waals | 3 | 5.6 | 0.634 | NO | Neutral |
|  | Alanine | A | Aliphatic | - | - | YES | - | YES | van der Waals | 0 | 6.0 | 0.806 |  |  |
| **462** | Threonine | T | Hydroxylic | - | - | YES | YES | - | H-bonds, van der Waals | 3 | 5.6 | 0.634 | NO | Neutral |
|  | Asparagine | N | Amide | - | - | YES | YES | - | H-bonds, van der Waals | 5 | 5.4 | 0.448 |  |  |
| **463** | Glycine | G | Aliphatic | - | - | YES | - | YES | van der Waals | 0 | 6.0 | 0.770 | NO | Deleterious |
|  | Arginine | R | Basic | YES | - | - | YES | - | Ionic, H-bonds, van der Waals | 7 | 10.8 | 0.000 |  |  |
| **480** | Valine | V | Aliphatic | - | - | YES | - | YES | van der Waals | 0 | 6.0 | 0.923 | NO | Neutral |
|  | Leucine | L | Aliphatic | - | - | YES | - | YES | van der Waals | 0 | 6.0 | 0.918 |  |  |
|  |  |  |  |  |  |  |  |  |  |  |  |  |  |  |

**Note:** R.No:Residue number, B:Potential side chain H-bonds, pI:Isoelectric point, H:Hydrophobicity, Sing.: Singleton

**Supplementary Table 13-A:** The effect of amino acid changes on the overall pI and charge estimates of *L. brevis* GAD pathway proteins.

| ***L.brevis* GadR Protein 196 aa** | | | **Neutral** | | **Estimated Charge Over pH Range** | | | | | | | |
| --- | --- | --- | --- | --- | --- | --- | --- | --- | --- | --- | --- | --- |
| **Residue number on the protein** | **Number of Replacement Changes** | **Estimated pI** | **pH 7.00** | | **pH 4.00** | | **pH 4.50** | | **pH 5.00** | **pH 5.50** | **pH 6.00** | **pH 6.50** |
| No Replacements changes | 0 | 5.891 | -4.794 | | +19.856 | | +12.581 | | +6.554 | +2.546 | -0.667 | -3.232 |
| 55-70-81 | 3 | 5,894 | -4,802 | | +20.066 | | +12.791 | | +6.673 | +2.593 | -0.652 | -3.23 |
| 93 | 1 | 5.891 | -4.794 | | +19.856 | | +12.581 | | +6.554 | +2.546 | -0.667 | -3.232 |
| 191 | 1 | 5.891 | -4.794 | | +19.856 | | +12.581 | | +6.554 | +2.546 | -0.667 | -3.232 |
| ***L. brevis* GadC Protein 501 aa** | | | | | | | | | | | | |
| No Replacements changes | 0 | 8.996 | +6.179 | +30.118 | | +23.11 | | +17.334 | | +13.463 | +10.303 | +7.752 |
| 7-141-247-266-289-291-369 | 7 | 9.097 | +7.176 | +30.38 | | +23.639 | | +18.114 | | +14.381 | +11.276 | +8.743 |
| 27-438 | 2 | 8.996 | +6.179 | +30.118 | | +23.11 | | +17.334 | | +13.463 | +10.303 | +7.752 |
| 93 | 1 | 8.996 | +6.179 | +30.118 | | +23.11 | | +17.334 | | +13.463 | +10.303 | +7.752 |
| 171 | 1 | 8.996 | +6.179 | +30.118 | | +23.11 | | +17.334 | | +13.463 | +10.303 | +7.752 |
| 332 | 1 | 8.877 | +5.266 | +30.108 | | +23.078 | | +17.239 | | +13.214 | +9.792 | +6.984 |
| 343-367 | 2 | 8.876 | +5.18 | +29.647 | | +22.372 | | +16.435 | | +12.497 | +9.314 | +6.755 |
| 367 | 1 | 8.876 | +5.18 | +29.647 | | +22.372 | | +16.435 | | +12.497 | +9.314 | +6.755 |
| 369 | 1 | 8.996 | +6.179 | +30.118 | | +23.11 | | +17.334 | | +13.463 | +10.303 | +7.752 |
| ***L. brevis* Gad1 Protein 479 aa** | | | | | | | | | | | | |
| No Replacements changes | 0 | 4,905 | -21.359 | +26.51 | | +10.32 | | -1.877 | | -9.283 | -14.765 | -18.939 |
| 9 | 1 | 4.954 | -20.359 | +27.51 | | +11.32 | | -0.877 | | -8.283 | -13.765 | -17.939 |
| 38-234-407 | 3 | 5.045 | -18.364 | +28.243 | | +12.586 | | +0.802 | | -6.4 | -11.804 | -15.951 |
| 40 | 1 | 4,905 | -21.359 | +26.51 | | +10.32 | | -1.877 | | -9.283 | -14.765 | -18.939 |
| 240 | 1 | 4,905 | -21.359 | +26.51 | | +10.32 | | -1.877 | | -9.283 | -14.765 | -18.939 |
| 323 | 1 | 4,905 | -21.359 | +26.51 | | +10.32 | | -1.877 | | -9.283 | -14.765 | -18.939 |
| 339-471 | 2 | 4.950 | -21.272 | +27.499 | | +11.287 | | -0.972 | | -8.532 | -14.277 | -18.707 |
| 471 | 1 | 4.950 | -21.272 | +27.499 | | +11.287 | | -0.972 | | -8.532 | -14.277 | -18.707 |
| 472 | 1 | 4.949 | -20.36 | +26.981 | | +11.058 | | -0.978 | | -8.318 | -13.776 | -17.942 |

(cont. on next page)

**Supplementary Table 13-A:** (cont.)

| ***L. brevis* Gad2 Protein 468 aa** | | | | **Neutral** | | **Estimated Charge Over pH Range** | | | | | | | |
| --- | --- | --- | --- | --- | --- | --- | --- | --- | --- | --- | --- | --- | --- |
| **Residue no on the protein** | **Number of Replacement Changes** | **Estimated pI** | | **pH 7.00** | | **pH 4.00** | | **pH 4.50** | | **pH 5.00** | **pH 5.50** | **pH 6.00** | **pH 6.50** |
| No replacement changes | 0 | | 5.363 | -18.832 | +39.085 | | +21.164 | | +6.992 | | -2.238 | -9.655 | -15.493 |
| 12-16-23-40-325 | 5 | | 5.359 | -18.834 | +38.876 | | +20.955 | | +6.873 | | -2.285 | -9.671 | -15.499 |
| 17-416 | 2 | | 5.423 | -17.833 | +40.085 | | +22.164 | | +7.992 | | -1.238 | -8.655 | -14.494 |
| 17-328-416 | 3 | | 5.423 | -17.833 | +40.085 | | +22.164 | | +7.992 | | -1.238 | -8.655 | -14.494 |
| 17-224-416 | 3 | | 5.423 | -17.833 | +40.085 | | +22.164 | | +7.992 | | -1.238 | -8.655 | -14.494 |
| 17-153-224 | 3 | | 5.423 | -17.833 | +40.085 | | +22.164 | | +7.992 | | -1.238 | -8.655 | -14.494 |
| 23-153-156 | 3 | | 5.410 | -18.745 | +40.075 | | +22.132 | | +7.898 | | -1.487 | -9.167 | -15.261 |
| 23-94-153-156 | 4 | | 5.352 | -19.745 | +39.075 | | +21.132 | | +6.898 | | -2.487 | -10.167 | -16.261 |
| 23 | 1 | | 5.363 | -18.832 | +39.085 | | +21.164 | | +6.992 | | -2.238 | -9.655 | -15.493 |
| 23-153-156-157 | 4 | | 5.410 | -18.745 | +40.075 | | +22.132 | | +7.898 | | -1.487 | -9.167 | -15.261 |
| 23-153-156 | 3 | | 5.410 | -18.745 | +40.075 | | +22.132 | | +7.898 | | -1.487 | -9.167 | -15.261 |
| 139-173-416 | 3 | | 5.363 | -18.832 | +39.085 | | +21.164 | | +6.992 | | -2.238 | -9.655 | -15.493 |
| 224-416 | 2 | | 5.363 | -18.832 | +39.085 | | +21.164 | | +6.992 | | -2.238 | -9.655 | -15.493 |
| 227-229 | 2 | | 5.421 | -17.834 | +39.557 | | +21.902 | | +7.891 | | -1.272 | -8.666 | -14.497 |
| 227-229-353 | 3 | | 5.421 | -17.834 | +39.557 | | +21.902 | | +7.891 | | -1.272 | -8.666 | -14.497 |
| 416 | 1 | | 5.363 | -18.832 | +39.085 | | +21.164 | | +6.992 | | -2.238 | -9.655 | -15.493 |

**Supplementary Table 13-B:** The effect of amino acid changes on the overall pI and charge estimates of *L. plantarum* GAD pathway proteins.

| ***L. plantarum* GadB Protein 469 aa** | | | **Neutral** | **Estimated Charge Over pH Range** | | | | | |
| --- | --- | --- | --- | --- | --- | --- | --- | --- | --- |
| **Residue no on the protein** | **Number of Replacement Changes** | **Estimated pI** | **pH 7.00** | **pH 4.00** | **pH 4.50** | **pH 5.00** | **pH 5.50** | **pH 6.00** | **pH 6.50** |
| No replacement changes | 0 | **5.577** | **-18.399** | +41.39 | +24.694 | +11.014 | +1.347 | -7.268 | -14.35 |
| 180-228 | 2 | 5.577 | -18.398 | +41.39 | +24.694 | +11.014 | +1.347 | -7.268 | -14.35 |
| 291 | 1 | 5.577 | -18.398 | +41.39 | +24.694 | +11.014 | +1.347 | -7.268 | -14.35 |
| 142 | 1 | 5.520 | -19.399 | +40.39 | +23.694 | +10.014 | +0.347 | -8.268 | -15.35 |
| 18-167-182-195-199-235-238 | 7 | 5.593 | -17.486 | +41.4 | +24.726 | +11.109 | +1.596 | -6.756 | -13.582 |
| 33-167-182-195-199-235-238-265-318 | 9 | 5.592 | -17.487 | +40.871 | +24.464 | +11.008 | +1.562 | -6.767 | -13.586 |
| 255-385 | 2 | 5.577 | **-18.399** | +41.39 | +24.694 | +11.014 | +1.347 | -7.268 | -14.35 |
| 180 | 1 | 5.577 | **-18.399** | +41.39 | +24.694 | +11.014 | +1.347 | -7.268 | -14.35 |
| 369 | 1 | 5.577 | **-18.399** | +41.39 | +24.694 | +11.014 | +1.347 | -7.268 | -14.35 |
| 370-451 | 2 | 5.617 | -18.312 | +42.379 | +25.662 | +11.919 | +2.098 | -6.779 | -14.118 |
| 180-301-340 | 3 | 5.634 | -17.4 | +42.39 | +25.694 | +12.014 | +2.347 | -6.268 | -13.351 |
| 341-385-451 | 3 | 5.619 | -18.31 | +42.589 | +25.872 | +12.038 | +2.146 | -6.763 | -14.113 |
| 341-451 | 2 | 5.671 | -17.313 | +42.851 | +26.4 | +12.818 | +3.064 | -5.79 | -13.122 |
| 18-451 | 2 | 5.617 | -18.312 | +42.379 | +25.662 | +11.919 | +2.098 | -6.779 | -14.118 |
| 167-182-195-199-235-238 | 6 | 5.593 | -17.486 | +41.4 | +24.726 | +11.109 | +1.596 | -6.756 | -13.582 |
| 14-318-451 | 3 | 5.726 | -16.316 | +43.641 | +27.191 | +13.699 | +4.016 | -4.807 | -12.128 |
| 167-182-195-199-235-238-318 | 7 | 5.593 | -17.486 | +41.4 | +24.726 | +11.109 | +1.596 | -6.756 | -13.582 |
| 191-451 | 2 | 5.617 | -18.312 | +42.379 | +25.662 | +11.919 | +2.098 | -6.779 | -14.118 |
| 220-451 | 2 | 5.563 | -19.31 | +41.908 | +24.924 | +11.02 | +1.133 | -7.768 | -15.115 |
| 451 | 1 | 5.617 | -18.312 | +42.379 | +25.662 | +11.919 | +2.098 | -6.779 | -14.118 |
| 140-194-195-225-235 | 5 | 5.537 | -18.485 | +40.929 | +23.988 | +10.21 | +0.63 | -7.745 | -14.579 |
| 385-446-451 | 3 | 5.617 | -18.312 | +42.379 | +25.662 | +11.919 | +2.098 | -6.779 | -14.118 |
| 114-140-194-195-225 | 5 | 5.577 | -18.399 | +41.39 | +24.694 | +11.014 | +1.347 | -7.268 | -14.35 |
| 284-317-451 | 3 | 5.617 | -18.312 | +42.379 | +25.662 | +11.919 | +2.098 | -6.779 | -14.118 |
| 220-341-451 | 3 | 5.671 | -17.313 | +42.851 | +26.4 | +12.818 | +3.064 | -5.79 | -13.122 |

(cont. on next page)

**Supplementary Table 13-B:** (cont.)

| ***L. plantarum* GadB Protein 469 aa (cont.)** | | | **Neutral** | **Estimated Charge Over pH Range** | | | | | |
| --- | --- | --- | --- | --- | --- | --- | --- | --- | --- |
| **Residue no on the protein** | **Number of Replacement Changes** | **Estimated pI** | **pH 7.00** | **pH 4.00** | **pH 4.50** | **pH 5.00** | **pH 5.50** | **pH 6.00** | **pH 6.50** |
| 180-312 | 2 | 5.634 | -17.4 | +42.39 | +25.694 | +12.014 | +2.347 | -6.268 | -13.351 |
| 172-220-451 | 3 | 5.563 | -19.31 | +41.908 | +24.924 | +11.02 | +1.133 | -7.768 | -15.115 |
| 385 | 1 | 5.577 | -18.399 | +41.39 | +24.694 | +11.014 | +1.347 | -7.268 | -14.35 |
| ***L. plantarum* YjeM Protein 494 aa** | | | | | | | | | |
| No replacement changes | 0 | 9.519 | +13.81 | +28.244 | +23.991 | +20.348 | +17.973 | +16.189 | +14.772 |
| 127 | 1 | 9.519 | +13.81 | +28.244 | +23.991 | +20.348 | +17.973 | +16.189 | +14.772 |
| 134 | 1 | 9.519 | +13.81 | +28.244 | +23.991 | +20.348 | +17.973 | +16.189 | +14.772 |
| 137 | 1 | 9.519 | +13.81 | +28.244 | +23.991 | +20.348 | +17.973 | +16.189 | +14.772 |
| 137-434 | 2 | 9.519 | +13.81 | +28.244 | +23.991 | +20.348 | +17.973 | +16.189 | +14.772 |
| 198 | 1 | 9.519 | +13.81 | +28.244 | +23.991 | +20.348 | +17.973 | +16.189 | +14.772 |
| 299 | **1** | 9.519 | +13.81 | +28.244 | +23.991 | +20.348 | +17.973 | +16.189 | +14.772 |
| 300 | 1 | 9.519 | +13.81 | +28.244 | +23.991 | +20.348 | +17.973 | +16.189 | +14.772 |
| 300-463 | 2 | 9.568 | +14.81 | +29.244 | +24.991 | +21.348 | +18.973 | +17.189 | +15.772 |
| 308-462 | 2 | 9.479 | +12.811 | +27.244 | +22.991 | +19.348 | +16.973 | +15.189 | +13.772 |
| 308-341-462 | 3 | 9.479 | +12.811 | +27.244 | +22.991 | +19.348 | +16.973 | +15.189 | +13.772 |
| 359 | 1 | 9.532 | +13.811 | +28.244 | +23.991 | +20.348 | +17.973 | +16.189 | +14.772 |
| 413 | 1 | 9.519 | +13.81 | +28.244 | +23.991 | +20.348 | +17.973 | +16.189 | +14.772 |
| 434 | 1 | 9.519 | +13.81 | +28.244 | +23.991 | +20.348 | +17.973 | +16.189 | +14.772 |
| 480 | **1** | 9.519 | +13.81 | +28.244 | +23.991 | +20.348 | +17.973 | +16.189 | +14.772 |

**Supplementary Table 14:** PLP/L-Glutamate binding and other importanat residues on the *L. brevis* and *L. plantarum* GAD enzymes.

| ***L. brevis***  **Gad1** | ***L. brevis***  **Gad2** | ***L. plantarum* GadB** | **Bond Information** |
| --- | --- | --- | --- |
| T66 | T64 | T64 | Hyrogen bond with L-Glu |
| F67 | F65 | F65 | Conserved and critical for catalytic activity |
| C68 | C66 | C66 | Hydrogen bond with L-Glu |
| N87 | N85 | N85 | Conserved and critical for catalytic activity |
| S132 | S126 | S126 | Interaction with PLP |
| S133 | S127 | S127 | Hyrogen bond with PLP |
| Q175 | Q166 | Q166 | Hydrogen bond with L-Glu |
| V177 | C168 | C168 | Hydrophobic interaction with pyridine ring of PLP |
| I220 | I211 | I211 | Hydrophobic interaction with pyridine ring of PLP |
| T224 | T215 | T215 | Necessary for the enzyme activity |
| A257 | A248 | A249 | Hydrophobic interaction with pyridine ring of PLP |
| S285 | S276 | S277 | Hydrogen bond with PLP |
| H287 | H278 | H279 | Salt bridge with L-Glu, Hydrogen bond with PLP |
| K288 | K279 | K280 | Salt bridge with L-Glu, PLP is covalently attached to the catalytic K279(Gad2) |
| F331 | F320 | F321 | Conserved and critical for catalytic activity |
| S332 | S321 | S322 | Hydrogen bond with L-Glu |
| R433 | R422 | R423 | Salt bridge with L-Glu |
| **Flexible Loop** | | | |
| Y319 | Y308 | Y309 | It covers the active site and provides a catalytic environment for GABA production. |
| L320 | L309 | L310 |  |
| G321 | G310 | G311 |  |
| K322 | G311 | G312 |  |
| T323 | E312 | E313 |  |

**Supplementary Table 15:** Codon bias index (CBI) and Codon adaptation (CAI) index estimates for *L. brevis*/*L. plantarum* GAD system genes

| **Organisms** | **Gene** | **CBI** | **Scaled Chi-square, SChi2** | **CAI** |
| --- | --- | --- | --- | --- |
| *L. brevis* | | | | |
|  | ***gad1*** | 0.39 | 0.36 | 0.83 |
|  | ***gad2*** | 0.30 | 0.27 | 0.77 |
|  | ***gadC*** | 0.33 | 0.28 | 0.79 |
|  | ***gadR*** | 0.45 | 0.39 | 0.80 |
|  |  |  |  |  |
| *L. plantarum* | | | | |
|  | ***gadB*** | 0.29 | 0.26 | 0.79 |
|  | ***yjeM*** | 0.33 | 0.28 | 0.79 |
|  |  |  |  |  |

**Supplementary Table 16-A:** Interspecific *L. brevis gad1* sequence comparisons with *gad*(glutamate decarboxylase) sequences from different bacterial species.

|  | **Fixed** | **Polymorphic** | **P** | **NI** | **Alpha** | **DoS** |
| --- | --- | --- | --- | --- | --- | --- |
| ***L. oris gad*** | | | | | | |
| Synonymous | 244 | 40 | 0,000*** | 0.12 | 0.88 | 0.44 |
| Nonsynonymous | 327 | 6 |  |  |  |  |
| ***L. antri gad*** | | | | | | |
| Synonymous | 255 | 40 | 0,000*** | 0.12 | 0.88 | 0.43 |
| Nonsynonymous | 323 | 6 |  |  |  |  |
| ***L. reuteri gad*** | | | | | | |
| Synonymous | 253 | 40 | 0,000*** | 0.12 | 0.88 | 0.43 |
| Nonsynonymous | 324 | 6 |  |  |  |  |
| ***L. fermentum gad*** | | | | | | |
| Synonymous | 270 | 40 | 0,000*** | 0.13 | 0.87 | 0.42 |
| Nonsynonymous | 324 | 6 |  |  |  |  |
| ***L. gastricus gad*** | | | | | | |
| Synonymous | 249 | 40 | 0,000*** | 0.11 | 0.89 | 0.44 |
| Nonsynonymous | 330 | 6 |  |  |  |  |
| ***P. suebicus gad*** | | | | | | |
| Synonymous | 257 | 40 | 0,000*** | 0.12 | 0.88 | 0.42 |
| Nonsynonymous | 320 | 6 |  |  |  |  |
| ***L. lactis gad*** | | | | | | |
| Synonymous | 243 | 40 | 0,000*** | 0.11 | 0.89 | 0.44 |
| Nonsynonymous | 322 | 6 |  |  |  |  |
| ***E. faecium gad*** | | | | | | |
| Synonymous | 257 | 40 | 0,000*** | 0.12 | 0.88 | 0.43 |
| Nonsynonymous | 329 | 6 |  |  |  |  |
| ***L.brevis gad2*** | | | | | | |
| Synonymous | 275 | 40 | 0,000*** | 0.13 | 0.87 | 0.41 |
| Nonsynonymous | 322 | 6 |  |  |  |  |
| ***L. herbarum gad*** | | | | | | |
| Synonymous | 260 | 40 | 0,000*** | 0.13 | 0.87 | 0.41 |
| Nonsynonymous | 310 | 6 |  |  |  |  |
| ***L. paraplantarum gad*** | | | | | | |
| Synonymous | 270 | 40 | 0,000*** | 0.13 | 0.87 | 0.41 |
| Nonsynonymous | 323 | 6 |  |  |  |  |
| ***L. argentoratensis gad*** | | | | | | |
| Synonymous | 266 | 40 | 0,000*** | 0.12 | 0.88 | 0.42 |
| Nonsynonymous | 321 | 6 |  |  |  |  |
| ***L.plantarum gadB*** | | | | | | |
| Synonymous | 272 | 40 | 0,000*** | 0.13 | 0.87 | 0.41 |
| Nonsynonymous | 317 | 6 |  |  |  |  |
| ***C. futsaii gad*** | | | | | | |
| Synonymous | 272 | 40 | 0,000*** | 0.13 | 0.87 | 0.41 |
| Nonsynonymous | 318 | 6 |  |  |  |  |
| ***C. nuruk gadi*** | | | | | | |
| Synonymous | 242 | 40 | 0,000*** | 0.11 | 0.89 | 0.44 |
| Nonsynonymous | 324 | 6 |  |  |  |  |
| ***S. paracollinoides gad*** | | | | | | |
| Synonymous | 258 | 40 | 0,000*** | 0.12 | 0.88 | 0.44 |
| Nonsynonymous | 337 | 6 |  |  |  |  |
| ***F. rossiae gad*** | | | | | | |
| Synonymous | 241 | 40 | 0,001** | 0.27 | 0.73 | 0.23 |
| Nonsynonymous | 135 | 6 |  |  |  |  |
| ***L. buchneri gad*** | | | | | | |
| Synonymous | 260 | 40 | 0,002** | 0.27 | 0.73 | 0.23 |
| Nonsynonymous | 147 | 6 |  |  |  |  |

(cont. on next page)

**Supplementary Table 16-A:** (cont.)

|  | **Fixed** | **Polymorphic** | **P** | **NI** | **Alpha** | **DoS** |
| --- | --- | --- | --- | --- | --- | --- |
| ***L. paracasei gad*** | | | | | | |
| Synonymous | 259 | 40 | 0,001*** | 0.26 | 0.74 | 0.23 |
| Nonsynonymous | 149 | 6 |  |  |  |  |
| ***L. parakefiri gad*** | | | | | | |
| Synonymous | 250 | 40 | 0,003** | 0.29 | 0.71 | 0.21 |
| Nonsynonymous | 131 | 6 |  |  |  |  |
| ***L. curvatus gad*** | | | | | | |
| Synonymous | 229 | 40 | 0,000*** | 0.15 | 0.85 | 0.31 |
| Nonsynonymous | 154 | 4 |  |  |  |  |
| ***L. rennini gad*** | | | | | | |
| Synonymous | 222 | 40 | 0,0003*** | 0.23 | 0.77 | 0.26 |
| Nonsynonymous | 144 | 6 |  |  |  |  |
| ***L. coleohominis gad*** | | | | | | |
| Synonymous | 219 | 38 | 0,000*** | 0.09 | 0.91 | 0.40 |
| Nonsynonymous | 194 | 3 |  |  |  |  |
| ***L. senmaizukei gad*** | | | | | | |
| Synonymous | 267 | 41 | 0,015* | 0.35 | 0.65 | 0.17 |
| Nonsynonymous | 111 | 6 |  |  |  |  |
| ***L. tangyuanensis gad*** | | | | | | |
| Synonymous | 263 | 40 | 0,022* | 0.36 | 0.64 | 0.16 |
| Nonsynonymous | 109 | 6 |  |  |  |  |
| ***L. spicheri gad*** | | | | | | |
| Synonymous | 223 | 40 | 0,412 | 0.64 | 0.36 | 0.06 |
| Nonsynonymous | 52 | 6 |  |  |  |  |
| ***L. angrenensis gad*** | | | | | | |
| Synonymous | 232 | 41 | 0,320 | 0.62 | 0.38 | 0.06 |
| Nonsynonymous | 55 | 6 |  |  |  |  |
| ***L. cerevisiae gad*** | | | | | | |
| Synonymous | 230 | 41 | 0,177 | 0.52 | 0.48 | 0.09 |
| Nonsynonymous | 65 | 6 |  |  |  |  |
| ***L. zymae gad*** | | | | | | |
| Synonymous | 0 | 41 | - | - | - | - |
| Nonsynonymous | 0 | 6 |  |  |  |  |
| ***L. sakei gad*** | | | | | | |
| Synonymous | 0 | 41 | 0,145 | 0.00 | 1.00 | 0.87 |
| Nonsynonymous | 1 | 6 |  |  |  |  |
| ***S. thermophilus gad*** | | | | | | |
| Synonymous | 269 | 41 | 0,000*** | 0.10 | 0.90 | 0.47 |
| Nonsynonymous | 397 | 6 |  |  |  |  |
| ***E.coli gad-alpha*** | | | | | | |
| Synonymous | 269 | 39 | 0,000*** | 0.09 | 0.91 | 0.49 |
| Nonsynonymous | 439 | 6 |  |  |  |  |
| ***E.coli gad-beta*** | | | | | | |
| Synonymous | 271 | 40 | 0,000*** | 0.08 | 0.92 | 0.49 |
| Nonsynonymous | 440 | 5 |  |  |  |  |
|  |  |  |  |  |  |  |

Note: Number of nonsynonymous and synonymous substitutions for fixed differences and polymorphisms between species different strains *gad1* genes of *L. brevis* compared with the sequences of other LABs *gad* genes coding region of species was examined. NI: Neutrality Index , P: Fisher's exact test. P-value (two tailed), DoS: Direction of selection.

* 0.01 < P < 0.05, ** 0.001 < P < 0.01, *** P < 0.001

**Supplementary Table 16-B:** Interspecific *L. brevis gad2* sequence comparisons with *gad*(glutamate decarboxylase) sequences from different bacterial species.

|  | **Fixed** | **Polymorphic** | **P** | **NI** | **Alpha** | **DoS** |
| --- | --- | --- | --- | --- | --- | --- |
| ***L. oris gad*** | | | | | | |
| Synonymous | 262 | 55 | 0,0003*** | 0.34 | 0.66 | 0.23 |
| Nonsynonymous | 196 | 14 |  |  |  |  |
| ***L. antri gad*** | | | | | | |
| Synonymous | 269 | 55 | 0,001*** | 0.36 | 0.64 | 0.21 |
| Nonsynonymous | 189 | 14 |  |  |  |  |
| ***L. reuteri gad*** | | | | | | |
| Synonymous | 278 | 55 | 0,0002*** | 0.34 | 0.66 | 0.23 |
| Nonsynonymous | 210 | 14 |  |  |  |  |
| ***L. fermentum gad*** | | | | | | |
| Synonymous | 262 | 55 | 0,001*** | 0.35 | 0.65 | 0.22 |
| Nonsynonymous | 190 | 14 |  |  |  |  |
| ***L. gastricus gad*** | | | | | | |
| Synonymous | 252 | 55 | 0,001*** | 0.35 | 0.65 | 0.22 |
| Nonsynonymous | 182 | 14 |  |  |  |  |
| ***P. suebicus gad*** | | | | | | |
| Synonymous | 261 | 55 | 0,001*** | 0.37 | 0.63 | 0.21 |
| Nonsynonymous | 180 | 14 |  |  |  |  |
| ***L. lactis gad*** | | | | | | |
| Synonymous | 261 | 55 | 0,001** | 0.38 | 0.62 | 0.20 |
| Nonsynonymous | 177 | 14 |  |  |  |  |
| ***E. faecium gad*** | | | | | | |
| Synonymous | 255 | 55 | 0,001** | 0.38 | 0.62 | 0.20 |
| Nonsynonymous | 171 | 14 |  |  |  |  |
| ***L. herbarum gad*** | | | | | | |
| Synonymous | 262 | 55 | 0,236 | 0.67 | 0.33 | 0.07 |
| Nonsynonymous | 99 | 14 |  |  |  |  |
| ***L. paraplantarum gad*** | | | | | | |
| Synonymous | 251 | 55 | 0,186 | 0.65 | 0.35 | 0.08 |
| Nonsynonymous | 98 | 14 |  |  |  |  |
| ***L. argentoratensis gad*** | | | | | | |
| Synonymous | 262 | 55 | 0,185 | 0.64 | 0.36 | 0.08 |
| Nonsynonymous | 104 | 14 |  |  |  |  |
| ***L.plantarum gadB*** | | | | | | |
| Synonymous | 263 | 55 | 0,235 | 0.66 | 0.34 | 0.07 |
| Nonsynonymous | 101 | 14 |  |  |  |  |
| ***C. futsaii gad*** | | | | | | |
| Synonymous | 265 | 55 | 0,235 | 0.66 | 0.34 | 0.08 |
| Nonsynonymous | 102 | 14 |  |  |  |  |
| ***C. nuruki gad*** | | | | | | |
| Synonymous | 274 | 55 | 0,0003*** | 0.35 | 0.65 | 0.22 |
| Nonsynonymous | 201 | 14 |  |  |  |  |
| ***S. paracollinoides gad*** | | | | | | |
| Synonymous | 240 | 55 | 0,001*** | 0.36 | 0.64 | 0.21 |
| Nonsynonymous | 170 | 14 |  |  |  |  |
| ***F. rossiae gad*** | | | | | | |
| Synonymous | 270 | 55 | 0,000*** | 0.22 | 0.78 | 0.31 |
| Nonsynonymous | 290 | 13 |  |  |  |  |
| ***L. buchneri gad*** | | | | | | |
| Synonymous | 273 | 55 | 0,000*** | 0.21 | 0.79 | 0.34 |
| Nonsynonymous | 329 | 14 |  |  |  |  |
| ***L. paracasei gad*** | | | | | | |
| Synonymous | 273 | 55 | 0,000*** | 0.21 | 0.79 | 0.34 |
| Nonsynonymous | 329 | 14 |  |  |  |  |

(cont. on next page)

**Supplementary Table 16-B:** (cont.)

|  | **Fixed** | **Polymorphic** | **P** | **NI** | **Alpha** | **DoS** |
| --- | --- | --- | --- | --- | --- | --- |
| ***L. parakefiri gad*** | | | | | | |
| Synonymous | 284 | 55 | 0,000*** | 0.23 | 0.77 | 0.32 |
| Nonsynonymous | 314 | 14 |  |  |  |  |
| ***L. curvatus gad*** | | | | | | |
| Synonymous | 257 | 53 | 0,000*** | 0.23 | 0.77 | 0.32 |
| Nonsynonymous | 291 | 14 |  |  |  |  |
| ***L. rennini gad*** | | | | | | |
| Synonymous | 266 | 54 | 0,000*** | 0.22 | 0.78 | 0.33 |
| Nonsynonymous | 313 | 14 |  |  |  |  |
| ***L. coleohominis gad*** | | | | | | |
| Synonymous | 239 | 51 | 0,000*** | 0.16 | 0.84 | 0.38 |
| Nonsynonymous | 285 | 10 |  |  |  |  |
| ***L. senmaizukei gad*** | | | | | | |
| Synonymous | 273 | 55 | 0,000*** | 0.22 | 0.78 | 0.33 |
| Nonsynonymous | 316 | 14 |  |  |  |  |
| ***L. tangyuanensis gad*** | | | | | | |
| Synonymous | 280 | 54 | 0,000*** | 0.24 | 0.74 | 0.32 |
| Nonsynonymous | 308 | 14 |  |  |  |  |
| ***L. spicheri gad*** | | | | | | |
| Synonymous | 275 | 54 | 0,000*** | 0.23 | 0.77 | 0.33 |
| Nonsynonymous | 315 | 14 |  |  |  |  |
| ***L. angrenensis gad*** | | | | | | |
| Synonymous | 280 | 55 | 0,000*** | 0.22 | 0.78 | 0.33 |
| Nonsynonymous | 320 | 14 |  |  |  |  |
| ***L. cerevisiae gad*** | | | | | | |
| Synonymous | 279 | 55 | 0,000*** | 0.22 | 0.78 | 0.34 |
| Nonsynonymous | 330 | 14 |  |  |  |  |
| ***L. brevis gad1*** | | | | | | |
| Synonymous | 264 | 55 | 0,000*** | 0.20 | 0.80 | 0.35 |
| Nonsynonymous | 312 | 13 |  |  |  |  |
| ***L. zymae gad*** | | | | | | |
| Synonymous | 262 | 55 | 0,000*** | 0.21 | 0.79 | 0.35 |
| Nonsynonymous | 318 | 14 |  |  |  |  |
| ***L. sakei gad*** | | | | | | |
| Synonymous | 261 | 55 | 0,000*** | 0.21 | 0.79 | 0.35 |
| Nonsynonymous | 319 | 14 |  |  |  |  |
| ***S. thermophilus gad*** | | | | | | |
| Synonymous | 252 | 55 | 0,000*** | 0.18 | 0.82 | 0.38 |
| Nonsynonymous | 353 | 14 |  |  |  |  |
| ***E. coli gad-alpha*** | | | | | | |
| Synonymous | 249 | 54 | 0,000*** | 0.16 | 0.84 | 0.40 |
| Nonsynonymous | 376 | 13 |  |  |  |  |
| ***E. coli gad-beta*** | | | | | | |
| Synonymous | 251 | 55 | 0,000*** | 0.15 | 0.85 | 0.41 |
| Nonsynonymous | 385 | 13 |  |  |  |  |
|  |  |  |  |  |  |  |

Note: Number of nonsynonymous and synonymous substitutions for fixed differences and polymorphisms between species different strains *gad2* genes of *L. brevis* compared with the sequences of other LABs *gad* genes coding region of species was examined. NI: Neutrality Index , P: Fisher's exact test. P-value (two tailed), DoS: Direction of selection.

* 0.01 < P < 0.05, ** 0.001 < P < 0.01, *** P < 0.001

**Supplementary Table 16-C:** Interspecific *L. plantarum gadB* sequence comparisons with *gad*(glutamate decarboxylase) sequences from different bacterial species.

|  | **Fixed** | **Polymorphic** | **P** | **NI** | **Alpha** | **DoS** |
| --- | --- | --- | --- | --- | --- | --- |
| ***L. oris gad*** | | | | | | |
| Synonymous | 257 | 82 | 0,010** | 0.57 | 0.43 | 0.13 |
| Nonsynonymous | 209 | 38 |  |  |  |  |
| ***L. antri gad*** | | | | | | |
| Synonymous | 251 | 82 | 0,010** | 0.57 | 0.43 | 0.13 |
| Nonsynonymous | 204 | 38 |  |  |  |  |
| ***L. reuteri gad*** | | | | | | |
| Synonymous | 271 | 82 | 0,009** | 0.56 | 0.44 | 0.13 |
| Nonsynonymous | 217 | 37 |  |  |  |  |
| ***L. fermentum gad*** | | | | | | |
| Synonymous | 253 | 82 | 0,021* | 0.60 | 0.40 | 0.12 |
| Nonsynonymous | 191 | 37 |  |  |  |  |
| ***L. gastricus gad*** | | | | | | |
| Synonymous | 240 | 82 | 0,007** | 0.55 | 0.45 | 0.14 |
| Nonsynonymous | 196 | 37 |  |  |  |  |
| ***P. suebicus gad*** | | | | | | |
| Synonymous | 236 | 82 | 0,011* | 0.57 | 0.43 | 0.13 |
| Nonsynonymous | 186 | 37 |  |  |  |  |
| ***L. lactis gad*** | | | | | | |
| Synonymous | 252 | 82 | 0,028* | 0.62 | 0.38 | 0.11 |
| Nonsynonymous | 189 | 38 |  |  |  |  |
| ***E. faecium gad*** | | | | | | |
| Synonymous | 243 | 82 | 0,045* | 0.64 | 0.36 | 0.10 |
| Nonsynonymous | 175 | 38 |  |  |  |  |
| ***L.brevis gad2*** | | | | | | |
| Synonymous | 253 | 82 | 0,644 | 1.14 | -0.14 | -0.03 |
| Nonsynonymous | 103 | 38 |  |  |  |  |
| ***L. herbarum gad*** | | | | | | |
| Synonymous | 215 | 82 | 0,181 | 1.39 | -0.39 | -0.07 |
| Nonsynonymous | 72 | 38 |  |  |  |  |
| ***L. paraplantarum gad*** | | | | | | |
| Synonymous | 191 | 82 | 0,000*** | 4.02 | -3.02 | -0.21 |
| Nonsynonymous | 22 | 38 |  |  |  |  |
| ***L. argentoratensis gad*** | | | | | | |
| Synonymous | 1 | 82 | 0,105 | 0.15 | 0.85 | 0.43 |
| Nonsynonymous | 3 | 38 |  |  |  |  |
| ***C. futsaii gad*** | | | | | | |
| Synonymous | 2 | 82 | 1,000 | 0.93 | 0.07 | 0.02 |
| Nonsynonymous | 1 | 38 |  |  |  |  |
| ***C. nuruki gad*** | | | | | | |
| Synonymous | 253 | 82 | 0,009** | 0.57 | 0.43 | 0.13 |
| Nonsynonymous | 202 | 37 |  |  |  |  |
| ***S. paracollinoides gad*** | | | | | | |
| Synonymous | 243 | 82 | 0,055 | 0.64 | 0.36 | 0.10 |
| Nonsynonymous | 171 | 37 |  |  |  |  |
| ***F. rossiae gad*** | | | | | | |
| Synonymous | 252 | 82 | 0,000*** | 0.38 | 0.62 | 0.23 |
| Nonsynonymous | 296 | 37 |  |  |  |  |
| ***L. buchneri gad*** | | | | | | |
| Synonymous | 274 | 82 | 0,000*** | 0.39 | 0.61 | 0.23 |
| Nonsynonymous | 318 | 37 |  |  |  |  |
| ***L. paracasei gad*** | | | | | | |
| Synonymous | 274 | 82 | 0,000*** | 0.39 | 0.61 | 0.23 |
| Nonsynonymous | 319 | 37 |  |  |  |  |

(cont. on next page)

**Supplementary Table 16-C:** (cont.)

|  | **Fixed** | **Polymorphic** | **P** | **NI** | **Alpha** | **DoS** |
| --- | --- | --- | --- | --- | --- | --- |
| ***L. parakefiri gad*** | | | | | | |
| Synonymous | 267 | 82 | 0,000*** | 0.39 | 0.61 | 0.23 |
| Nonsynonymous | 309 | 37 |  |  |  |  |
| ***L. rennini gad*** | | | | | | |
| Synonymous | 244 | 82 | 0,000*** | 0.34 | 0.66 | 0.26 |
| Nonsynonymous | 326 | 37 |  |  |  |  |
| ***L. coleohominis gad*** | | | | | | |
| Synonymous | 235 | 76 | 0,000*** | 0.38 | 0.62 | 0.23 |
| Nonsynonymous | 278 | 34 |  |  |  |  |
| ***L. senmaizukei gad*** | | | | | | |
| Synonymous | 259 | 81 | 0,000*** | 0.38 | 0.62 | 0.23 |
| Nonsynonymous | 308 | 37 |  |  |  |  |
| ***L. tangyuanensis gad*** | | | | | | |
| Synonymous | 257 | 82 | 0,000*** | 0.39 | 0.61 | 0.23 |
| Nonsynonymous | 298 | 37 |  |  |  |  |
| ***L. spicheri gad*** | | | | | | |
| Synonymous | 259 | 82 | 0,000*** | 0.39 | 0.61 | 0.23 |
| Nonsynonymous | 303 | 37 |  |  |  |  |
| ***L. angrenensis gad*** | | | | | | |
| Synonymous | 243 | 82 | 0,000*** | 0.36 | 0.64 | 0.25 |
| Nonsynonymous | 309 | 37 |  |  |  |  |
| ***L. cerevisiae gad*** | | | | | | |
| Synonymous | 250 | 81 | 0,000*** | 0.36 | 0.64 | 0.24 |
| Nonsynonymous | 315 | 37 |  |  |  |  |
| ***L. brevis gad1*** | | | | | | |
| Synonymous | 253 | 82 | 0,000*** | 0.36 | 0.64 | 0.24 |
| Nonsynonymous | 301 | 35 |  |  |  |  |
| ***L. zymae gad*** | | | | | | |
| Synonymous | 255 | 82 | 0,000*** | 0.37 | 0.63 | 0.24 |
| Nonsynonymous | 309 | 37 |  |  |  |  |
| ***L. sakei gad*** | | | | | | |
| Synonymous | 255 | 82 | 0,000*** | 0.37 | 0.63 | 0.24 |
| Nonsynonymous | 310 | 37 |  |  |  |  |
| ***S. thermophilus gad*** | | | | | | |
| Synonymous | 250 | 82 | 0,000*** | 0.33 | 0.67 | 0.27 |
| Nonsynonymous | 342 | 37 |  |  |  |  |
| ***E. coli gad-alpha*** | | | | | | |
| Synonymous | 244 | 81 | 0,000*** | 0.31 | 0.69 | 0.28 |
| Nonsynonymous | 366 | 38 |  |  |  |  |
| ***E. coli gad-beta*** | | | | | | |
| Synonymous | 250 | 81 | 0,000*** | 0.32 | 0.68 | 0.28 |
| Nonsynonymous | 369 | 38 |  |  |  |  |
|  |  |  |  |  |  |  |

Note: Number of nonsynonymous and synonymous substitutions for fixed differences and polymorphisms between species different strains *gadB* genes of *L. plantarum* compared with the sequences of other LABs *gad* genes coding region of species was examined. NI: Neutrality Index , P: Fisher's exact test. P-value (two tailed), DoS: Direction of selection.

* 0.01 < P < 0.05, ** 0.001 < P < 0.01, *** P < 0.001

**Supplementary Table 16-D:** Interspecific *L. brevis gadC* sequence comparisons with *gadC* or *yjeM* sequences from different bacterial species.

|  | **Fixed** | **Polymorphic** | **P** | **NI** | **Alpha** | **DoS** |
| --- | --- | --- | --- | --- | --- | --- |
| ***L. zymae gadC*** | | | | | | |
| Synonymous | 0 | 52 | - | - | - | - |
| Nonsynonymous | 0 | 7 |  |  |  |  |
| ***L. sakei gadC*** | | | | | | |
| Synonymous | 0 | 52 | - | - | - | - |
| Nonsynonymous | 0 | 7 |  |  |  |  |
| ***L. angrenensis gadC*** | | | | | | |
| Synonymous | 240 | 52 | 0,198 | 0.56 | 0.44 | 0.08 |
| Nonsynonymous | 58 | 7 |  |  |  |  |
| ***L. cerevisiae gadC*** | | | | | | |
| Synonymous | 263 | 52 | 0,151 | 0.53 | 0.47 | 0.08 |
| Nonsynonymous | 67 | 7 |  |  |  |  |
| ***L. spicheri gadC*** | | | | | | |
| Synonymous | 265 | 52 | 0,043* | 0.44 | 0.56 | 0.12 |
| Nonsynonymous | 82 | 7 |  |  |  |  |
| ***L. senmaizukei gadC*** | | | | | | |
| Synonymous | 283 | 52 | 0,002** | 0.31 | 0.69 | 0.19 |
| Nonsynonymous | 124 | 7 |  |  |  |  |
| ***L. tangyuanensis gadC*** | | | | | | |
| Synonymous | 280 | 52 | 0,002** | 0.30 | 0.70 | 0.20 |
| Nonsynonymous | 125 | 7 |  |  |  |  |
| ***F. rossiae gadC*** | | | | | | |
| Synonymous | 284 | 52 | 0,001*** | 0.27 | 0.73 | 0.21 |
| Nonsynonymous | 142 | 7 |  |  |  |  |
| ***L. parakefiri gadC*** | | | | | | |
| Synonymous | 250 | 50 | 0,000*** | 0.19 | 0.81 | 0.28 |
| Nonsynonymous | 159 | 6 |  |  |  |  |
| ***L. buchneri gadC*** | | | | | | |
| Synonymous | 276 | 52 | 0,000*** | 0.23 | 0.77 | 0.25 |
| Nonsynonymous | 165 | 7 |  |  |  |  |
| ***L. rennini gadC*** | | | | | | |
| Synonymous | 255 | 52 | 0,000*** | 0.20 | 0.80 | 0.28 |
| Nonsynonymous | 169 | 7 |  |  |  |  |
| ***L. curvatus gadC*** | | | | | | |
| Synonymous | 259 | 49 | 0,000*** | 0.20 | 0.80 | 0.23 |
| Nonsynonymous | 188 | 7 |  |  |  |  |
| ***L. coleohominis gadC*** | | | | | | |
| Synonymous | 265 | 50 | 0,000*** | 0.17 | 0.83 | 0.33 |
| Nonsynonymous | 221 | 7 |  |  |  |  |
| ***E. coli gadC*** | | | | | | |
| Synonymous | 333 | 52 | 0,000*** | 0.09 | 0.91 | 0.46 |
| Nonsynonymous | 429 | 6 |  |  |  |  |
| ***L. lactis gadC*** | | | | | | |
| Synonymous | 312 | 52 | 0,000*** | 0.09 | 0.91 | 0.46 |
| Nonsynonymous | 399 | 6 |  |  |  |  |
| ***E. faecium gadC*** | | | | | | |
| Synonymous | 280 | 51 | 0,000*** | 0.08 | 0.92 | 0.49 |
| Nonsynonymous | 404 | 6 |  |  |  |  |
| ***L. reuteri gadC*** | | | | | | |
| Synonymous | 312 | 52 | 0,000*** | 0.08 | 0.92 | 0.47 |
| Nonsynonymous | 427 | 6 |  |  |  |  |
| ***L. oris gadC*** | | | | | | |
| Synonymous | 296 | 52 | 0,000*** | 0.09 | 0.91 | 0.47 |
| Nonsynonymous | 400 | 6 |  |  |  |  |
|  |  |  |  |  |  |  |

(cont. on next page)

**Supplementary Table 16-D:** (cont.)

|  | **Fixed** | **Polymorphic** | **P** | **NI** | **Alpha** | **DoS** |
| --- | --- | --- | --- | --- | --- | --- |
| ***L. antri gadC*** | | | | | | |
| Synonymous | 290 | 52 | 0,000*** | 0.08 | 0.92 | 0.47 |
| Nonsynonymous | 397 | 6 |  |  |  |  |
| ***S. thermophilus gadC*** | | | | | | |
| Synonymous | 280 | 52 | 0,000*** | 0.08 | 0.92 | 0.52 |
| Nonsynonymous | 497 | 7 |  |  |  |  |
| ***C. nuruki yjeM*** | | | | | | |
| Synonymous | 284 | 45 | 0,000*** | 0.08 | 0.92 | 0.52 |
| Nonsynonymous | 538 | 7 |  |  |  |  |
| ***C. futsaii yjeM*** | | | | | | |
| Synonymous | 274 | 44 | 0,000*** | 0.08 | 0.92 | 0.52 |
| Nonsynonymous | 530 | 7 |  |  |  |  |
| ***L. plantarum yjeM*** | | | | | | |
| Synonymous | 292 | 47 | 0,000*** | 0.09 | 0.91 | 0.50 |
| Nonsynonymous | 506 | 7 |  |  |  |  |
| ***L. paraplantarum yjeM*** | | | | | | |
| Synonymous | 279 | 45 | 0,000*** | 0.09 | 0.91 | 0.51 |
| Nonsynonymous | 507 | 7 |  |  |  |  |
| ***L. argentoratensis yjeM*** | | | | | | |
| Synonymous | 295 | 47 | 0,000*** | 0.09 | 0.91 | 0.51 |
| Nonsynonymous | 513 | 7 |  |  |  |  |
| ***S. paracollinoides yjeM*** | | | | | | |
| Synonymous | 284 | 46 | 0,000*** | 0.07 | 0.93 | 0.53 |
| Nonsynonymous | 514 | 6 |  |  |  |  |
| ***L. fermentum yjeM*** | | | | | | |
| Synonymous | 284 | 46 | 0,000*** | 0.08 | 0.92 | 0.51 |
| Nonsynonymous | 514 | 7 |  |  |  |  |
| ***L. gastricus yjeM*** | | | | | | |
| Synonymous | 260 | 45 | 0,000*** | 0.08 | 0.92 | 0.53 |
| Nonsynonymous | 517 | 7 |  |  |  |  |
|  | | | | | | |

Note: Number of nonsynonymous and synonymous substitutions for fixed differences and polymorphisms between species different strains *gadC* genes of *L. brevis* compared with the sequences of other LABs *gadC* or *yjeM* genes coding region of species was examined. NI: Neutrality Index , P: Fisher's exact test. P-value (two tailed), DoS: Direction of selection.

* 0.01 < P < 0.05, ** 0.001 < P < 0.01, *** P < 0.001

**Supplementary Table 16-E:** Interspecific *L. plantarum yjeM* sequence comparisons with *yjeM* sequences from different bacterial species.

|  | **Fixed** | **Polymorphic** | **P** | **NI** | **Alpha** | **DoS** |
| --- | --- | --- | --- | --- | --- | --- |
| ***C. nuruki yjeM*** | | | | | | |
| Synonymous | 279 | 51 | 0,000*** | 0.22 | 0.78 | 0.34 |
| Nonsynonymous | 330 | 13 |  |  |  |  |
| ***C. futsaii yjeM*** | | | | | | |
| Synonymous | 273 | 51 | 0,000*** | 0.20 | 0.80 | 0.36 |
| Nonsynonymous | 347 | 13 |  |  |  |  |
| ***L. paraplantarum yjeM*** | | | | | | |
| Synonymous | 125 | 51 | 0,001** | 4.9 | -3.9 | -0.16 |
| Nonsynonymous | 7 | 14 |  |  |  |  |
| ***L. argentoratensis yjeM*** | | | | | | |
| Synonymous | 0 | 51 | - | - | - | - |
| Nonsynonymous | 0 | 14 |  |  |  |  |
| ***S. paracollinoides yjeM*** | | | | | | |
| Synonymous | 268 | 51 | 0,000*** | 0.20 | 0.80 | 0.36 |
| Nonsynonymous | 362 | 14 |  |  |  |  |
| ***L. fermentum yjeM*** | | | | | | |
| Synonymous | 287 | 50 | 0,000*** | 0.20 | 0.80 | 0.37 |
| Nonsynonymous | 385 | 13 |  |  |  |  |
| ***L. gastricus yjeM*** | | | | | | |
| Synonymous | 293 | 51 | 0,000*** | 0.18 | 0.82 | 0.38 |
| Nonsynonymous | 418 | 13 |  |  |  |  |
|  | | | | | | |

Note: Number of nonsynonymous and synonymous substitutions for fixed differences and polymorphisms between species different strains *yjeM* genes of *L. plantarum* compared with the sequences of other LABs *yjeM* genes coding region of species was examined. NI: Neutrality Index , P: Fisher's exact test. P-value (two tailed), DoS: Direction of selection.

** 0.001 < P < 0.01, *** P < 0.001

| **Name** | **length** | **CAI** | **%GC** | **%GC1** | **%GC2** | **%GC3** |
| --- | --- | --- | --- | --- | --- | --- |
| *Levilactobacillus brevis gad2* | 1407 | 0.767 | 48.2 | 54.4 | 36.2 | 53.9 |
| *Levilactobacillus brevis gad1* | 1410 | 0.829 | 45.7 | 50.4 | 36.8 | 50 |
| *Levilactobacillus zymae gad* | 1440 | 0.827 | 45.2 | 50.4 | 36.2 | 49 |
| *Levilactobacillus senmaizukei gad* | 1437 | 0.743 | 45.9 | 50.9 | 36.1 | 50.5 |
| *Levilactobacillus tangyuanensis gad* | 1407 | 0.742 | 45.7 | 51 | 36.5 | 49.7 |
| *Levilactobacillus angrenensis gad* | 1446 | 0.799 | 45.2 | 49 | 35.3 | 51.2 |
| *Levilactobacillus cerevisiae gad* | 1446 | 0.824 | 42.1 | 50.2 | 34.4 | 41.7 |
| *Levilactobacillus spicheri gad* | 1428 | 0.798 | 46.4 | 48.9 | 35.9 | 54.4 |
| *Lactiplantibacillus plantarum gadB* | 1410 | 0.792 | 47.3 | 58.1 | 35.3 | 48.5 |
| *Lactiplantibacillus argentoratensis gad* | 1410 | 0.788 | 46.9 | 57.2 | 35.7 | 47.7 |
| *Lactiplantibacillus paraplantarum gad* | 1410 | 0.787 | 45.7 | 56 | 36 | 45.3 |
| *Lactiplantibacillus herbarum gad* | 1404 | 0.767 | 47.4 | 56.8 | 34.8 | 50.6 |
| *Limosilactobacillus reuteri gad* | 1407 | 0.792 | 35.6 | 49.3 | 34.3 | 23.2 |
| *Limosilactobacillus fermentum gad* | 1404 | 0.726 | 55.3 | 55.6 | 33.3 | 77.1 |
| *Limosilactobacillus oris gad* | 1407 | 0.793 | 49.1 | 53.5 | 33.9 | 59.9 |
| *Limosilactobacillus antri gad* | 1407 | 0.797 | 49.9 | 53.9 | 34.1 | 61.6 |
| *Limosilactobacillus gastricus gad* | 1404 | 0.809 | 40.5 | 52.4 | 31.8 | 37.4 |
| *Limosilactobacillus coleohominis gad* | 1278 | 0.805 | 41.9 | 48.1 | 35.4 | 42 |
| *Latilactobacillus sakei gad* | 1440 | 0.827 | 45.2 | 50.4 | 36.2 | 49 |
| *Latilactobacillus curvatus gad* | 1356 | 0.783 | 37.8 | 48.5 | 34.3 | 30.8 |
| *Lentilactobacillus parakefiri gad* | 1440 | 0.792 | 38.1 | 48.5 | 33.5 | 32.1 |
| *Lentilactobacillus buchneri gad* | 1449 | 0.795 | 40.2 | 50.5 | 34.4 | 35.6 |
| *Companilactobacillus nuruki gad* | 1401 | 0.752 | 34.4 | 43.3 | 31.3 | 28.7 |
| *Companilactobacillus futsaii gad* | 1410 | 0.789 | 47.2 | 58.1 | 35.1 | 48.5 |
| *Lacticaseibacillus paracasei gad* | 1446 | 0.796 | 40.3 | 51 | 34.4 | 35.5 |
| *Loigolactobacillus rennini gad* | 1437 | 0.822 | 42.2 | 47.2 | 35.1 | 44.5 |
| *Paucilactobacillus suebicus gad* | 1401 | 0.804 | 41.3 | 49 | 32.1 | 42.8 |
| *Furfurilactobacillus rossiae gad* | 1416 | 0.801 | 40.8 | 49.8 | 33.9 | 38.8 |
| *Secundilactobacillus paracollinoides gad* | 1401 | 0.789 | 48.9 | 54.6 | 34.5 | 57.6 |
| *Lactococcus lactis gad* | 1401 | 0.818 | 35.5 | 47.8 | 31.5 | 27.4 |
| *Enterococcus faecium gad* | 1401 | 0.822 | 36.5 | 50.1 | 31.3 | 28.1 |
| *Streptococcus thermophilus gad* | 1380 | 0.781 | 42.3 | 51.1 | 37.6 | 38.3 |
| *Escherichia coli gad-alpha* | 1401 | 0.748 | 54 | 60 | 40 | 61.9 |
| *Escherichia coli gad-beta* | 1401 | 0.749 | 53.3 | 59.5 | 40 | 60.4 |
|  |  |  |  |  |  |  |

**Supplementary Table 17-A:** Codon adaptation (CAI) index and codon position %GC estimates for GAD system genes in examined bacterial species.

**Supplementary Table 17-B:** Codon adaptation (CAI) index and codon position %GC estimates for GAD system genes in examined bacterial species.

| **Name** | **length** | **CAI** | **%GC** | **%GC1** | **%GC2** | **%GC3** |
| --- | --- | --- | --- | --- | --- | --- |
| *Levilactobacillus brevis gadC* | 1506 | 0.796 | 45.5 | 45.8 | 38.2 | 52.4 |
| *Levilactobacillus zymae gadC* | 1506 | 0.793 | 45.7 | 45.8 | 38.2 | 53 |
| *Levilactobacillus senmaizukei gadC* | 1500 | 0.765 | 43.9 | 48 | 38.8 | 44.8 |
| *Levilactobacillus tangyuanensis gadC* | 1500 | 0.767 | 43.6 | 47.2 | 39.4 | 44.2 |
| *Levilactobacillus_angrenensis gadC* | 1506 | 0.791 | 44.3 | 45.4 | 38.6 | 48.8 |
| *Levilactobacillus cerevisiae gadC* | 1506 | 0.775 | 42.3 | 45.6 | 39.4 | 41.8 |
| *Levilactobacillus spicheri gadC* | 1506 | 0.802 | 46.6 | 47 | 39.4 | 53.4 |
| *Lactiplantibacillus plantarum yjeM* | 1485 | 0.802 | 46.6 | 43.8 | 39 | 57 |
| *Lactiplantibacillus argentoratensis yjeM* | 1485 | 0.801 | 46.5 | 43.8 | 39 | 56.8 |
| *Lactiplantibacillus paraplantarum yjeM* | 1485 | 0.805 | 44.8 | 43.6 | 39 | 51.7 |
| *Limosilactobacillus reuteri gadC* | 1533 | 0.766 | 38 | 44.4 | 36.6 | 33.1 |
| *Limosilactobacillus fermentum yjeM* | 1506 | 0.745 | 53.4 | 44.4 | 40.2 | 75.5 |
| *Limosilactobacillus oris gadC* | 1566 | 0.752 | 50 | 51.5 | 38.9 | 59.6 |
| *Limosilactobacillus antri gadC* | 1566 | 0.766 | 49.4 | 50.6 | 39.5 | 58 |
| *Limosilactobacillus gastricus yjeM* | 1506 | 0.792 | 44 | 42.6 | 40 | 49.4 |
| *Limosilactobacillus coleohominis gadC* | 1470 | 0.789 | 41.6 | 43.9 | 38.2 | 42.9 |
| *Latilactobacillus_sakei gadC* | 1506 | 0.793 | 45.7 | 45.8 | 38.2 | 53 |
| *Latilactobacillus curvatus gadC* | 1458 | 0.765 | 39.4 | 44.2 | 39.5 | 34.6 |
| *Lentilactobacillus parakefiri gadC* | 1347 | 0.764 | 38.1 | 45.4 | 37.6 | 31.2 |
| *Lentilactobacillus buchneri gadC* | 1521 | 0.742 | 40.5 | 45.8 | 39.1 | 36.7 |
| *Companilactobacillus nuruki yjeM* | 1530 | 0.809 | 36.5 | 41.2 | 39.8 | 28.4 |
| *Companilactobacillus futsaii yjeM* | 1527 | 0.778 | 38.2 | 41.3 | 38.3 | 35.2 |
| *Loigolactobacillus rennini gadC* | 1518 | 0.804 | 43.3 | 44.3 | 38.9 | 46.6 |
| *Furfurilactobacillus rossiae GadC* | 1605 | 0.783 | 40.7 | 45 | 38.9 | 38.1 |
| *Secundilactobacillus paracollinoides yjeM* | 1488 | 0.778 | 49.1 | 41.7 | 42.7 | 62.7 |
| *Lactococcus lactis gadC* | 1512 | 0.752 | 35.4 | 44.8 | 35.5 | 25.8 |
| *Enterococcus faecium gadC* | 1512 | 0.753 | 37.5 | 48.4 | 36.3 | 27.8 |
| *Streptococcus thermophilus gadC* | 1434 | 0.767 | 42.5 | 46.7 | 39.1 | 41.6 |
| *Escherichia coli gadC* | 1536 | 0.759 | 48.2 | 52.5 | 39.5 | 52.5 |
|  |  |  |  |  |  |  |
