# Supplementary material for: Molecular evolution and population genetics of glutamate decarboxylase acid resistance pathway in lactic acid bacteria
Source: Front Genet. 2023 Jan 26;14:1027156. doi: 10.3389/fgene.2023.1027156 (PMC9909107; doi:10.3389/fgene.2023.1027156)
Supplement: Supplementary file 2 [file DataSheet1.PDF]

Supplementary Figure Legends

Supplementary Figure 1. Nonsynonymous nucleotide diversity estimates of GAD pathway genes from individual (A) *L. brevis*, and (B) *L. plantarum* populations isolated from different environments. Thick arrows show the GAD pathway gene organiation in L. brevis and L. plantarum genomes. Numbers inside the thick arrows show the length of each gene in base pairs (bp). Numbers outside the arrows indicate the relative nucleotide number (nt) positions of genes with respect to each other.

gadR: Transcription regulator gene; *gadC* and *yjeM*: glutamate/GABA transporter gene; *gad1*, *gad2*, and *gadB*: glutamate decarboxylase enzyme gene. *L. brevis* operon includes *gadR*, *gadC*, and *gad1*.

Sample sizes for *L. brevis* groups:
Fermented vegetable: 5, Sourdough: 4, Fermented dairy: 5, Fermented beverage: 10, Feces: 5, all samples:30

Sample sizes for *L. plantarum* groups:
Kimchi: 28, Unspecified food: 8, Raw food: 4, Kefir: 9, Cheese:10, Beverage: 6, Meat: 6, Feces: 5, other milk-based fermented products: 12, all samples:88

Supplementary Figure 2. Phylogenetic trees based on *L. brevis* (A) *gadR* , (B) *gadC* , (C) *gad1* and *gad2* gene sequences from isolation sources indicated on the trees. The *gad1-gad2* tree colored based on the separation of *gad1* and *gad2* sequence clades. The trees were constructed with MEGA-X using the Maximum Likelihood method and the Kimura 2-parameter model. One thousand replicates were used to generate the bootstrap consensus tree. A discrete Gamma distribution was used for evolutionary rate variations across sites. Numbers on the nodes show the bootstrap support, numbers on the branches show substitutions per site.

Supplementary Figure 3. Phylogenetic trees based on *L. plantarum* (A) *gadB* , (B) *yjeM* gene sequences from isolation sources indicated on the trees. The trees were constructed with MEGA-X using the Maximum Likelihood method and the Kimura 2-parameter model. One thousand replicates were used to generate the bootstrap consensus tree. A discrete Gamma distribution was used for evolutionary rate variations across sites. Numbers on the nodes show the bootstrap support, numbers on the branches show substitutions per site.

Supplementary Figure 4. Amino acid sequence alignment of Gad1, Gad2, and GadB proteins. Grey, yellow, and pruple highlighted residues show N-terminal, PLP-binding, and Small domain regions, respectively. Amino acid sequences are represented by their accession numbers.

Supplementary Figure 5. Secondary structure predictions of *L. brevis* and *L. plantarum* GAD enzyme proteins (Gad1, Gad2, GadB).

Supplementary Figure 6. Amino acid sequence alignment of GadC and YejM transporter proteins.

Supplementary Figure 7. Secondary structure and trans-membrane domain predictions for *L. brevis* GadC and L. plantarum YjeM transporter proteins

Supplementary Figure 8. Secondary structure, trans-membrane domain, and 3D structure predictions for 191 amino acid (aa) long *L. brevis* GadR regulatory protein. Sliding-window analyses show nonsynonymous nucleotide diversity (π) and Tajima's *D* test results throughout the gene.

Supplementary Figure 9. Unrooted phylogenetic trees based on (A) *gad* , (B) transporter (*gadC* and *yejM*) gene sequences from 32 bacterial species. The trees were constructed with MEGA-X using the Maximum Likelihood method and General Time Reversible (GTR) model. One thousand replicates were used to generate the bootstrap consensus tree. A discrete Gamma distribution was used for evolutionary rate variations across sites. Numbers on the nodes show the bootstrap support, numbers on the branches show substitutions per site. Orange color represents species where GAD pathway genes are organized in an operon structure. Purple color represents species without an operon structure, and blue color represents species with a small operon structure consisting of only a transporter and a GAD gene.

Supplementary Figure 1

A

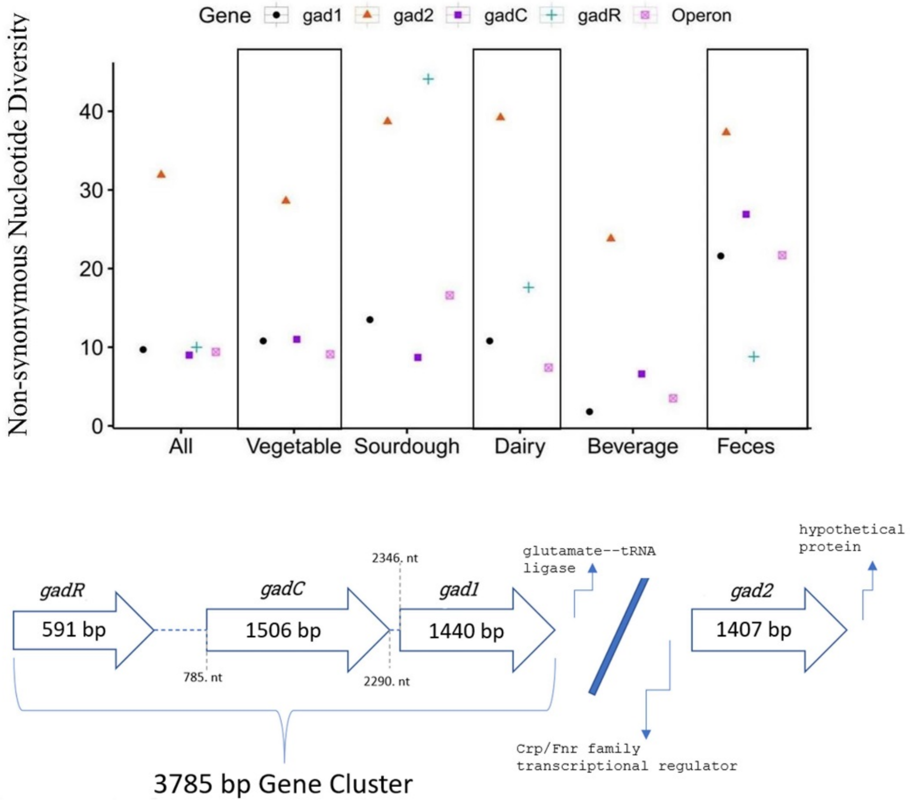

Levilactobacillus brevis

B

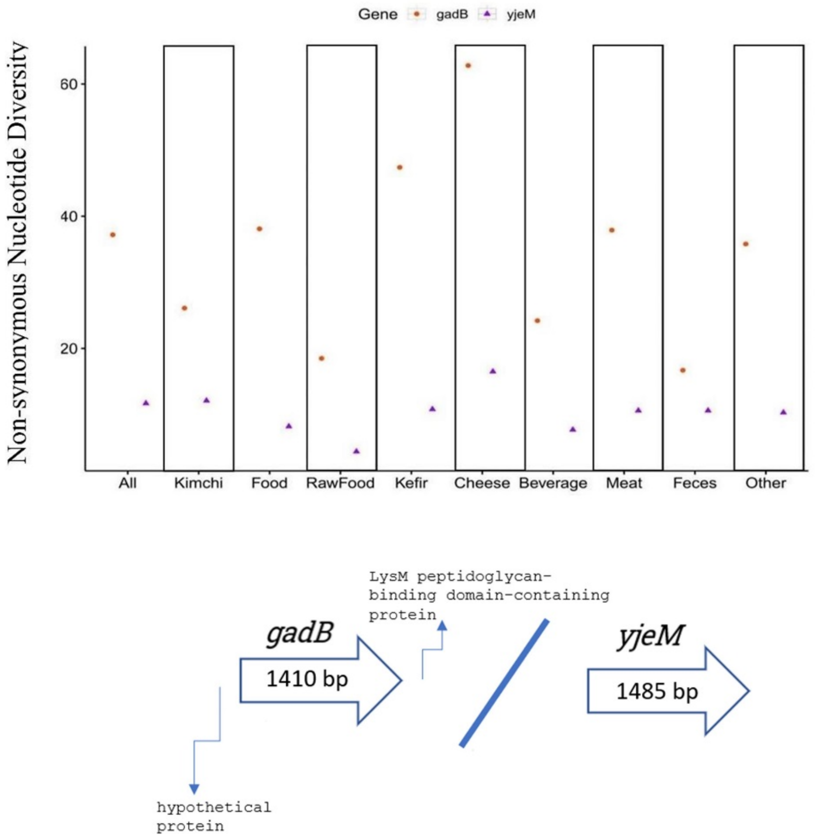

Lactiplantibacillus plantarum

Supplementary Figure 2

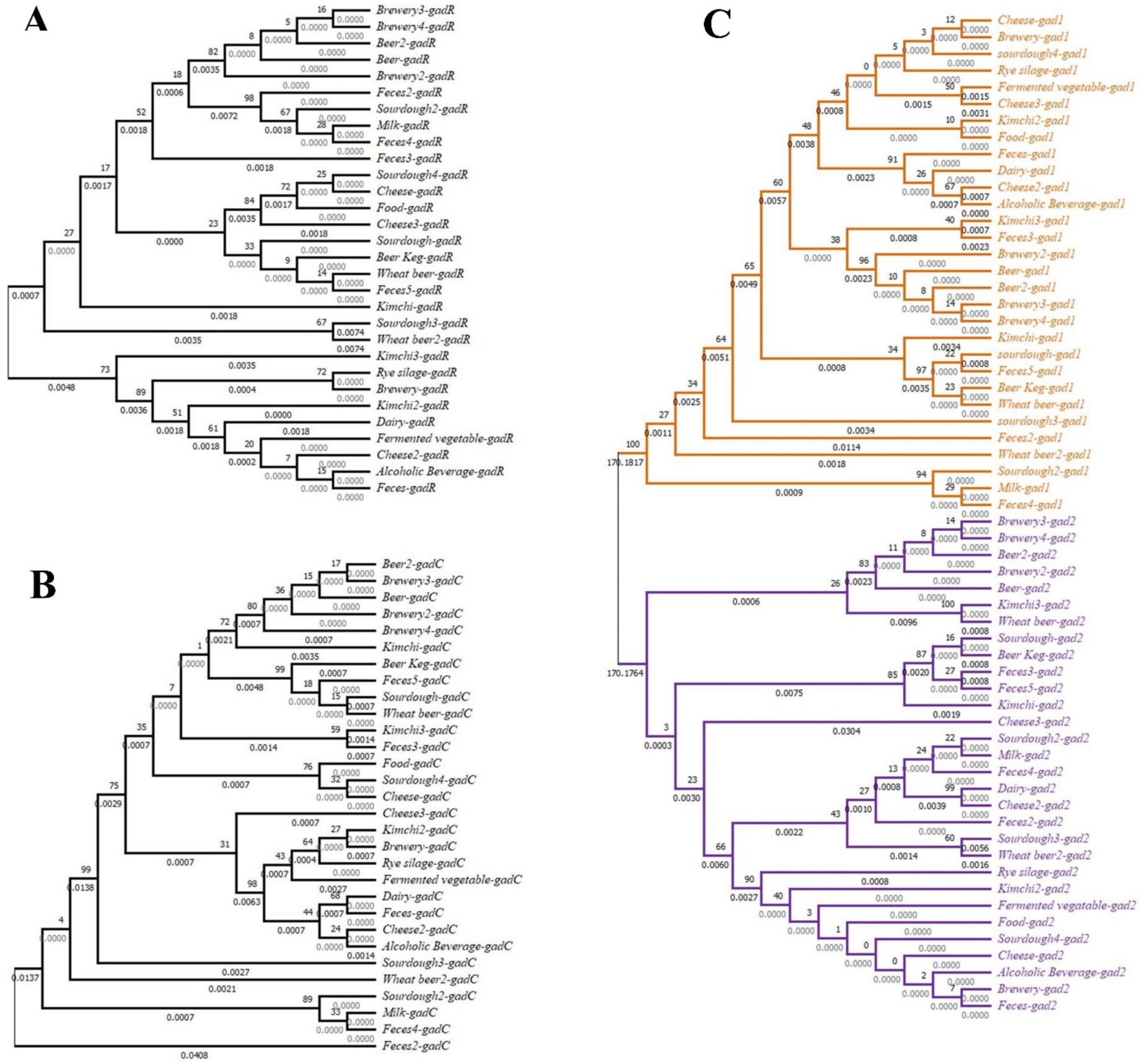

Supplementary Figure 3

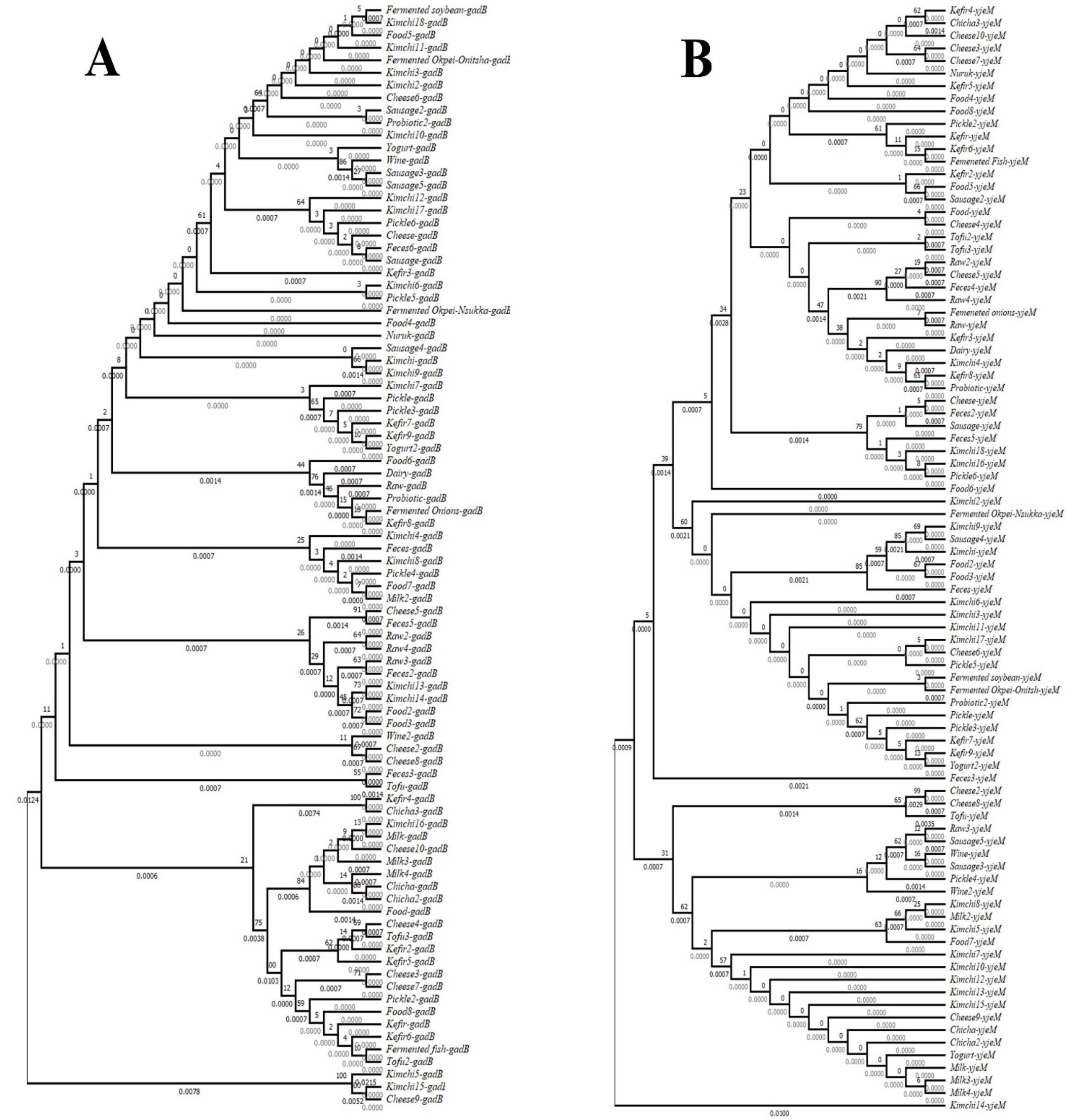

Supplementary Figure 4

```
ANN50035.1      -----MINNVDLEKTF---LGSVEAGQSLPTNTLPDDPMAPDVAAQLVEHYRLNEAKANQ
ANN49747.1      MAMLYGKHTHETDETLTPIFGASAERHDLPKYKLAKHALEPREADRLVRDQLLDEGNSRL
ATI72654.1      MAMLYGKHNHEAEEYLEPVFGAPSEQHDLPKYRLPKHSLSPREADRLVRDELLDEGNSRL
                       :. : :: :   :*:    :.**.  *.. .: *  * .**    *:*.::.

ANN50035.1      NLATFCTTQMEPQADELMKNALNTNAIDKSEYPKTAAMENYCVSMIAHLWGIPDNEKIYD
ANN49747.1      NLATFCQTYMEPEAVELMKDTLEKNAIDKSEYPRTAEIENRCVNIIANLWHAPEAE----
ATI72654.1      NLATFCQTYMEPEAVELMKDTLAKNAIDKSEYPRTAEIENRCVNIIANLWHAPDDE----
                ****** * ***:* ****::* .*********.** :** **.:**:**  *: *

ANN50035.1      DFIGTSTVGSSEGCMLGGLALLHSWKHRAKAAGFDIEDLHSHKPNLVIMSGYQVVWEKFC
ANN49747.1      SFTGTSTIGSSEACMLAGLAMKFAWRKRAKANGL---DLTAHQPNIVISAGYQVCWEKFC
ATI72654.1      HFTGTSTIGSSEACMLGGLAMKFAWRKRAQAAGL---DLNAHRPNLVISAGYQVCWEKFC
                 * ****:****.***.***: .:*.:**:* *:   ** :*.**:** :**** *****

ANN50035.1      TYWNVEMRQVPINGDQVSLDMDHVMDYVDENTIGIIGIEGITYTGSVDDIQTLDNLVSEY
ANN49747.1      VYWDIDMHVVPMDDDHMSLNVDHVLDYVDDYTIGIVGIMGITYTGQYDDLARLNAIVERY
ATI72654.1      VYWDVDMHVVPMDEQHMALDVNHVLDYVDEYTIGIVGIMGITYTGQYDDLAALDKVVTHY
                .**:::*. **:: ::::*:::**:****: ****:** ******. **:  *: :*  *

ANN50035.1      N-KTATMPVRIHVDAAFGGLFAPFVDGFNPWDFRLKNVVSINVSGHKYGMVYPGLGWIVW
ANN49747.1      N-RTTKFPVYIHVDAASGGFYTPFIEPELKWDFRLNNVISINASGHKYGLVYPGVGWVIW
ATI72654.1      NHQHPKLPVYIHVDAASGGFYTPFIEPQLIWDFRLANVVSINASGHKYGLVYPGVGWVVW
                * . ..:** ****** **:::**::    ***** **:***.******:****:**::*

ANN50035.1      RHNTADILPAEMRFQVPYLGKTVDSIAINFSHSGAHISAQYYNFIRFGLSGYKTIMQNVR
ANN49747.1      RDQ--QYLPKELVFKVSYLGGELPTMAINFSHSASQLIGQYYNFIRFGFDGYREIQEKTH
ATI72654.1      RDR--QFLPPELVFKVSYLGGELPTMAINFSHSAAQLIGQYYNFIRFGMDGYREIQTKTH
                * .  : ** *: *:*.***  : ::*******.::: .*********:.**. *  :..

ANN50035.1      KVSLKLTAALKTYGIFDILVDGSQLPINCWKLADDAPVGWTLYDLESELAKYGWQVPAYP
ANN49747.1      DVARYLAKSLTKLGGFSLINDGHELPLICYELTADSDREWTLYDLSDRLLMKGWQVPTYP
ATI72654.1      DVARYLAAALDKVGEFKMVNNGHQLPLICYQLAPREDREWTLYDLSDRLLMNGWQVPTYP
                .*:  *: :* . * *.:: :* :**: *::*:      ******.. *   *****:**

ANN50035.1      LPKNRDDVTISRIVVRPSMTMTIADDFLDDLKLAIDGLNHTFGVTTTVDQDNKTTVRS
ANN49747.1      LPKNMADRVIQRIVVRADFGMSMAHDFIDDLTQAIHDLDQAHIVFHSDPQPKKYGFTH
ATI72654.1      LPANLEQQVIQRIVVRADFGMNMAHDFMDDLTKAVHDLNHAHIVYHHDAAPKKYGFTH
                ** *  : .*.*****..: *.:* **:***. *: .*:::. *       :*  .
```

N-terminal domain · PLP-binding domain · Small domain

ANN50035.1 *L. brevis* Gad1 / ANN49747.1 *L. brevis* Gad2 / ATI72654.1 *L. plantarum* GadB

Supplementary Figure 5

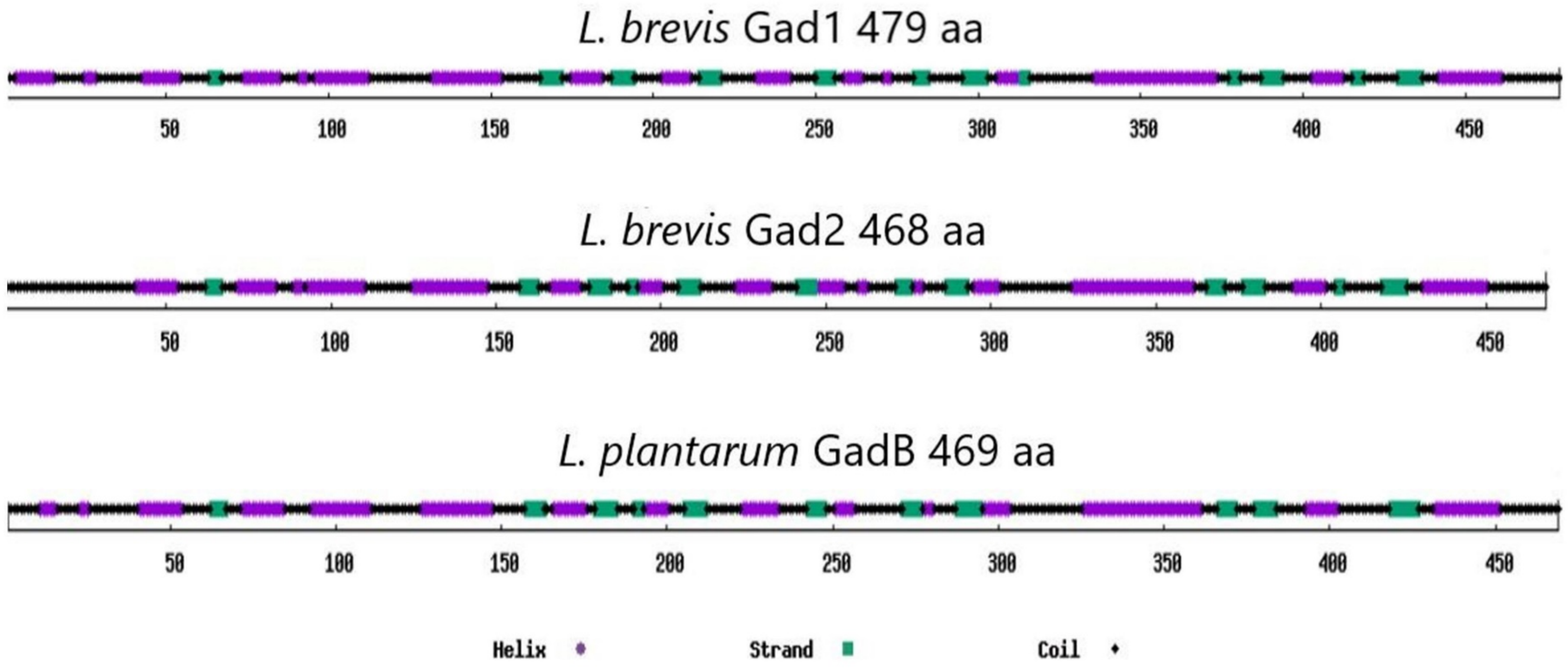

Supplementary Figure 6

```
GadC            MDENKSEQQIDTQKANRITGFQLFSMTTSMVMTVYGFA----AFAKQGPTAFFYLFLAGI
YjeM            ---------MATKDNEKIT---LMALVMMIFTTVFGFANSTVAYYLMGYSSILFYLVAAV
                         : *:. :.**   *:::.  :. **:***    *:   * ::::: ::*.:

GadC            LWFLPVTRTSGEMASIDGWSKGGIFTWSRNMLGERAG------WSALFYQWIHITVGMNT
YjeM            LFFIPFALMMAEFGAAVKSDSSGMYKWLEVSVNAKFAFVGTFMWFASYIIWL-VSTSAKV
                *:*:*.:   .*:.:    ...*::.*    :. . .      * * :  *: ::.. :.

GadC            MMYFIIGCFS-------VTFGLPIITDNPLVKFILMMIILWGLI--FLQQKGTSVTGKIA
YjeM            WIPFTTIFFGSDQTQRFAMFGLNA------TQMIGILSCLWMVLVTFVSIKGMKGIVRVT
                 : *    *.       . ***        .::* ::  ** ::  *:. ** .   .::

GadC            QW-CFTLGVIIPVFFLLFLFILYLVQGNPAMIHVNMHTIFPSSWNGSV-LVGFVPF-ILA
YjeM            SLGGLAVTSLTAILLVVSGVVLALNHGQFAQPLQHVMTSPNPSYQHPVGLLGFAVFAIFA
                .   :::  : .:::::  .:* * :*: *    :: *   .*:: .* *:**. * *:*

GadC            FAGAEGSAPHVKDLDKPS-IYPK--------VMMALAVAAICSDIIGSMAIAMTIPNNQI
YjeM            YGGLEVLGGMVDKTKNPEKTFPRGIIISAIVITLGYGLGIFCWGISTNWQAVLSNPTTNL
                :.* *  .  *.. .:*.  :*.        : :. .:. :* .*  .   .:: *..::

GadC            -QLSNGIVYAYGALVAR-YGVGV-------VFVEKLTGFLLAVGVLGEISSWVVGPNAGM
YjeM            GNISYVMMQNLGYVLGQAFGLSTAAAKTMGLWFARYTGLGMFLAYSGAFFTLTYSPLKTL
                 ::*  ::   * ::.. :*:..       ::. . **: : :.  * : : . .*   :

GadC            FEAAKAGYLPPRFSKANKYGIETNVMVLQGVIVSIVGALLTFGAGGNKASLSFQTAMSLT
YjeM            ILGTPKELWPKKFTKLNKAGMPSYAMMVQCAIVIVIILVASFATADASAFYNVLTLMA-N
                : .:     * .*:* ** *: : .*::* .** ::  : :*.:.. .*  .. * *: .

GadC            VALYTLMYMLMFISYLVLQFKYVDLHRDFVAAKS-RWLRITYGILGFILSAFGFVVTFFP
YjeM            VSM-TLPYLFLLYAFPKFK-ENQNIVKPFEVYKSLTWTKIISWVV-FIVVLGANVFTLIQ
                *:: ** *:::: ::  :: :  :: . * . **  * .*   :: **:   . *.*::

GadC            PADLSVASKHTYLMLLVSAFVVMLVLPFILYRFHDKWALQLGVNVDEVAAPTGHEALEKE
YjeM            PI-LETGQIQNTIWMLVGP-IVFGVAGIIWYQVRERH-----VN----------------
                *  *.... :. : :**.. :*: *  :* *...:..     **

GadC            TK
YjeM            --
```

Supplementary Figure 7

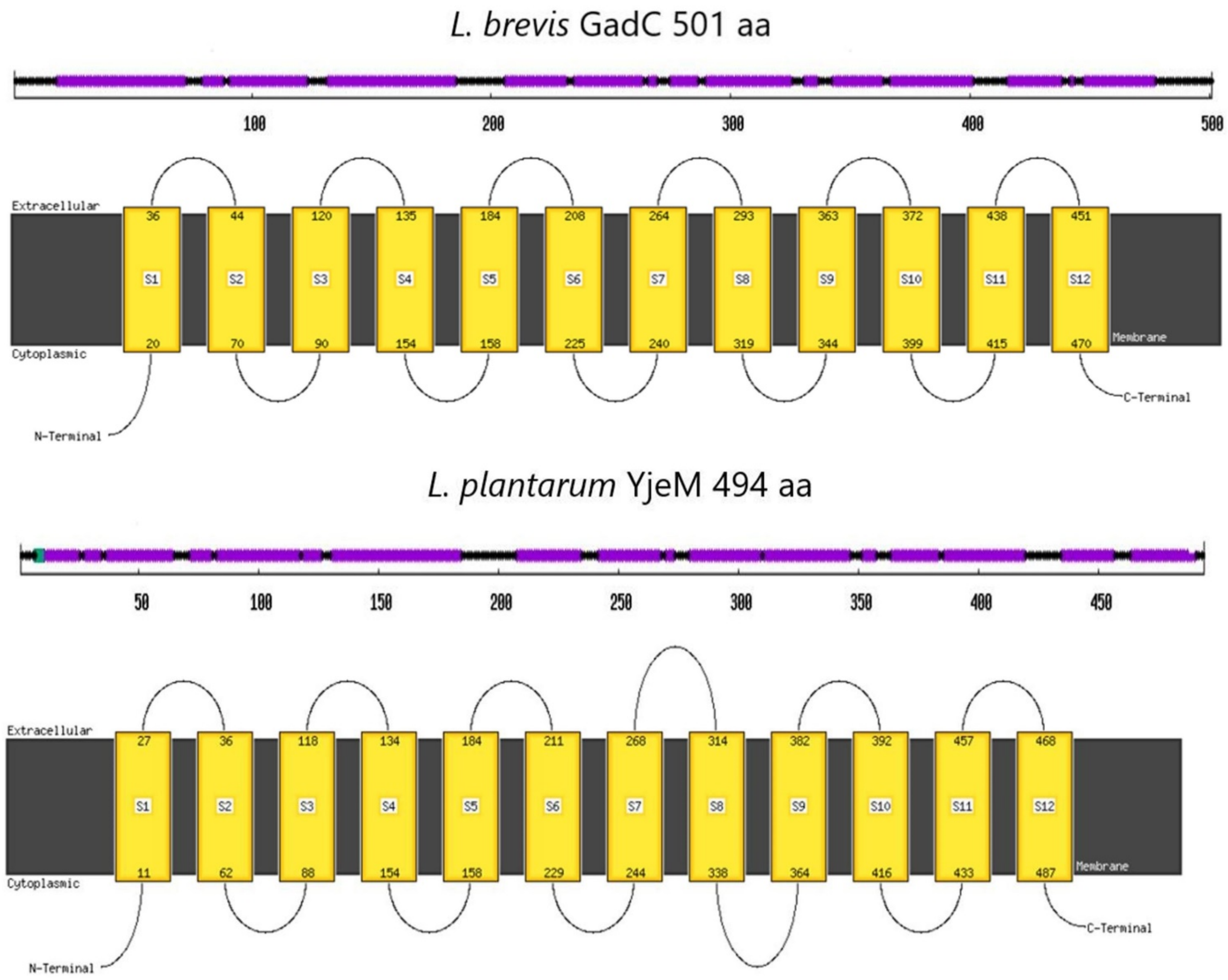

Supplementary Figure 8

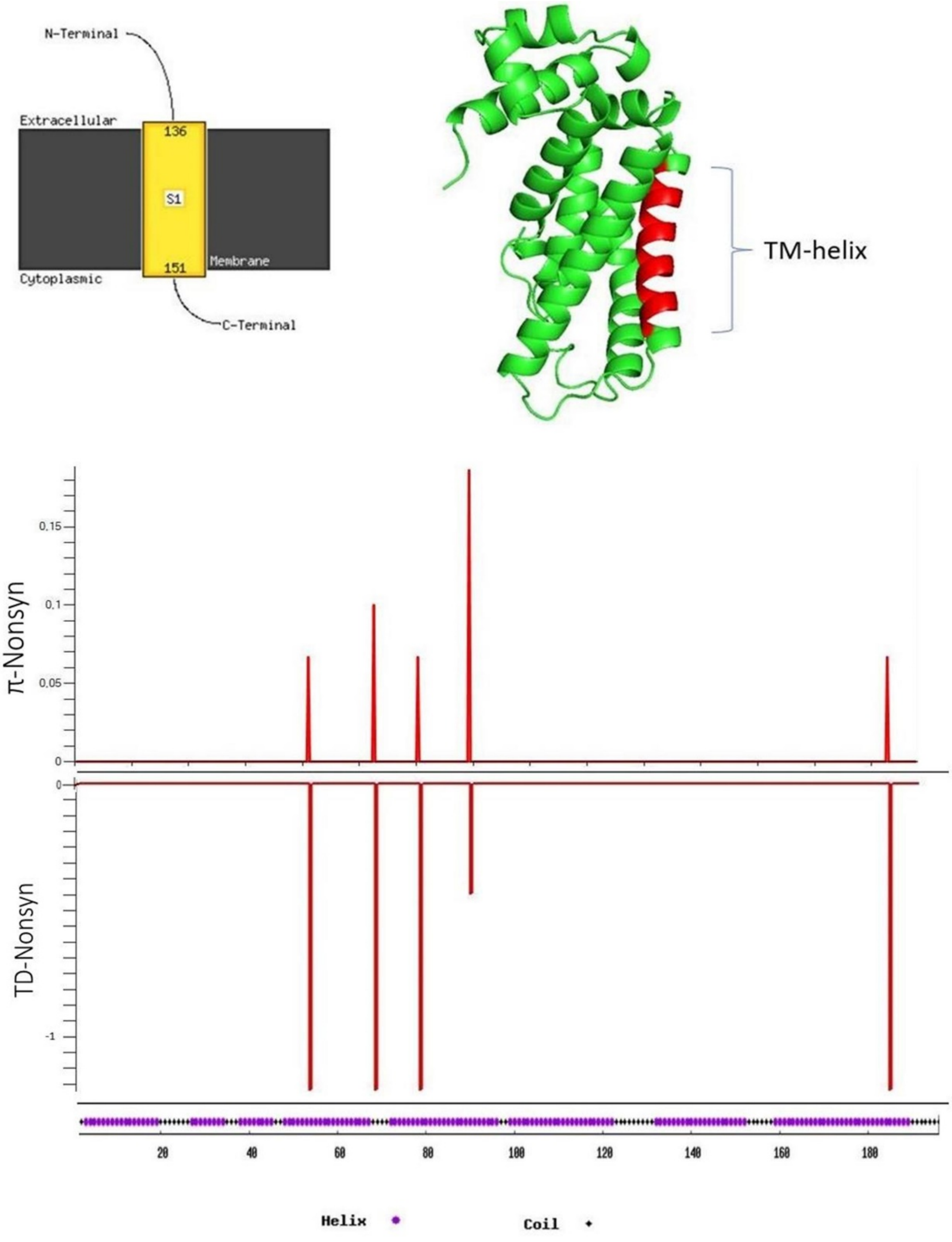

L. brevis GadR 191 aa

Supplementary Figure 9

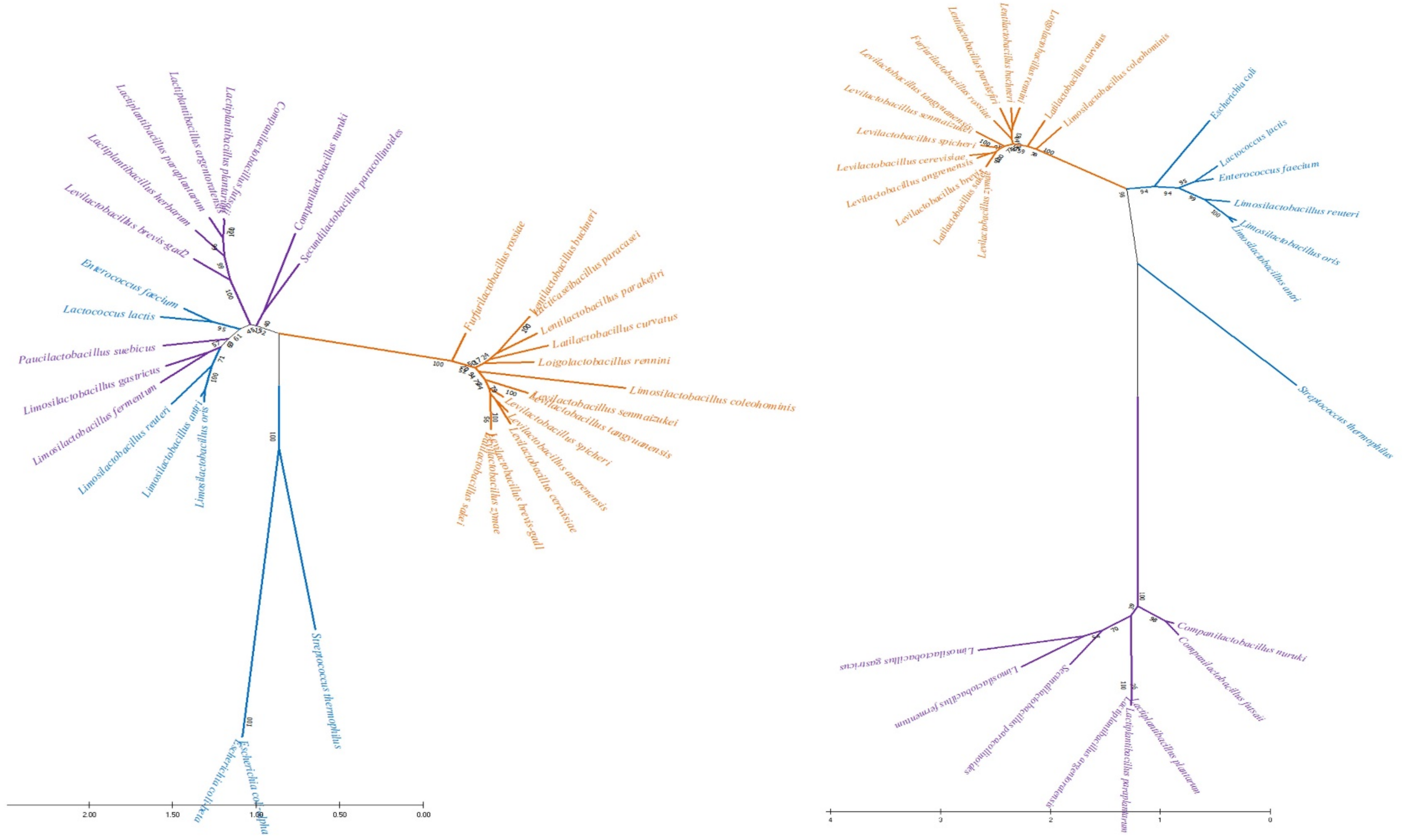

A

B
